# Endothelial cell glycogen synthase kinase 3**β** promotes lipotoxic endotheliopathy and liver inflammation in MASH

**DOI:** 10.1172/jci.insight.202552

**Published:** 2026-05-05

**Authors:** Akitoshi Sano, Qianqian Guo, Khaled Warasnhe, Chady Meroueh, Nantawat Satthawiwat, Asma Hamdi, Ghefar Hmaydoosh, Xin Dai, Usman Yaqoob, Kevin D. Pavelko, Charlene Miciano, Tatiana Kisseleva, Zeba Firdaus, Patrick P. Starlinger, David Pereyra, Enis Kostallari, Petra Hirsova, Davide Povero, Samar H. Ibrahim

**Affiliations:** 1 Division of Gastroenterology & Hepatology, Mayo Clinic, Rochester, Minnesota, USA.; 2 Division of Gastroenterology, Tohoku University Graduate School of Medicine, Sendai, Japan.; 3 Department of Laboratory Medicine and Pathology, Mayo Clinic, Rochester, Minnesota, USA.; 4 Department of Biochemistry, Medical Biochemistry Program, Faculty of Medicine, Chulalongkorn University, Bangkok, Thailand.; 5 Division of Pediatric Gastroenterology & Hepatology, Mayo Clinic, Rochester, Minnesota, USA.; 6 Department of Gastroenterology and Hepatology, Tianjin Medical University General Hospital, Tianjin, China.; 7 Immune Monitoring Core and; 8 Department of Immunology, Mayo Clinic, Rochester, Minnesota, USA.; 9 Department of Cellular and Molecular Medicine and; 10 Center for Epigenomics, UCSD School of Medicine, La Jolla, California, USA.; 11 Department of Surgery, UCSD, La Jolla, California, USA.; 12 Department of Biochemistry and Molecular Biology and; 13 Division of Hepatobiliary and Pancreas Surgery, Department of Surgery, Mayo Clinic, Rochester, Minnesota, USA.; 14 Centre of Physiology and Pharmacology and; 15 Department of General Surgery, Medical University of Vienna, Vienna, Austria.

**Keywords:** Hepatology, Vascular biology, Endothelial cells, Mitochondria, Monocytes

## Abstract

In metabolic dysfunction–associated steatohepatitis (MASH), liver sinusoidal endothelial cells (LSECs) acquire a proinflammatory phenotype termed lipotoxic endotheliopathy. We previously identified glycogen synthase kinase 3β (GSK3β) as a central signaling hub in LSECs during MASH. To elucidate the molecular mechanisms and functional outcome of lipotoxicity-induced GSK3β activation in LSECs, we utilized endothelial cell–specific *Gsk3β*-KO (*Gsk3β*^ΔEnd^) mice fed MASH-inducing diets. Endothelial *Gsk3β* deletion significantly reduced markers of lipotoxic endotheliopathy, including adhesion molecules and chemokines, alongside liver injury, inflammation, and fibrosis. Immune profiling via flow cytometry and mass cytometry by time of flight (CyTOF) identified decreased hepatic infiltration of proinflammatory myeloid populations, particularly mature DCs in *Gsk3β*^ΔEnd^ mice. In a coculture system, GSK3β in lipotoxic LSECs promoted DCs maturation. Mechanistically, GSK3 inhibition restored lipotoxicity-induced alterations in LSEC mitochondrial morphology and respiration by regulating AMP-activated protein kinase and dynamin-related protein 1. This rescue suppressed chemokine and adhesion molecule expression, thereby limiting immune cell recruitment. Collectively, under lipotoxic stress, GSK3β amplifies mitochondrial dysfunction and inflammatory signaling in LSECs, enhancing myeloid cell homing and DC maturation. Targeting LSEC GSK3β may, therefore, represent a promising therapeutic strategy to mitigate LSEC-driven fibroinflammatory response in human MASH.

## Abstract 2

Lipotoxic GSK3β activation in LSECs drives mitochondrial dysfunction and fibroinflammation in MASH, while endothelial GSK3β targeting emerges as a promising therapeutic strategy for disease treatment.

## Graphical abstract

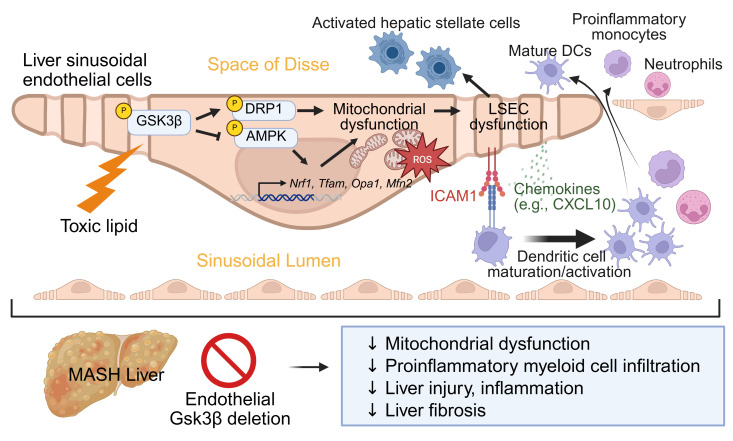

## Introduction

Metabolic dysfunction–associated steatohepatitis (MASH) is a growing public health problem worldwide (1–3). MASH is associated with an increased risk of cardiovascular events, chronic kidney disease, hepatic and extrahepatic malignancies, and liver-related outcomes, including liver failure, and hepatocellular carcinoma (4). In addition, MASH is currently a leading cause of liver transplantation posing a high socioeconomic burden and a global health crisis (5).

The pathogenesis of MASH involves both toxic lipid-induced cellular stress known as lipotoxicity driving lethal and sublethal liver injury (6) and an inflammatory response paired with a dysfunctional reparative process culminating in progressive liver fibrosis (7). The inflammatory response is mainly mediated by recruited proinflammatory myeloid cells and their homing to the liver (8, 9). Chemotaxis and adhesion of myeloid cells to the liver sinusoidal endothelial cells (LSECs) are fundamental elements in the inflammatory response in MASH (10). LSECs under lipotoxic stress undergo structural and functional alterations, leading to LSEC dysfunction and a proinflammatory phenotype, which we refer to as lipotoxic endotheliopathy (11–13). Emerging data implicate LSEC endotheliopathy in liver inflammation in MASH (14, 15). However, few studies have focused on targeting the molecular mediators of LSEC lipotoxic endotheliopathy in MASH.

Glycogen synthase kinase 3β (GSK3β) is a serine/threonine kinase that integrates multiple signaling pathways, including cell metabolism, adhesion, and inflammation (16). Aberrant GSK3β activation is pathogenic in numerous inflammatory diseases, and GSK3 inhibitors dampened the exuberant inflammatory responses in various rodent models (17, 18). Using phosphoproteomics and kinome profiling of primary mouse LSECs from control versus MASH mice, we recently reported LSEC GSK3β as the top signaling hub in MASH (19). However, the subcellular organelles and molecular mechanisms linking lipotoxicity to LSEC endotheliopathy remain unknown.

GSK3 exits in 2 isoforms α and β, which share some redundant biological functions (20). GSK3β is primarily localized in the cytosol, with smaller amounts present in the mitochondria and the nucleus. On the contrary, GSK3α is absent in the mitochondria. This indicates that GSK3β signaling is crucial for mitochondrial dysfunction (20). Importantly, mitochondrial biodynamics modulates the metabolic profile and inflammatory phenotype of endothelial cells (21). Thus, we hypothesize that GSK3β-induced mitochondrial dysfunction enhances LSEC endotheliopathy and promotes liver inflammation and fibrosis in MASH.

Herein, using a combination of endothelial cell–specific *Gsk3β*-KO (*Gsk3β*^ΔEnd^) mouse model with diet-induced MASH and an in vitro model of lipotoxic endotheliopathy, we show that: (a) *Gsk3β*^ΔEnd^ suppresses the infiltration of mature DCs and activated proinflammatory myeloid cells, thereby attenuating liver injury, inflammation, and fibrosis in murine MASH; (b) *Gsk3β*^ΔEnd^ suppresses chemokine and cytokine signaling pathways and markers of lipotoxic endotheliopathy; (c) GSK3β inhibition in LSECs under toxic lipid treatment reduces mitochondrial dysfunction and morphological alterations via modulating AMP-activated protein kinase (AMPK) and dynamin-related protein 1 (DRP1); (d) mitochondrial dysfunction leads to a proinflammatory phenotype in LSECs via NF-κB and cellular MYC (cMYC) activation; and (e) lipotoxic LSECs induce hepatic stellate cell (HSC) activation, and this profibrogenic crosstalk is attenuated by GSK3 inhibition. We validated the clinical relevance of these findings using liver samples from a human metabolic dysfunction–associated steatotic liver disease (MASLD) cohort confirming increased DC abundance with disease progression and precision-cut liver slices (PCLS) derived from patients validating the suppression of LSEC endotheliopathy markers by GSK3 inhibition. These data define GSK3β in LSEC as a signaling hub linking mitochondrial dysfunction and liver inflammation and support the role of GSK3β as a potential therapeutic target in human MASH.

## Results

### Endothelial cell–specific Gsk3β deletion attenuates liver injury without altering the metabolic phenotype in mice with diet-induced MASH.

**Figure 1:**
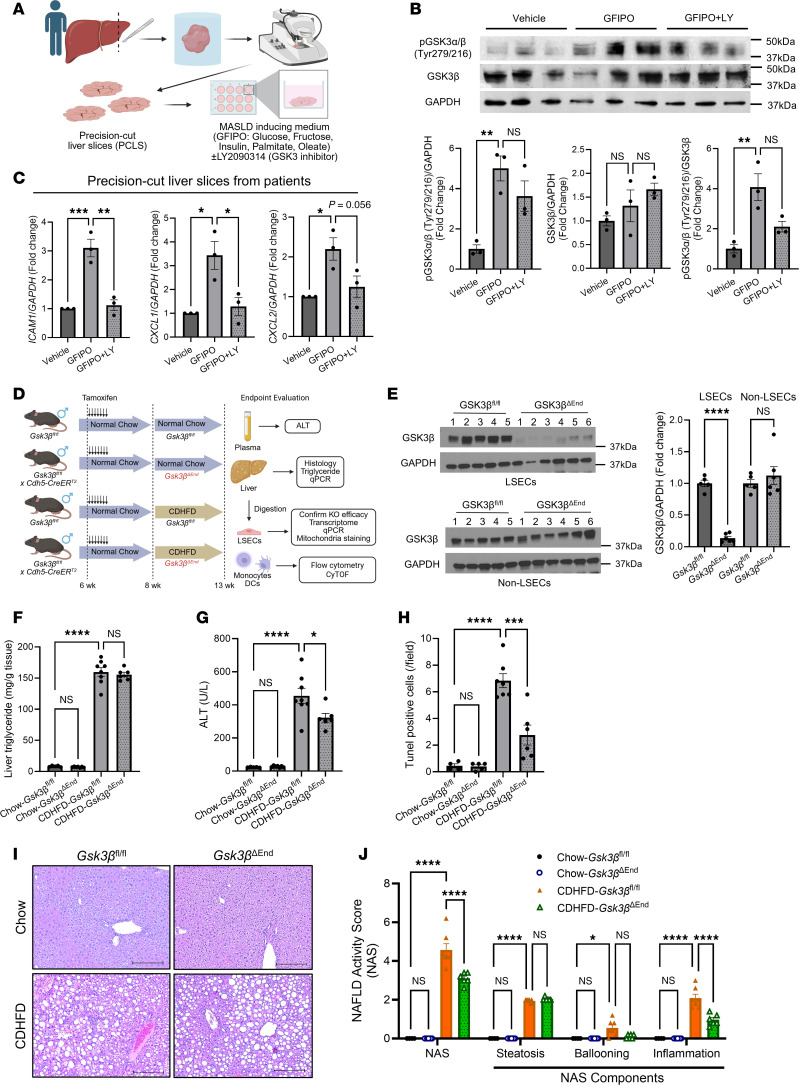
Endothelial cell–specific *Gsk3β* deletion does not alter the metabolic phenotype but attenuates liver injury in murine MASH. (**A**) Schematic representation of precision-cut liver slice treatment with MASLD inducing medium (GFIPO) ± the GSK3 inhibitor LY2090314 (LY). Created in BioRender. (**B**) Western blot analysis of pGSK3α/β (Tyr279/216) and GSK3β protein levels in PCLS treated with GFIPO ± LY (upper panel) and the quantification (lower panel) (*n* = 3). (**C**) mRNA expression of *ICAM1* and *CXCL* family in PCLS. (**D**) Schematic representation of the mouse model and the feeding study. Created in BioRender. (**E**) Western blot analysis of GSK3β protein levels to confirm knockdown efficiency (left panel) and the quantification (right panel). (**F**) Quantification of hepatic triglyceride. (**G**) Serum ALT levels. (**H**) TUNEL^+^ cells (DAPI-stained nuclei overlapped with TUNEL^+^ areas) per field (20×) were counted. (**I**) Representative images of H&E-stained liver sections. Scale bar: 200 μm. (**J**) NAS and its components (Steatosis, Ballooning, and Inflammation). Bar graphs represent the mean ± SEM. *P < 0.05;**P < 0.01; ***P < 0.001; ****P < 0.0001 (1-way ANOVA with Bonferroni’s multiple comparison for **B**, **C**, and **E**–**H**; 2-way ANOVA with Bonferroni’s multiple comparison for **J**).

Publicly available databases (Liver Cell Atlas: https://www.livercellatlas.org) shows that GSK3β is abundantly expressed in human LSECs (Supplemental Figure 1, A–C; supplemental material available online with this article; https://doi.org/10.1172/jci.insight.202552DS1). In LSECs isolated from WT mice, the *Gsk3β* was highly expressed compared with other cell types (Supplemental Figure 1D). Analysis of human single nuclear RNA-seq (GSE244832) (22) from healthy patients, patients with hepatic steatosis (MASL), and patients with MASH shows that GSK3β expression is abundant in LSECs (Supplemental Figure 1E). However, during disease progression to steatosis and MASH, GSK3β mRNA levels in LSECs do not significantly increase. On the other hand, we have previously confirmed the increased activated phosphorylation of GSK3β in the liver of patients with MASH, when compared with those with isolated steatosis, and healthy controls (19). Furthermore, we employed PCLS from patients undergoing hepatobiliary surgery and treated them with MASLD-inducing medium of glucose, fructose, insulin, palmitate, and oleate (GFIPO) (23). We identified upregulation of phosphorylated GSK3α/β (Y279/216) when compared with control medium (Figure 1, A and B). Although the GSK3 inhibitor LY2090314 (LY) is primarily an ATP-competitive inhibitor, it showed a decreasing trend in pGSK3α/β, which may be attributed to the suppression of GSK3 autophosphorylation (24). These findings indicate that GSK3β is mainly regulated posttranslationally, and its activating phosphorylation is increased in MASH. We next measured the mRNA levels of LSEC endotheliopathy markers, intercellular adhesion molecule 1 (ICAM1) and C-X-C chemokine ligands (CXCLs) (12), and we identified increased expressions of these proinflammatory mediators in the GFIPO-treated PCLS, which were attenuated with LY treatment (Figure 1C). These findings collectively indicate that elevated GSK3 activity in a lipotoxic MASLD environment drives the proinflammatory phenotype of LSECs.

To assess the effect of LSEC *Gsk3β* deficiency on the severity of murine MASH, we fed *Gsk3β*^ΔEnd^ mice a choline-deficient high fat diet (CDHFD) (Figure 1D). Deletion of *Gsk3β* in LSECs was confirmed by Western blot on isolated primary mouse LSECs (Figure 1E). Western blot analysis of GSK3β in other liver cells (non-LSECs) showed similar expression levels between *Gsk3β*^ΔEnd^ and *Gsk3β*^fl/fl^ mice confirming endothelial cell–specific deletion (Figure 1E). *Gsk3β*^ΔEnd^ mice on the MASH-inducing CDHFD diet exhibited similar caloric intake (Supplemental Figure 2A), body weight (Supplemental Figure 2B), and liver/body weight ratio (Supplemental Figure 2C) compared with CDHFD-fed *Gsk3β*^fl/fl^ mice. Likewise, fasting blood glucose (Supplemental Figure 2D), HOMA-IR (Supplemental Figure 2E), serum cholesterol (Supplemental Figure 2F), and liver triglyceride (Figure 1F) were similar in CDHFD-fed *Gsk3β*^ΔEnd^ mice compared with *Gsk3β*^fl/fl^ mice. Interestingly, serum alanine aminotransferase (ALT) level was reduced in *Gsk3β*^ΔEnd^ mice (Figure 1G), and TUNEL staining (Figure 1H, a cell death marker) was significantly reduced in the CDHFD-fed *Gsk3β*^ΔEnd^ mice compared with *Gsk3β*^fl/fl^ mice on the same diet. Histological assessment showed similar hepatic steatosis between *Gsk3β*^ΔEnd^ mice and *Gsk3β*^fl/fl^ mice, while inflammatory infiltration was reduced in *Gsk3β*^ΔEnd^ mice, resulting in a lower NAFLD activity score (NAS) (Figure 1, I and J). To validate these results in another murine mouse model, we employed methionine and choline deficient diet–fed (MCD-fed) mice. Although there were not differences in body and liver weight between MCD-fed *Gsk3β*^fl/fl^ and *Gsk3β*^ΔEnd^ mice (Supplemental Figure 2, G and H), endothelial *Gsk3β* deletion significantly decreased ALT level (Supplemental Figure 2I). Histological assessment showed that endothelial *Gsk3β* deletion attenuated hepatic inflammation in MCD-fed mice (Supplemental Figure 2, J and K). Taken together, endothelial cell–specific *Gsk3β* deletion reduced liver injury and inflammation without affecting steatosis in murine MASH.

### Endothelial cell–specific Gsk3β deletion attenuates liver inflammation in mice with diet-induced MASH.

**Figure 2:**
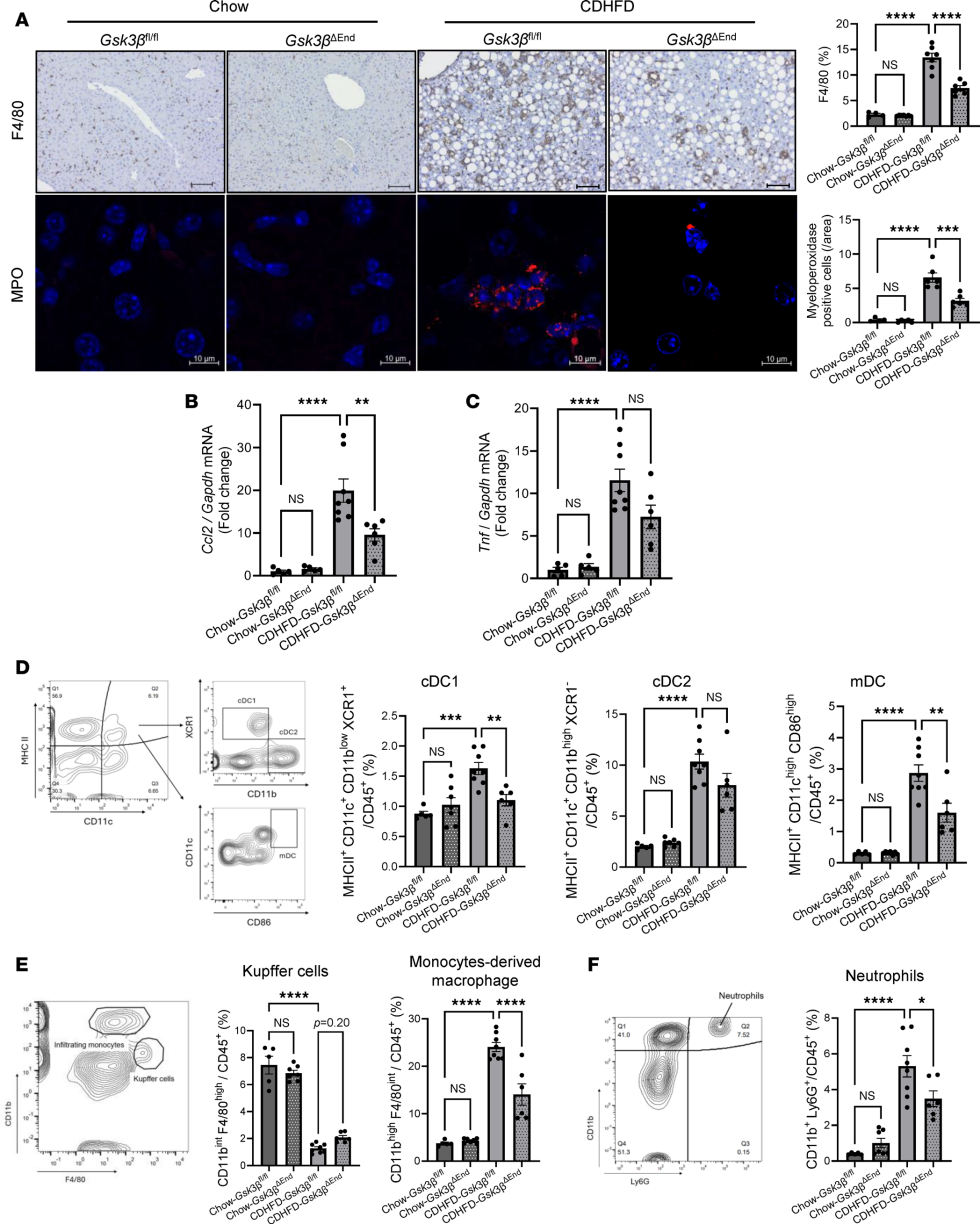
Endothelial cell–specific *Gsk3β* deletion attenuates liver inflammation by suppressing recruitment of proinflammatory myeloid cells in CDHFD-fed mice. (**A**) Representative IHC staining for F4/80 and MPO. Positive areas were quantified in 5 random 10× microscopic fields for F4/80, and MPO^+^ cells were counted in 5 random fields (right panel). Scale bar:100 μm (F4/80); 10 μm (MPO). (**B** and **C**) Whole liver mRNA expression of *Ccl2* (**B**) and *Tnf* (**C**). (**D**) Representative flow cytometry contour plots (left panel) and quantification of cDC1, cDC2, and mDC populations among CD45^+^ intrahepatic mouse leukocytes (right panels). (**E**) A representative flow cytometry contour plots (left panel) and quantification of Kupffer cells and infiltrating monocytes among CD45^+^ cells (right panels). (**F**) A representative flow cytometry contour plots (left panel) and quantification of neutrophils among CD45^+^ cells (right panels). Bar graphs represent the mean ± SEM; *P < 0.05; **P < 0.01; ***P < 0.001; ****P < 0.0001 (1-way ANOVA with Bonferroni’s multiple comparison).

Immunostaining for macrophage marker F4/80 and neutrophil marker myeloperoxidase (MPO) revealed a significant reduction in both markers in the CDHFD-fed *Gsk3β*^ΔEnd^ mice compared with *Gsk3β*^fl/fl^ mice on the same diet (Figure 2A). Additionally, CDHFD-fed *Gsk3β*^ΔEnd^ mice showed reduced whole liver chemokine (C-C motif chemokine ligand 2 [*Ccl2*]) and cytokine (TNF-α [*Tnfa*]) mRNA hepatic expression (Figure 2, B and C). These results were also validated in mice with MCD-induced MASH (Supplemental Figure 3, A–C). We next employed flow cytometry analysis to assess whether *Gsk3β*^ΔEnd^ mice with MASH have reduced proinflammatory myeloid cells hepatic infiltration (Supplemental Figure 3D and Supplemental Table 1). We classified conventional DCs (cDCs) as CD45^+^MHC-II^+^CD11c^+^, which we further subclassified into XCR1^+^CD11b^lo^ cDC1 and XCR1^–^CD11b^hi^ cDC2, and CD11c^hi^CD86^hi^ monocyte-derived DC (mDC) (Figure 2D) (25). We identified increased abundance of cDC1, cDC2, and mDC among CD45^+^ cells in CDHFD-fed mice livers (Figure 2D), which was consistent with published literature (25). Interestingly, *Gsk3β*^ΔEnd^ decreased the proportion of cDC1 and mDC (known to be pathogenic in MASH without significant alteration of cDC2 population; reported to be protective in MASH) (Figure 2D) (25). A similar trend was observed in high-fat fructose and cholesterol–fed (FFC-fed) mice treated with the enteral formulation of the GSK3 inhibitor elraglusib (9-ING-41) (Supplemental Figure 3E). We further examined the monocyte and macrophage cell populations given their established role in promoting chronic liver injury and fibrosis in MASH (26). As previously reported (27, 28), F4/80^hi^CD11b^int^ macrophage population predominates in the chow-fed mice as a surrogate for resident Kupffer cells, and this population was reduced in CDHFD-fed mice, with some restoration in the *Gsk3β*^ΔEnd^ mice (Figure 2E). The CD11b^hi^F4/80^int^ subset, representing recruited monocyte-derived macrophage population (27, 28), was significantly increased in CDHFD-fed *Gsk3β*^fl/fl^ mice and reduced in the *Gsk3β*^ΔEnd^ mice on the same diet (Figure 2E). Furthermore, neutrophil infiltration (CD11b^+^Ly6G^+^) into the liver was also reduced in CDHFD-fed *Gsk3β*^ΔEnd^ mice (Figure 2F).

**Figure 3:**
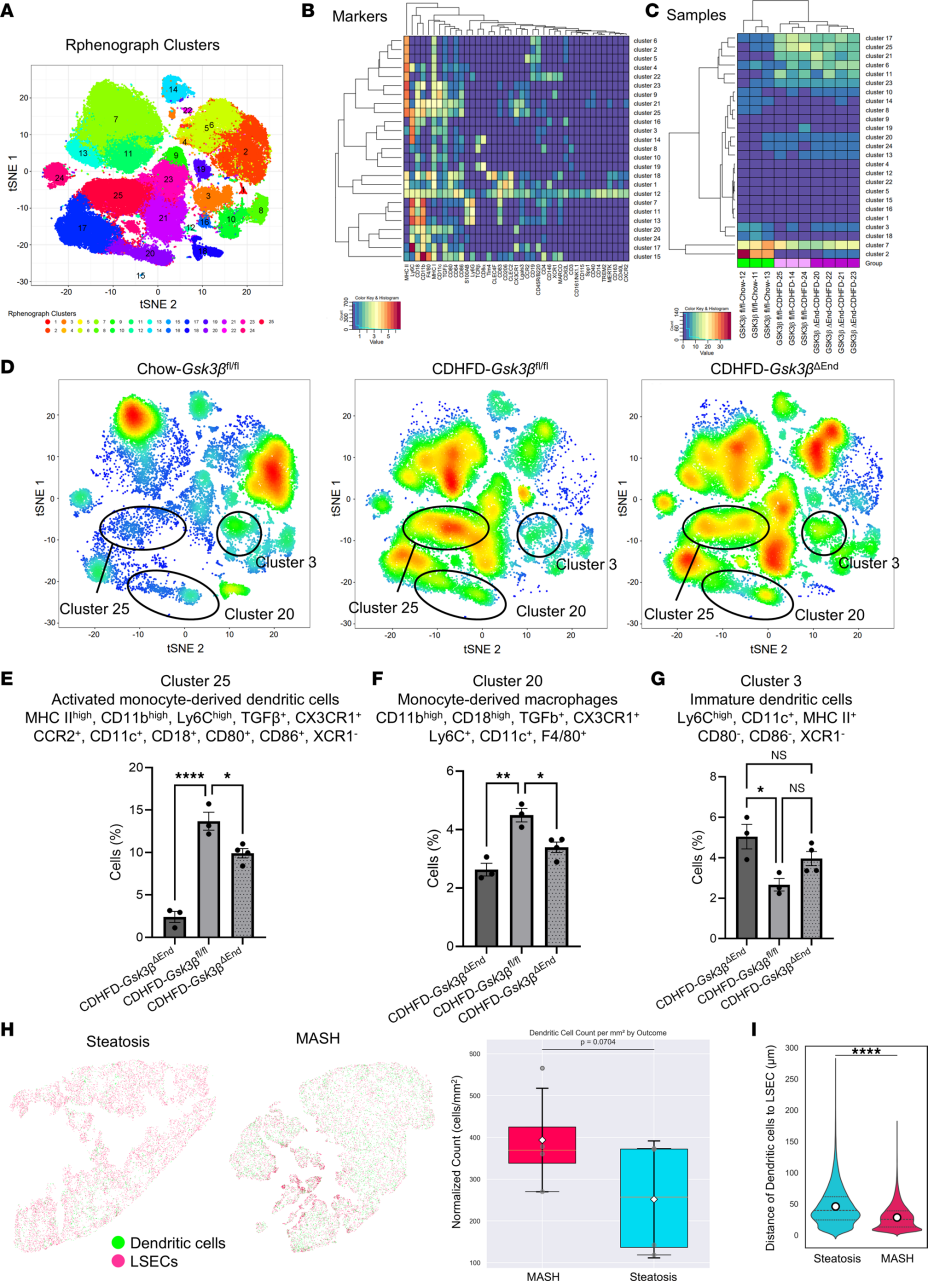
Intrahepatic leukocyte profiling by mass cytometry by time-of-flight (CyTOF). CyTOF was performed on intrahepatic leukocytes from chow-fed *Gsk3β*^fl/fl^ mice, and CDHFD-fed *Gsk3β*^fl/fl^ and *Gsk3β*^ΔEnd^ mice. (**A**) Twenty-five unique clusters were defined by the surface marker panel using the Rphenograph clustering algorithm and visualized via t-distributed stochastic neighbor embedding (tSNE) plot. (**B**) Heatmap showing markers distribution and relative intensity across clusters. (**C**) Heatmap showing the relative abundance of each cluster per mouse. (**D**) Representative tSNE plots for each group. Red indicates high frequency categorization of cells to a cluster. Blue indicates low frequency. (**E**–**G**) Clusters categorization into distinct leukocyte subpopulations based on markers intensities. Proportion of cells belonging to specific clusters were quantified for each experimental group. (**H**) Representative images of digital slide scanning and analysis showing LSECs and DCs in liver tissue sections of patients with simple steatosis and MASH. Immunofluorescence staining of liver sections were analyzed using *QuPath* and *InstanSeg*. DCs were defined by CD45^+^CD11c^+^HLA-DR^+^ and LSECs by CD14^+^ expression. DC number per mm^2^ was counted and shown in the right panel. (**I**) Violin plots showing the distribution of DC-LSEC distances. Spatial distances were calculated with SciPy in Python (*n* = 4 for steatosis and 4 for MASH). Bar graphs represent the mean ± SEM; *P < 0.05; **P < 0.01; ****P < 0.0001 (**E**–**G**: 1-way ANOVA with Bonferroni’s multiple comparison, **H**: unpaired *t* test, **I**: Mann-Whitney *U* test).

To assess how endothelial Gsk3β deletion influences the intrahepatic immune landscape, including lymphoid and myeloid populations, we applied mass cytometry by time-of-flight (CyTOF) on intrahepatic leukocytes (IHLs) isolated from chow-fed *Gsk3β*^fl/fl^ mice and CDHFD-fed *Gsk3β*^fl/fl^ and *Gsk3β*^ΔEnd^ mice. The IHL were clustered into 25 different clusters (Figure 3, A and B) based on the CyTOF panel of 42 different metal conjugated antibodies (Supplemental Figure 4, A–D, and Supplemental Table 2). Each group of mice displayed a characteristic pattern of clusters abundance (Figure 3, C and D). Each cluster was identified based on the marker profile (Supplemental Table 3 and Supplemental Figure 5).

Monocyte-derived DC/macrophage populations (Cluster 25 and 20, respectively) were increased in CDHFD-fed *Gsk3β*^fl/fl^ and decreased in *Gsk3β*^ΔEnd^ mice on the same diet (Figure 3, E and F). Cluster 20 is consistent with monocyte-derived macrophage (CD11b^hi^, CD18^hi^, TGF-β^+^, CX3CR1^+^, Ly6C^+^, CD11c^+^, F4/80^+^) (29), whereas Cluster 25 showed fully mature, MHC-II^hi^ monocyte-derived DCs (MHC-II^hi^, CD11b^hi^, Ly6C^hi^, TGF-β^+^, CX3CR1^+^, CCR2^+^, CD11c^+^, CD18^+^, with high costimulatory [CD80^+^, CD86^+^]) (30) and fibrogenesis potential; transforming growth factor beta (TGF-β)^+^ (31). CD11c CD18 (integrin αXβ2), also known as complement receptor 4, is a well-established marker of DCs, particularly activated and mature DCs (32, 33). On the other hand, the population of CD80^–^/CD86^–^ immature DCs (Cluster 3) was reduced in CDHFD-fed *Gsk3β*^fl/fl^ mice, with no significant difference observed between chow-fed mice and CDHFD-fed *Gsk3β*^ΔEnd^ mice (Figure 3G), supporting the association between endothelial *Gsk3β* deletion and reduced intrahepatic mature DC infiltration in murine MASH. Furthermore, we identified Cluster 12 as lipid-associated macrophages (LAMs), characterized by high expressions of TREM2, SPP1, and Lgals3 (Supplemental Figure 5E and Supplemental Table 3), which has been known to maintain the fibrotic niche during MASH progression (34). While this population represented a small fraction of total IHLs, we observed that it was enriched in MASH mouse livers and showed a downward trend in *Gsk3β*^ΔEnd^ mice, although it did not reach statistical significance. Taken together, the reduction in these fibrogenic macrophages may contribute to the attenuated fibrosis in *Gsk3β*^ΔEnd^ mice fed a MASH-inducing diet.

Three B cell clusters (Cluster 2, 5, and 6) showed an increasing trend in *Gsk3β*^ΔEnd^ mice (Supplemental Figure 5, M–O). These populations displayed high expression of MHC-II and canonical B cell markers (CD19, B220) together with low levels of costimulatory molecules (CD80/CD83) and TGF-β, consistent with naïve or low-activation states. B cells with low costimulatory molecules and high MHC-II are less likely to promote T cell activation and may even include regulatory B cells with the capacity to secrete IL-10 and suppress inflammation (35, 36). Thus, the enrichment of these B cell populations in *Gsk3β*^ΔEnd^ mice may contribute to a more tolerogenic intrahepatic environment. No significant difference was observed in the remaining myeloid and lymphoid cells clusters between CDHFD-fed *Gsk3β*^fl/fl^ and *Gsk3β*^ΔEnd^ mice (Supplemental Figure 5).

To evaluate the infiltration of DCs defined as (CD45^+^CD11c^+^HLA-DR^+^) in relation to LSEC in human MASH, digital slide scanning, and analysis were performed. Compared with liver tissues from patients with isolated steatosis, MASH livers showed a higher number of DCs (Figure 3H). The lack of statistical significance likely reflects the inclusion of all DCs in the analysis without accounting for their subtypes. Notably, the distance between DCs and LSECs was significantly reduced in patients with MASH compared with steatosis (Figure 3I). This increased proximity suggests that direct cell-cell contact and chemotactic signaling between LSECs and DCs may contribute to DC maturation, highlighting a potential mechanistic link in MASH. To further validate our findings, we performed coimmunostaining using an LSEC marker (CD31) and DC markers (HLA-DR and CD11c) on liver sections from patients with MASLD and identified increased hepatic DC infiltration in patients with MASH when compared with healthy controls and those with isolated steatosis (Supplemental Figure 6A). These results confirm the close spatial proximity of LSECs and DCs and the increase in DC hepatic infiltration with disease progression in MASLD.

Taken together, these data indicate that endothelial cell–selective *Gsk3β* deletion attenuates MASH progression likely by suppressing hepatic proinflammatory myeloid cells recruitment, and maturation.

### Endothelial GSK3β deletion attenuates lipotoxicity-induced cytokine and chemokine-related pathways in murine MASH.

**Figure 4:**
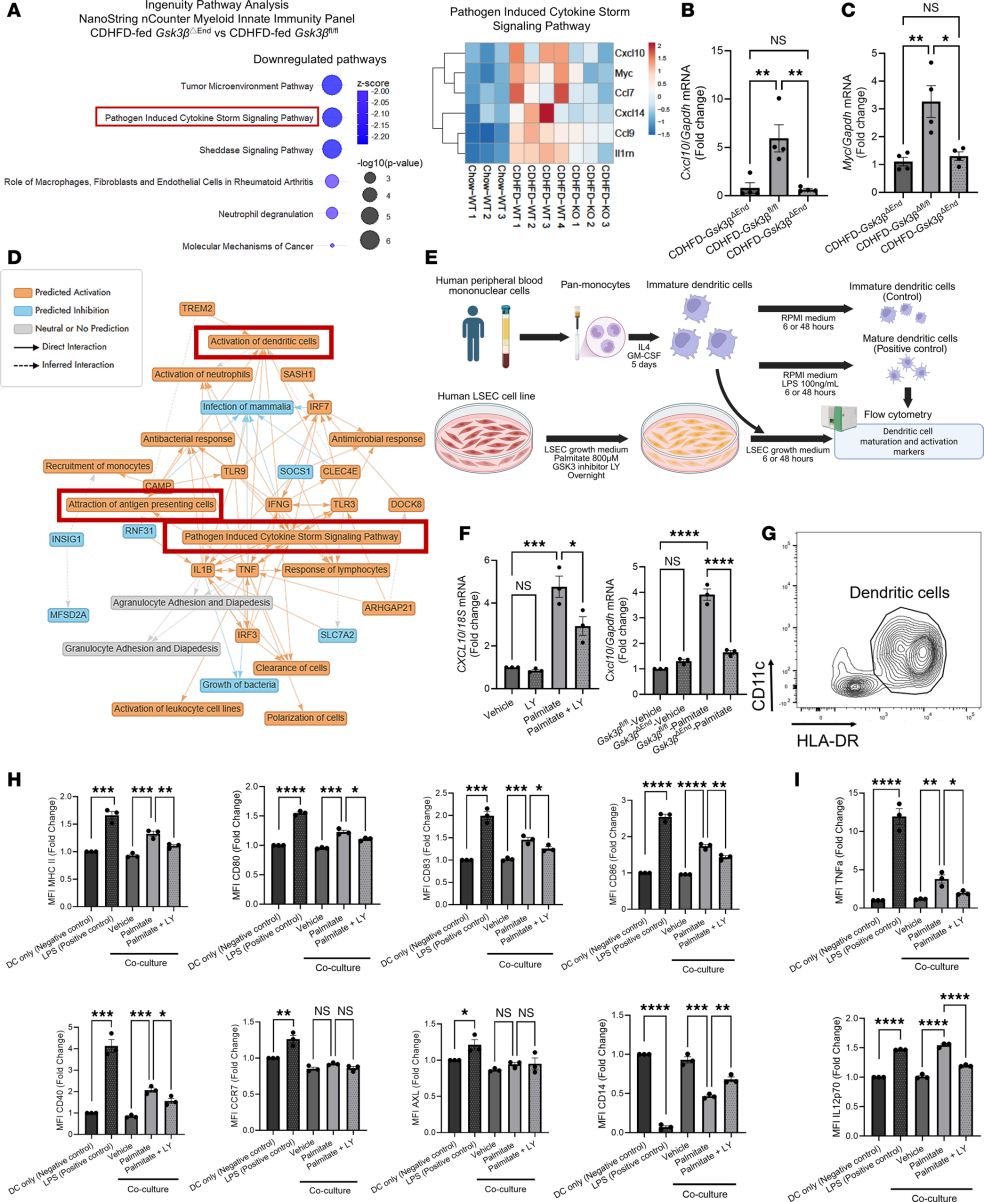
GSK3β inhibition alters cytokine and chemokine–related pathways in hLSECs under lipotoxic stress. (**A**) Bubble chart of top 10 downregulated pathways in CDHFD-fed *Gsk3β*^ΔEnd^ compared with CDHFD-fed *Gsk3β*^fl/fl^ mice (left panel). Differential gene expression data based on Nanostring data and Ingenuity Pathway Analysis. The analysis included downregulated genes with a *P* value of less than 0.05. The color of the bubbles represents the *z* score, while the bubble size reflects the *P* value. Heatmap was generated based on normalized mRNA expression for genes within the Pathogen Induced Cytokine Storm Signaling Pathway (right panel). (**B** and **C**) mRNA expression of *Cxcl10* (**B**) and *Myc* (**C**) in LSECs isolated from mice. (**D**) Network diagram from Ingenuity Pathway Analysis of LSECs from FFC-fed MASH mice (GEO accession no. GSE164006). (**E**) Schematic representation of hLSECs-DCs coculture assay. Created in BioRender. (**F**) *CXCL10* mRNA expression in hLSECs (left panel) treated with palmitate ± GSK3 inhibitor LY and LSECs from mouse models treated with palmitate (right panel). (**G**) Gating strategy for DCs. (**H** and **I**) Flow cytometry analysis of mean fluorescence intensity (MFI) of surface markers (**H**) and intracellular staining (**I**). Bar graphs represent the mean ± SEM; *P < 0.05; **P < 0.01; ***P < 0.001; ****P < 0.0001 (1-way ANOVA with Bonferroni’s multiple comparison).

To gain a comprehensive insight into the effect of *Gsk3β* deletion on the proinflammatory phenotype of LSECs under lipotoxic stress, we performed transcriptomic analysis using NanoString nCounter Myeloid Innate Immunity Panel on isolated LSECs from chow and CDHFD-fed *Gsk3β*^fl/fl^ and *Gsk3β*^ΔEnd^ mice. The volcano plot shows markedly reduced expression of matrix metallopeptidase 13 (*Mmp13*) in CDHFD-fed *Gsk3β*^ΔEnd^ mice compared with CDHFD-fed *Gsk3β*^fl/fl^ mice (Supplemental Figure 7A). This expression was validated by qPCR (Supplemental Figure 7B). MMP13, the functional murine homologue of human MMP1, plays a dual role in liver fibrogenesis transient upregulation during the early phase of injury and contributes to extracellular matrix remodeling and release of profibrogenic mediators (37). Therefore, the suppression of *Mmp13* in *Gsk3β*-deficient mice likely reflects attenuation of the fibrogenic response, preventing the reactive elevation in MMP13 normally observed during liver injury. Furthermore, Ingenuity Pathway Analysis (IPA) showed downregulation of Pathogen Induced Cytokine Storm Signaling Pathway in CDHFD-fed *Gsk3β*^ΔEnd^ mice compared with CDHFD-fed *Gsk3β*^fl/fl^ (Figure 4A). Consistently, the proinflammatory mediators in this pathway, including *Cxcl10* and *Myc*, were upregulated in CDHFD-fed *Gsk3β*^fl/fl^ mice and reduced in the *Gsk3β*^ΔEnd^ mice (Figure 4, A–C). CXCL10 is known to recruit and activate CXCR3-expressing effector T cells and Tregs as well as DCs (38), while cMYC modulates the immunemetabolic pathways and drives lipid metabolic reprogramming, including the direct upregulation of fatty acid elongases, thereby linking inflammatory responses with altered lipid metabolism (39). Furthermore, Kyoto Encyclopedia of Genes and Genomes (KEGG) pathway enrichment analysis identified Chemokine Signaling and Cytokine-Cytokine Receptor Interaction Pathways as highly enriched in the CDHFD-fed *Gsk3β*^ΔEnd^ mice versus CDHFD-fed *Gsk3β*^Δfl/fl^ mice (Supplemental Figure 7C). In summary, these findings indicate that endothelial Gsk3β deletion mitigates lipotoxic stress–induced proinflammatory and profibrotic responses in LSECs through downregulation of the cytokine and chemokine signaling pathways including *Cxcl10* and *Myc* signaling.

Likewise, network diagram based on differentially expressed genes on the transcriptomic analysis of LSECs isolated from the liver of control mice and mice with FFC diet–induced MASH (GSE164006) identified marked enrichment of pathways related to cytokine signaling, antigen-presenting cell recruitment, and DC activation in LSECs from the mice with MASH (Figure 4D), supporting a role of LSEC under lipotoxic stress in DCs activation across different MASH mouse models. Notably, CXCL10 has been recognized in the vaccine literature as a key mediator of DC maturation (40).

To confirm that direct interaction between LSECs under lipotoxic stress promotes DC maturation and activation, we established a coculture system of hLSECs and differentiated human DCs (Figure 4E). *CXCL10* expression was elevated in palmitate-treated LSECs but was markedly reduced by GSK3β inhibition with LY (Figure 4F) and by Gsk3β deletion (Figure 4F). In addition, the MASLD-inducing medium increased *CXCL10* expression in PCLS derived from patients, which was attenuated by LY treatment (Supplemental Figure 7D), although it did not reach statistic differences, likely because RNA was derived from whole tissue rather than LSEC alone. We then assessed DC maturation markers by flow cytometry (Figure 4G). In the LSEC-DC direct coculture assay, DC maturation markers (MHC-II, CD80, CD83, CD86, CD40) except for AXL and CCR7 were significantly increased when DCs were cocultured with LSECs previously exposed to lipotoxic stress and suppressed in the presence of the pharmacological GSK3 inhibitor LY (Figure 4H). Cytokine markers TNF-α and IL-12p70 showed similar trends (Figure 4I). Although LPS enhanced AXL and CCR7 expressions in DCs, they were not significantly increased in coculture with palmitate-treated LSECs. The differential expression of CCR7 and AXL may be explained by the difference between the myeloid differentiation primary response 88– dependent (MyD88-dependent) and TIR-domain–containing adapter-inducing interferon-β–dependent (TRIF-dependent) signaling pathways in DCs (41). Direct stimulation of DCs with LPS activates both the MyD88 and TRIF pathways. While the MyD88/NF-κB axis drives the production of proinflammatory cytokines (TNF-α, IL12) and the upregulation of CD40 and CD83, the TRIF/IRF3 axis induces the secretion of Type I IFNs (e.g., IFN-β). Because AXL is known to be an IFN-Stimulated Gene (ISG), and CCR7 expression and function relies on autocrine or paracrine Type I IFN signaling, direct LPS stimulation successfully upregulates both markers (41). Our data support that, when DCs are cocultured with lipotoxic LSECs, the MyD88/NF-κB pathway is activated in the DCs. This is supported by the upregulation of CD80, CD83, CD86, CD40, TNF-α, and IL-12p70. However, the endogenous signals derived from palmitate-treated LSECs seem insufficient to trigger the TRIF/Type I IFN pathway in DCs. Taken together, LSECs under lipotoxic stress likely induces a selective MYD88 activation in DCs, which is highly proinflammatory but lacks full migratory capabilities (CCR7) and regulatory feedback mechanisms (AXL).

To assess the role of CXCL10 in DC maturation, human monocyte–derived DCs were cultured with recombinant CXCL10, and the surface marker expression was analyzed by flow cytometry. We confirmed the expression of CXCL10 receptor CXCR3 (38) on human DCs, which was further enhanced upon CXCL10 stimulation. CXCL10 stimulation downregulated CD14 expression and modestly upregulated the activation markers MHC-II and CD86; however, CD80 expression remained unchanged (Supplemental Figure 7E). These findings suggest that CXCL10 contributes partially to DC maturation, whereas additional factors, such as LSEC-DC direct contact, play a complementary role. Furthermore, we employed conditioned medium from lipotoxic LSECs (Supplemental Figure 7F). The conditioned medium from palmitate-treated LSECs induced a similar trend in maturation markers and proinflammatory cytokines as seen in direct coculture (Supplemental Figure 7, G and H). However, this induction was notably weaker than observed in the direct contact model. These data suggest that, while LSEC-derived secretory factors like CXCL10 contribute to DC activation, direct LSEC-DC physical interaction is essential for DC maturation.

Taken together, these data indicate that endothelial GSK3β inhibition attenuates lipotoxicity-induced cytokine and chemokine-related pathways, as well as direct contact between DCs and LSECs, thereby reducing DC maturation and activation in MASH.

### GSK3β inhibition in LSEC under lipotoxic stress reduces lipid and atherosclerosis signaling.

**Figure 5:**
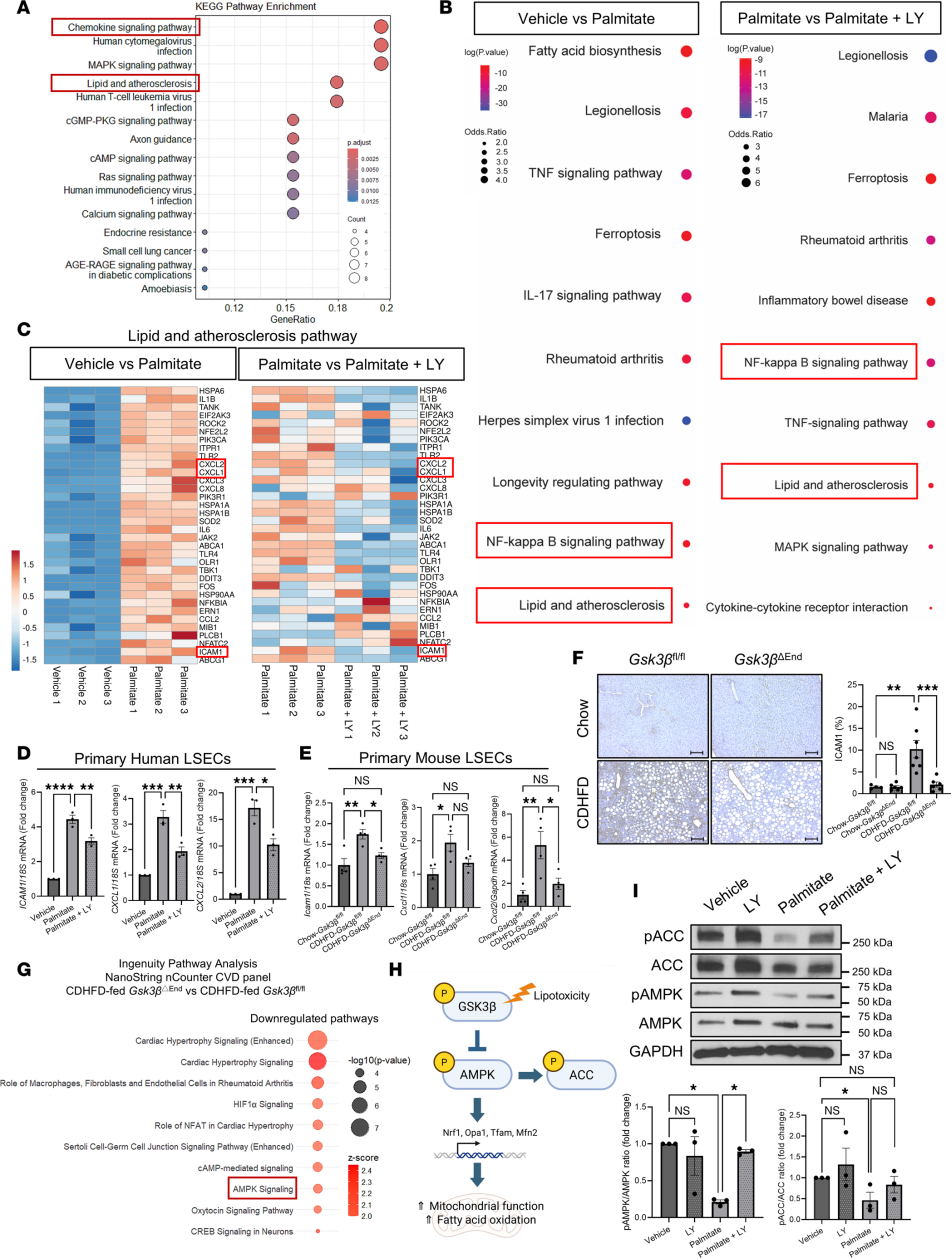
GSK3β inhibition attenuates the LSEC proinflammatory phenotype and upregulates AMPK pathway in lipotoxicity and murine MASH. (**A**) KEGG pathway enrichment analysis of differentially expressed genes in LSECs isolated from CDHFD-fed *Gsk3β*^ΔEnd^ and *Gsk3β*^fl/fl^ mice. The analysis was conducted using R for differential genes (*P* < 0.05) obtained from Nanostring nCounter. The horizontal axis shows the gene ratio, and the vertical axis shows the names of enriched pathways. The bubbles size reflects the number of differentially expressed genes per pathway (count), and the color gradient denotes the *P* value. (**B**) The top 10 enriched pathways in palmitate-treated hLSECs versus vehicle (left panel) and palmitate-treated hLSECs ± LY (right panel) based on transcriptomic analysis. (**C**) Heatmap of genes within the Lipid and Atherosclerosis Pathway in **B**. (**D** and **E**) mRNA expression of *ICAM1*, *CXCL1*, and *CXCL2* in palmitate-treated hLSECs ± LY and isolated primary mouse LSECs. (**F**) Representative immunohistochemical staining for ICAM1. Positive areas were quantified in 5 random 10x microscopic fields. Scale bar: 100 μm. (**G**) Bubble chart of the top 10 upregulated pathways in CDHFD-fed *Gsk3β*^ΔEnd^ versus CDHFD-fed *Gsk3β*^fl/fl^ mice, derived from differential gene expression Nanostring data analyzed via Ingenuity Pathway Analysis (IPA). The analysis included downregulated genes with a *P* value of less than 0.05. The color of the bubbles represents the *z* score, while the bubble size reflects the *P* value. (**H**) Schematic diagram illustrating the relationship between AMPK and mitochondrial function. Created in BioRender. (**I**) Representative Western blot of phosphorylated and total ACC phosphorylated and total AMPK, and GAPDH in hLSECs treated with palmitate ± LY. (left panel). Quantification of pAMPK/AMPK and pACC/ACC density ratios is shown in the right panels, respectively. Bar graphs represent the mean ± SEM; *P < 0.05; **P < 0.01; ***P < 0.001; ****P < 0.0001 (1-way ANOVA with Bonferroni’s multiple comparison).

To elucidate the mechanisms of GSK3β*-*induced lipotoxic endotheliopathy in MASH, we analyzed the transcriptomic data of LSECs isolated from CDHFD-fed mice using the Nanostring nCounter CVD Pathophysiology Panel. KEGG pathway analysis identified enrichment of chemokine signaling pathway and lipid and atherosclerosis pathway (Figure 5A). Furthermore, we performed unbiased transcriptomic analyses using bulk RNA-seq on hLSECs treated with palmitate ± GSK3 inhibitor LY or DMSO (GSE309912). Pathway analysis identified enrichment of lipid and atherosclerosis and NF-κB pathway in both the palmitate versus vehicle and palmitate versus palmitate + LY datasets (Figure 5B). Impaired lipid metabolism in vascular endothelial cells induces atherosclerosis, leading to systemic hypertension, endothelial dysfunction, and the activation of proinflammatory signaling pathways (42, 43). The lipid and atherosclerosis pathway included the adhesion molecule ICAM1 and CXCL family (Figure 5C). We then confirmed that expressions of these genes were increased in palmitate-treated hLSECs and reduced with GSK3 inhibition by qPCR for *ICAM1*, *CXCL1*, *CXCL2* (Figure 5D). We also validated mRNA expression of these genes in LSECs isolated from mice and confirmed that *Icam1* and *Cxcl2* were significantly increased in CDHFD-fed *Gsk3β*^fl/fl^ mice and reduced in the *Gsk3β*^ΔEnd^ mice (Figure 5E). In addition, these gene expressions were reduced in palmitate-treated LSECs isolated from *Gsk3β*^ΔEnd^ mice compared with *Gsk3β*^fl/fl^ mice (Supplemental Figure 8A). Furthermore, we identified a significant reduction of ICAM1 in the CDHFD-fed *Gsk3β*^ΔEnd^ mice compared with *Gsk3β*^fl/fl^ mice on the same diet by immunostaining (Figure 5F). These findings indicate that GSK3β enhances the proinflammatory phenotype of LSECs by modulating lipid metabolism and atherosclerosis-related pathway.

AMP-activated protein kinase (AMPK) pathway was markedly enriched in LSECs from CDHFD-fed *Gsk3β*^ΔEnd^ mice on Nanostring CVD panel transcriptomic analysis (Figure 5G). AMPK activation depends on Thr172 phosphorylation within its α-subunit kinase domain (44) and regulates genes involved in mitochondrial biogenesis and dynamics (Figure 5H) (45, 46). We observed reduced AMPKα (Thr172) phosphorylation in palmitate-treated hLSECs (Figure 5I), which was restored by GSK3β inhibition with LY. AMPK also phosphorylates and inactivates acetyl-CoA carboxylase (ACC), limiting fatty acid synthesis and promoting oxidation. Notably, phosphorylated ACC was significantly downregulated in palmitate-treated hLSECs compared with controls (Figure 5I). Given mitochondria’s vital role in metabolism, we next examined GSK3β’s contribution to mitochondrial dysfunction in LSECs under lipotoxic stress.

### GSK3 inhibition mitigates mitochondrial structural and functional impairments in LSECs under lipotoxic stress.

**Figure 6:**
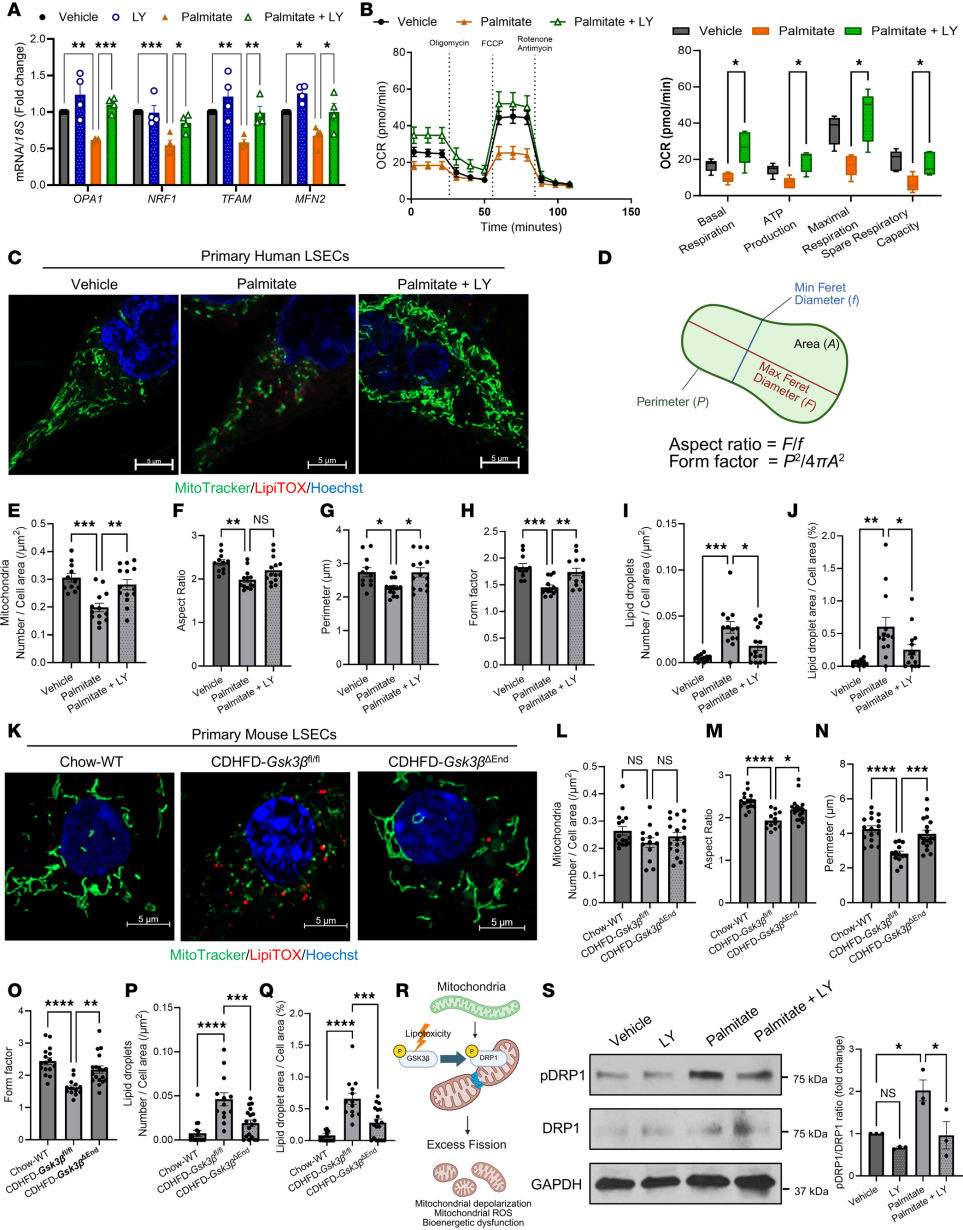
GSK3 inhibition protects LSEC against lipotoxicity-induced mitochondrial morphological and functional alterations. (**A**) mRNA expression of *OPA1*, *NRF1*, *TFAM*, and *MFN2* in hLSECs with palmitate ± GSK3 inhibitor LY. (**B**) hLSECs were treated with 500 μM palmitate ± 100 nM LY overnight. Subsequently, oxygen consumption ratio was measured using Agilent Seahorse XF analyzer (left panel) and the mitochondrial function parameters were calculated from the kinetic data (right panel). (**C**) Representative images of hLSECs stained with MitoTracker (green) and LipiTOX (red). hLSECs were incubated overnight with 500 μM Palmitate ± 20 nM GSK3 inhibitor LY, followed by staining and examination under confocal microscopy. Scale bar: 5 μm. (**D**) Parameters for mitochondrial circularity and complexity. Created in BioRender. (**E**–**H**) Quantification of mitochondrial number (**E**), aspect ratio (**F**), perimeter (**G**), and form factor (**H**) in hLSECs. (**I**) Quantification of lipid droplet number per cell area in hLSECs. (**J**) Quantification of lipid droplet area per cell area in hLSECs. (**K**) Representative images of LSECs isolated from CDHFD-fed *Gsk3β*^ΔEnd^ and *Gsk3β*^fl/fl^ stained with MitoTracker (green) and LipiTOX (red). Scale bar: 5 μm. (**L**–**O**) Quantification of mitochondrial number (**L**), aspect ratio (**M**), perimeter (**N**), and form factor (**O**) in LSECs isolated from mice. (**P**) Quantification of lipid droplet number per cell area in LSECs from mice. (**Q**) Quantification of lipid droplet area per cell area in LSECs from mice. (**R**) Schematic diagram showing DRP1-induced mitochondrial fission. Created in BioRender. (**S**) Western blotting of pDRP1 (Ser616) and GAPDH from palmitate-treated hLSECs ± GSK3 inhibitor LY. Bar graphs represent the mean ± SEM; *P < 0.05; **P < 0.01; ***P < 0.001; ****P < 0.0001 (2-way ANOVA with Bonferroni’s multiple comparison for **A** and **B**; 1-way ANOVA with Bonferroni’s multiple comparison for other graphs).

To examine mitochondrial biogenesis and dynamics in hLSECs under lipotoxic conditions, we examined mRNA expression of genes related to mitochondrial function and biogenesis, optic atrophy 1 (*OPA1*) (47), nuclear respiratory factor 1 (*NRF1*) (48), transcription factor A, mitochondrial (*TFAM*) (49), and mitofusin-2 (*MFN2*) (50) using our in vitro model of lipotoxicity. We identified decreased expression of these genes in hLSECs treated with palmitate and restoration with LY treatment (Figure 6A). These genes are known to be regulated by the AMPK pathway (45, 46). To assess the effect of lipotoxicity on mitochondrial respiration in hLSECs, we performed Seahorse XF mitochondrial stress tests. Palmitate markedly suppressed basal oxygen consumption rate (OCR) and OCR elevation after carbonyl cyanide-4 (trifluoromethoxy) phenylhydrazone (FCCP) treatment, leading to reduced basal respiration, ATP production, maximal respiration, and spare respiratory capacity, all of which were restored by GSK3β inhibition with LY (Figure 6B).

In hepatocytes from patients with MASH, mitochondrial morphology shifts from tubular to spherical, correlating with reduced complete fatty acid oxidation, increased incomplete oxidation, and impaired oxidative phosphorylation (51). Hence, we confirmed that mitochondria in LSEC display similar spherical morphology and reduced density under palmitate-induced lipotoxic stress (Figure 6C). Interestingly, treatment of human LSECs with oleate or linoleate induced greater lipid droplets accumulation than palmitate, whereas palmitate caused more pronounced mitochondrial morphological alterations compared with the other fatty acids (Supplemental Figure 9A). GSK3 inhibition with LY attenuated the mitochondrial morphological alterations induced by palmitate (Figure 6C). To provide an objective assessment, we calculated the mitochondrial number, as well as aspect ratio, perimeter, and form factor, which reflect the complexity of mitochondrial morphology, as previously reported (Figure 6D) (52). We confirmed a decrease in mitochondrial number (Figure 6E), aspect ratio (Figure 6F), perimeter (Figure 6G), and form factor (Figure 6H) in hLSECs under palmitate treatment, which were restored by LY. Furthermore, lipid droplet accumulation was significantly decreased by LY treatment (Figure 6, I and J). Comparable improvement of the mitochondrial morphology and lipid droplet accumulation was confirmed in LSECs isolated from the CDHFD-fed *Gsk3β*^ΔEnd^ mice compared with CDHFD-fed *Gsk3β*^fl/fl^ mice (Figure 6, K–Q). Furthermore, pharmacological GSK3 inhibition restored mitochondrial reactive oxygen species (ROS) generation in hLSECs treated with palmitate when examined by MitoSOX staining (Supplemental Figure 9B).

Dynamin-related protein 1 (DRP1) is a GTPase that mediates mitochondrial constriction at fission sites, thereby regulating mitochondrial morphology. Upon phosphorylation at Ser616, DRP1 translocates from the cytosol to the mitochondrial outer membrane to execute fission (53) (Figure 6R). Interestingly, palmitate enhanced pDRP1 (Ser616) protein expression in hLSECs, which was suppressed with pharmacological GSK3 inhibition (Figure 6S). These findings suggest that, under lipotoxic conditions, GSK3 activation drives mitochondrial fragmentation in LSECs through DRP1 activation.

### LSEC mitochondrial dysfunction drives inflammation in MASH via NF-κB activation.

Since pathway analysis of in vitro transcriptomic profiling identified NF-κB pathway enrichment (Figure 5B), we examined the effects of lipotoxicity and GSK3 inhibition on NF-κB activation in LSECs.

**Figure 7:**
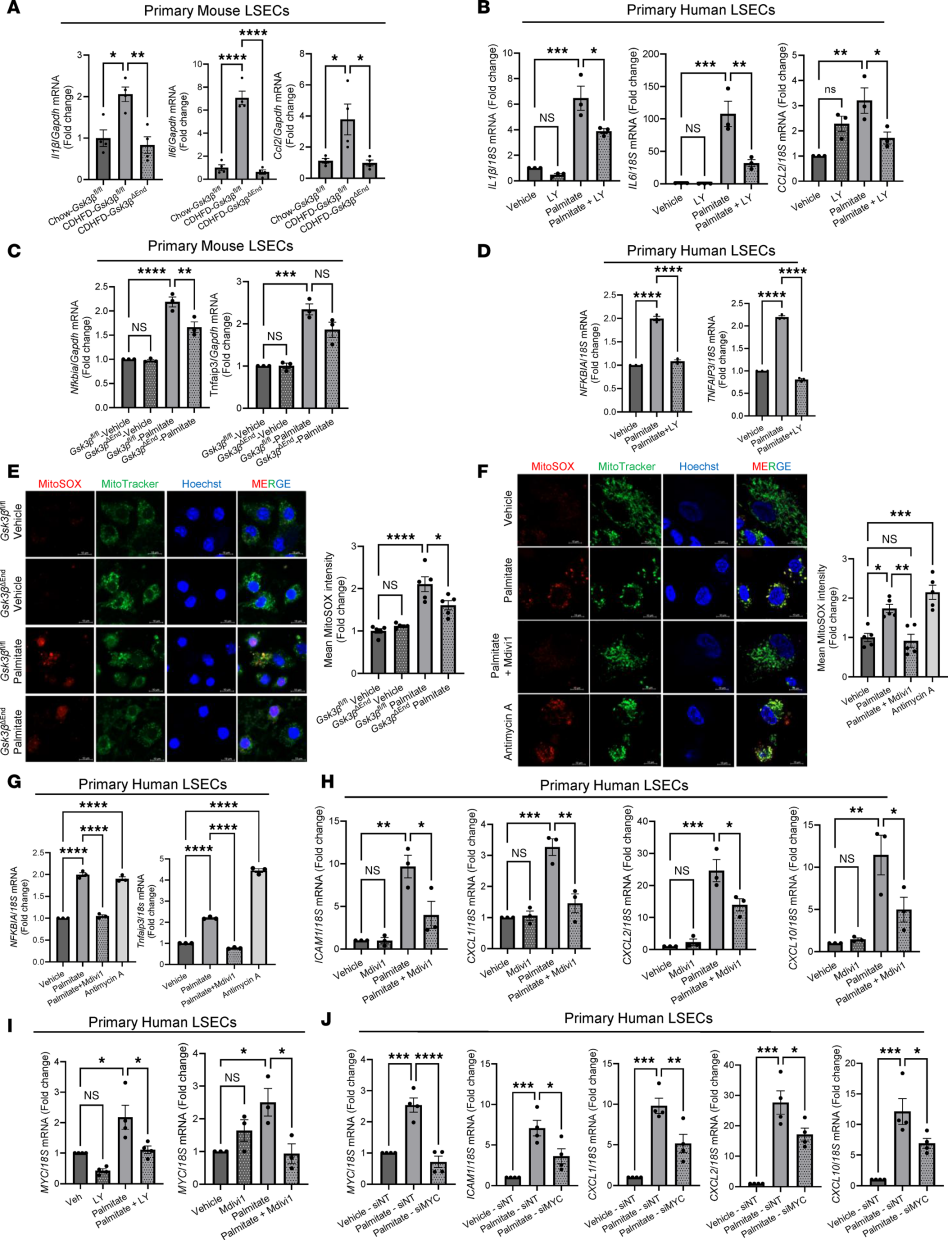
LSEC mitochondrial dysfunction drives inflammation in lipotoxicity and MASH via NF-κB and MYC activation. (**A**) mRNA expression of *Il1b*, *Il6*, and *Ccl2* in LSECs isolated from mice. (**B**) mRNA expression of *IL1B*, *IL6*, and *CCL2* in hLSECs treated with palmitate and GSK3 inhibitor LY. (**C**) mRNA expression of *Nfkbia* and *Tnfaip3* in primary mouse LSECs treated with 500 μM palmitate for 2 hours. (**D**) mRNA expression of *NFKBIA* and *TNFAIP3* in hLSECs treated with 800 μM palmitate and the GSK3 inhibitor LY for 2 hours. (**E** and **F**) Representative images of primary mouse LSECs (**E**) and hLSECs (**F**) treated with palmitate, Mdivi1, and antimycin A, stained with MitoTracker (green) and MitoSOX (red). Scale bar: 5 μm. The MitoSOX quantification is shown in the right panel. (**G**) mRNA expression of *NFKBIA* and *TNFAIP3* in hLSECs treated with 800 μM palmitate, Mdivi1, and antimycin A for 2 hours. (**H**) mRNA expression of *ICAM1*, *CXCL1*, *CXCL2*, and *CXCL10* in hLSECs treated with palmitate and Mdivi1. (**I**) mRNA expression of *MYC* in hLSECs treated with palmitate, GSK3 inhibitor LY and Mdivi. (**J**) mRNA expression of *MYC*, *ICAM1*, *CXCL1*, *CXCL2*, and *CXCL10* in hLSECs following *MYC* silencing by siRNA. Bar graphs represent the mean ± SEM; *P < 0.05; **P < 0.01; ***P < 0.001; ****P < 0.0001 (1-way ANOVA with Bonferroni’s multiple comparison).

In LSECs from CDHFD-fed mice, NF-κB target genes (*Il1b*, *Il6*, and *Ccl2*) were markedly upregulated, whereas *Gsk3β* deletion significantly suppressed their expression (Figure 7A). Consistently, palmitate increased the expression of these genes in hLSECs, which was attenuated with GSK3 pharmacologic inhibition (Figure 7B). Furthermore, palmitate increased the expression of these genes in primary mouse LSECs, whereas genetic *Gsk3β* deletion suppressed this induction (Supplemental Figure 9C). *Nfkbia* and *Tnfaip3*, which are upregulated by NF-κB activation, were induced by palmitate stimulation in both mouse primary LSECs (Figure 7C) and hLSECs (Figure 7D), and genetic (Figure 7C) or pharmacologic (Figure 7D) inhibition of GSK3 reduced their expression. Collectively, these data indicate that palmitate-induced lipotoxicity activates the NF-κB pathway in LSECs and that GSK3β inhibition suppresses this activation.

NF-κB is known to be activated by mitochondrial ROS, which induce PINK1/Parkin-dependent ubiquitination of damaged mitochondria, leading to the recruitment of the NF-κB effector NEMO and subsequent activation of the IKK complex that drives inflammatory NF-κB signaling (54). We confirmed that palmitate increased mitochondrial ROS in primary mouse LSECs, while *Gsk3β* deletion reduced it, as shown by MitoSOX staining (Figure 7E). To determine whether mitochondrial ROS activates the NF-κB pathway in LSECs, we treated hLSECs with Mdivi-1, a mitochondrial division inhibitor that reduces mitochondrial ROS (55). Confocal microscopy confirmed that Mdivi-1 maintained mitochondrial morphology under palmitate treatment and suppressed palmitate-induced mitochondrial ROS (Figure 7F). Mdivi-1 also decreased the expression of *Nfkbia* and *Tnfaip3* (Figure 7G), suggesting that mitochondrial ROS contribute to NF-κB activation. Conversely, treatment with antimycin A, an inhibitor of mitochondrial electron transport complex III and a known mitochondrial ROS inducer (56), increased mitochondrial ROS (Figure 7F), and upregulated *Nfkbia* and *Tnfaip3* expression (Figure 7G). These findings support a critical role for mitochondrial ROS in NF-κB activation in LSECs. Mdivi-1 further suppressed palmitate-induced upregulation of *ICAM1*, *CXCL1*, *CXCL2*, and *CXCL10* (Figure 7H), which are direct NF-κB targets (57, 58). These results support the concept that the mitochondrial ROS/NF-κB axis contributes to the proinflammatory phenotype of LSECs. Furthermore, we identified downregulation of *Myc* in CDHFD-fed *Gsk3β*^ΔEnd^ mice (Figure 4, A and C), and cMYC is partially regulated by NF-κB activity (59). Likewise*,* in human LSEC, *MYC* expression was increased by palmitate and reduced by GSK3 inhibition or Mdivi-1 treatment (Figure 7I). Furthermore, pharmacological inhibition of cMYC (MYCi) (Supplemental Figure 9D) and MYC silencing (siMYC) (Figure 7J) decreased *ICAM1* and *CXCL* family expressions.

Together, these findings suggest that cMYC acts as a downstream effector of the mitochondrial ROS/NF-κB signaling cascade and plays a key role in regulating the proinflammatory phenotype in lipotoxic endotheliopathy.

### Endothelial cell–specific Gsk3β deletion attenuates liver fibrosis in CDHFD-fed mice.

**Figure 8:**
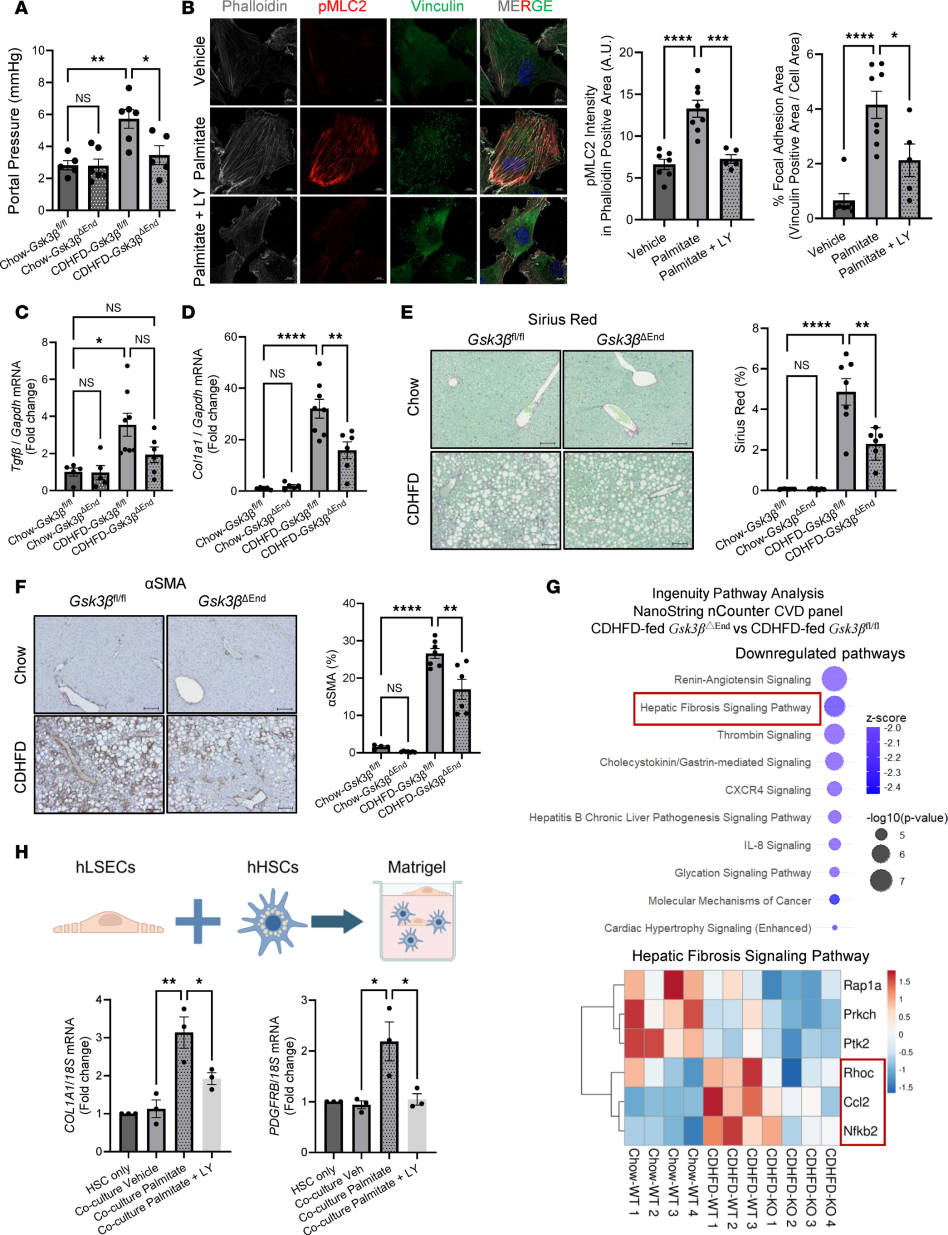
Endothelial cell–specific *Gsk3β* deletion attenuates liver fibrosis in murine MASH. (**A**) Portal pressure (mmHg) of CDHFD-fed mice was measured at the endpoint. (**B**) Immunofluorescence showing that palmitate-induced (400 μM) MLC2 phosphorylation and actin polymerization connecting focal adhesions in LSEC. (**C** and **D**) Whole liver mRNA expression of *Tgfβ* (**C**) and *Col1a1* (**D**). (**E** and **F**) Representative images showing Sirius red staining (**E**, left panel) and αSMA (**F**, left panel). Positive areas were quantified in 5 random 10× microscopic fields and averaged for each animal. (right panel). Scale bar: 100 μm. (**G**) Bubble chart showing the top 10 downregulated pathways in LSECs isolated from CDHFD-fed *Gsk3β*^ΔEnd^ versus CDHFD-fed *Gsk3β*^fl/fl^ mice (upper panel). Differential gene expression data obtained based on Nanostring CVD panel was analyzed and Ingenuity Pathway Analysis (Lower panel). The analysis included downregulated genes with a *P* value of less than 0.05. The color of the bubbles represents the *z* score, while the bubble size reflects the *P* value. (**H**) hLSECs and hHSCs were cocultured using the 3D coculture system with Matrigel. hLSECs treated with vehicle or palmitate 800 μM overnight then cocultured with hHSCs for 3 days in LSEC growth medium. HSCs activation was examined by mRNA expression of *COL1a1* and *PDGFRB*. Created in BioRender. Bar graphs represent the mean ± SEM; *P < 0.05; ***P < 0.001; ****P < 0.0001 (1-way ANOVA with Bonferroni’s multiple comparison).

To assess the effect of selective *Gsk3β* deletion in endothelial cell on hepatic fibrosis in MASH mice, we measured portal pressure and identified reduced portal pressure, suggesting attenuated vasoreactivity in addition to liver fibrosis in the CDHFD-fed *Gsk3β*^ΔEnd^ mice (Figure 8A). We identified a mechanical pathway linking LSEC stress responses to the increased vasoreactivity in MASH. Under palmitate-induced lipotoxicity, LSECs show a marked increase in actin stress fiber formation as assessed by phosphorylation of myosin light chain (pMLC) indicating enhanced actomyosin contractility, which was reduced by GSK3 inhibition (Figure 8B). Because endothelial contractility contributes to increased sinusoidal stiffness and elevated portal pressure, these findings support a model in which GSK3β-dependent cytoskeletal rearrangement in LSECs may exacerbate portal hypertension.

Consistent with the decrease in TGF-β^+^ monocyte-derived DCs/macrophages in the *Gsk3β*^ΔEnd^ mice compared with *Gsk3β*^fl/fl^ mice on CDHFD diet (Figure 3, E and F), hepatic *Tgfβ* mRNA expression showed a decreasing trend in the CDHFD-fed *Gsk3β*^ΔEnd^ mice compared with *Gsk3β*^fl/fl^ mice (Figure 8C). Likewise, Collagen, type I, α 1 chain (*Col1a1*) mRNA expression was reduced in *Gsk3β*^ΔEnd^ mice on the MASH inducing diet (Figure 8D). Furthermore, we identified significant reduction of liver fibrosis when assessed by Sirius red staining (Figure 8E) and αSMA IHC (Figure 8F) in *Gsk3β*^ΔEnd^ mice compared with *Gsk3β*^fl/fl^ mice on the CDHFD diet. These data were replicated in a different MASH mouse model induced by the MCD, confirming reduced Sirius red staining (Supplemental Figure 10A) and *Col1a1* expression in *Gsk3β*^ΔEnd^ mice compared with *Gsk3β*^fl/fl^ mice (Supplemental Figure 10B).

IPA of transcriptomic data from primary mouse LSECs, using the Nanostring nCounter CVD Pathophysiology Panel, identified significant downregulation of Hepatic Fibrosis Signaling Pathway in CDHFD-fed *Gsk3β*^ΔEnd^ mice compared with CDHFD-fed *Gsk3β*^fl/fl^ mice (Figure 8G). In the LSECs of CDHFD-fed *Gsk3β***^ΔEnd^** mice, the proinflammatory gene expressions, including NF-κB downstream *Ccl2* and NF-κB subunit 2 (*Nfkb2*), were also reduced compared with the *Gsk3β*^fl/fl^ mice (Figure 8G), supporting the concept of mitochondrial ROS/NF-κB axis in LSECs. Furthermore, Ras homolog family member C (RhoC), a regulator of actomyosin contractility (60), was upregulated in *Gsk3β^fl/fl^* mice fed a MASH-inducing diet and attenuated in *Gsk3β*^ΔEnd^ mice, consistent with the observed pMLC upregulation and stress fiber formation in LSECs.

To assess direct LSEC-HSC communication, we employed a primary human LSEC-HSC 3D coculture system. We observed that lipotoxic LSECs promote HSC activation, as shown by upregulation of *COL1A1* and *PDGFRB1* (Figure 8H). The observed effect was diminished when primary hLSECs were pretreated with a pharmacological GSK3 inhibitor prior to coculture. This finding indicates that endothelial GSK3β plays a direct role in mediating profibrogenic signaling to stellate cells, in addition to indirectly modulating profibrogenic responses via attenuating liver inflammation.

In summary, endothelial GSK3β enhances portal hypertension and liver fibrosis in MASH by modulating LSEC cytoskeletal rearrangement, inflammatory signaling, and direct profibrogenic communication with HSCs.

## Discussion

The current study provides key insights into the LSEC-specific role of GSK3β during the pathogenesis of MASH. We show that: (a) endothelial cell–specific *Gsk3β* deletion attenuates the proinflammatory phenotype of LSECs, reducing hepatic infiltration of pathogenic myeloid cells and limiting DC maturation and activation, thereby mitigating liver injury, inflammation, and fibrosis in diet-induced MASH mice; (b) endothelial cell–specific *Gsk3β* deletion suppresses chemokine and cytokine, as well as lipid and atherosclerosis signaling pathways in murine MASH LSECs; (c) GSK3 inhibition rescues lipotoxicity-induced alteration in mitochondrial respiration and morphology by modulating AMPK and DRP1 phosphorylation; (d) mitochondrial dysfunction induces a proinflammatory phenotype in LSECs under lipotoxic stress via NF-κB and cMYC activation; and (e) lipotoxic LSECs directly drive HSC activation, which is suppressed by GSK3 inhibition. Importantly, we confirmed the clinical translation of these findings in human MASLD by showing increased DC accumulation with MASLD progression and the suppression of LSEC endotheliopathy by GSK3 inhibition in human PCLS. While our previous studies showed the therapeutic efficacy of pharmacological GSK3 inhibition (19) and the hepatocyte-specific role of GSK3β in driving ferroptosis in MASH (61), the present study shows for the first time to our knowledge that LSEC GSK3β is a pivotal driver of MASH modulating the hepatic inflammation through mitochondrial ROS/NF-κB signaling axis. This axis promotes the maturation of DCs and the recruitment of proinflammatory myeloid cells, thereby enhancing the proinflammatory responses in MASH. These findings highlight LSEC GSK3β as a central driver of endotheliopathy and sterile inflammation in MASH.

Our unbiased transcriptomic analysis of toxic lipid-treated hLSECs and LSECs isolated from CDHFD-fed mice identified significant alterations not only in pathways related to the LSEC proinflammatory phenotype, namely cytokine and chemokine-related signaling, but also in the lipid and atherosclerosis and AMPK pathway. Mitochondria is the cell metabolic hub that integrates multiple metabolic pathways preserving cellular redox balance and limiting lipid peroxidation (62, 63). AMPK regulates the key genes controlling mitochondrial biogenesis and dynamics (45). In MASH, mitochondrial functions including fatty acid oxidation, oxidative phosphorylation, mitochondrial DNA homeostasis, and mitochondrial dynamics are impaired (64). Herein, we show that GSK3β inhibition in LSEC under lipotoxic stress improved mitochondrial morphology and respiration likely via increased pAMPK (Thr172), and it enhanced expression of mitochondrial dynamic and biogenesis (65, 66). Furthermore, our study confirmed that pharmacological GSK3β inhibition suppressed palmitate-enhanced DRP1 phosphorylation at Ser616, the key player in mitochondrial fission. DRP1 is a GTPase that regulates mitochondrial morphology; when phosphorylated at Ser616, it translocates from the cytosol to the outer membrane to drive mitochondrial fission at division sites (53). Taken together, our findings support that GSK3 inhibition rescues lipotoxicity-induced mitochondrial dysfunction via regulating AMPK and DRP1 phosphorylation, resulting in reduced expression of adhesion molecules and chemokines and thereby attenuating lipotoxic endotheliopathy.

Our flow cytometry and CyTOF data indicate that endothelial cell–specific *Gsk3β* deletion in mice with diet-induced MASH reduced intrahepatic monocyte-derived mature DCs and inflammatory macrophages. Although DCs constitute less than 1% of the total nonparenchymal liver cell population, they promote the immune tolerance properties of the liver through the production of antiinflammatory cytokines and induction of regulatory T cells (67, 68). With unresolved liver injury and persistent insults, liver-resident DCs undergo maturation and acquire the capacity to produce proinflammatory cytokines that activate other immune cells (69) and HSCs (70). However, the role of lipotoxic endotheliopathy in DC maturation in MASH has been unclear. Chemokines such as CCL2-5 and CXCL9/10 are strongly associated with the differentiation and activation of DCs (71). Transcriptomic profiling identified *Cxcl10* upregulation in CDHFD-fed *Gsk3β*^fl/fl^ mice, with significant reduction in *Gsk3β*^ΔEnd^ mice on the same diet. Under homeostatic conditions, CXCL10 is minimally expressed in LSECs (Liver Cell Atlas), with significant upregulation in response to stressors such as lipopolysaccharide (LPS) (72) or lipid (73). Our coculture experiment further shows that the direct interaction between lipotoxic LSECs and DCs was essential for DC maturation, which was suppressed when LSECs were pretreated with a GSK3 inhibitor. Collectively, these findings indicate that GSK3β plays a pivotal role in the interaction between LSECs and DCs through LSEC-derived secretory factors, including chemokines, and direct contact via adhesion molecules such as LSEC ICAM1 and DC Integrin αXβ2, modulating immune activation in MASH.

In addition to DCs and monocyte-derived macrophages, we observed that LAMs was enriched in MASH mouse livers and showed a downward trend in *Gsk3β*^ΔEnd^ mice. TREM2^+^ macrophages are known to maintain the fibrotic niche during MASH progression, and LSECs are vital for the recruitment and maintenance of these cells via adhesion molecules and secretory factors (34). Furthermore, the expression of vascular endothelial adhesion molecules recruits SPP1^+^ macrophages and promotes fibrosis (74). Our study shows that Gsk3β-driven mitochondrial ROS in LSECs upregulates ICAM1 and various chemokines. Thus, the reduction of adhesion molecules and chemokine expression by LSEC through *GSK3β* deletion likely impairs the recruitment of LAMs, thereby limiting liver fibrosis.

Our data indicate that the lipid and atherosclerosis pathway was significantly activated under palmitate treatment via a GSK3-dependent mechanism. Furthermore, GSK3 inhibition reduced lipid droplets in LSECs and lowered portal pressure. Impaired lipid metabolism and lipid droplet accumulation in vascular endothelial cells have been implicated in systemic hypertension, endothelial dysfunction, and activation of proinflammatory signaling cascades (42, 43). Improved mitochondrial function and β-oxidation by GSK3 inhibition may contribute to the enhancement of lipid metabolism in LSECs (75).

While HSC activation in liver fibrosis is primarily attributed to the inflammatory response including monocyte-derived macrophages (8) and DCs (70), emerging evidence highlights the pivotal roles of direct signaling from endothelial cells (8). Our study shows that endothelial *GSK3β* not only modulates the inflammatory microenvironment but also directly promotes HSC activation. This dual mechanism is supported by recent findings that vascular niche dysfunction and altered endothelial vasoreactivity act as drivers of fibrogenesis (76). Notably, a recent study reported that the upregulation of ROCK2, a downstream kinase of the Rho GTPase pathway, in LSECs drives profibrogenic signaling, and its selective inhibition attenuates liver fibrosis (77). Our data show that GSK3β modulates stress fiber formation, likely via the RhoC-MLC contractility axis (78) as supported by NanoString on LSECs isolated from CDHFD-fed mice and immunostaining on hLSECs. Collectively, in addition to promoting fibrosis by modulating the inflammatory microenvironment, endothelial GSK3β drives liver fibrosis and portal hypertension through a coordinated mechanism involving enhanced vasoreactivity and direct activation of HSCs.

To further advance the findings of this study, several future investigations are warranted. First, the observed enrichment of B cell populations with low costimulatory molecule expression in *Gsk3β*^ΔEnd^ mice suggests a potentially more tolerogenic intrahepatic environment. However, the role of endothelial GSK3β in B cell recruitment and maturation in MASH remains unexplored and is beyond the scope of the current manuscript. Second, emerging evidence indicates that the intercellular transfer of mitochondria can modulate immune responses (79). Given that GSK3β is a central driver of mitochondrial dysfunction in LSECs, and the direct communication between LSECs and DCs is essential for DC activation, investigating whether the damaged mitochondrial transfer from LSECs contributes to the pathogenic DCs is a future direction of our research program.

Collectively, our study indicates that GSK3β in LSECs plays a critical role in driving mitochondrial dysfunction and immune activation in MASH. GSK3β inhibition in LSEC under lipotoxic stress mitigates mitochondrial dysfunction, the proinflammatory phenotype, and pathogenic immune cells recruitment, thereby alleviating liver injury, inflammation, and fibrosis. Hence, our study supports the role of GSK3β as a potential therapeutic target in human MASH.

## Methods

### Sex as a biological variable.

Our study examined male mice because male animals exhibited less variability in phenotype. It is unknown whether the findings are relevant for female mice.

### Animals and diet-induced murine mash models.

Male *Gsk3β*^fl/fl^ mice were crossed with *Cdh5(PAC)-CreERT2* mice. At 6 weeks of age, offspring were injected with tamoxifen (160 mg/kg, 7 days) to generate *Gsk3β*^ΔEnd^ mice. At 8 weeks of age, mice were fed a choline-deficient high-fat diet (CDHFD; Research Diets) for 5 weeks to induce MASH. Alternatively, C57BL/6J mice were fed a methionine-choline-deficient (MCD) diet for 3 weeks. Detailed information and diet rationales are provided in the Supplemental Methods.

### Cells.

Primary hLSECs were purchased from ScienCell Research Laboratories. Primary mouse LSECs were isolated using a method based on liver collagenase perfusion and immunomagnetic selection as previously described (15, 80). Detailed information are provided in the Supplementary Methods. All the cell cultures were maintained at 37°C in a humidified atmosphere of 5% CO_2_. Upon reaching 70%–80% confluence, cells were treated with either 800 μM palmitate to induce lipotoxicity ± 20 nM of the GSK3 inhibitor LY for 8 hours, or vehicles (isopropanol or DMSO).

### PCLS.

Human liver tissues from consented patients undergoing resection were processed into 250 μm-thick PCLS as previously described (81, 82). Following an initial 24-hour recovery period, PCLS were cultured for 96 hours in either control media or a MASLD-inducing medium (GFIPO) (23), with or without 20 nM of the GSK3 inhibitor LY. Samples were then harvested for RNA and protein isolation. Detailed procedures for tissue processing and media composition are provided in the Supplemental Methods.

### Coculture models.

For 2D cocultures, human DCs were cultured directly with palmitate/LY-pretreated hLSECs, or in their conditioned medium, followed by flow cytometry analysis. For 3D cocultures, hLSECs and human HSCs (hHSCs) were coseeded on Matrigel Matrix (Corning Inc.) and treated with palmitate ± LY for 3 days before qPCR analysis. Detailed procedures and antibody information are in the Supplemental Methods.

### Statistics.

Data are presented as mean ± SEM. Statistical significance (*P < 0.05, **P < 0.01, ***P < 0.001, ****P < 0.0001) was determined using an unpaired 2-tailed Student’s *t* test, Mann-Whitney U test, or 1-way ANOVA with Bonferroni’s post hoc test (multiple groups) via GraphPad Prism 9.2.0. A *P* value less than 0.05 was considered significant. Schematic diagrams were created using BioRender (https://BioRender.com). Specific citations for each figure are as follows: Graphical Abstract (https://BioRender.com/xxu9z7u); Figure 1A (https://BioRender.com/8faxae9); Figure 1D (https://BioRender.com/rgysqdx); Figure 4E (https://BioRender.com/5c74qrm); Figure 5H (https://BioRender.com/zfvyxxm); Figure 6D (https://BioRender.com/kooqcnl); Figure 6R (https://BioRender.com/hwmnm8v); Figure 8H (https://BioRender.com/q298b12); and Supplemental Figure 7F (https://BioRender.com/ynnwkwg).

### Study approval.

All animal studies were approved and performed in accordance with the IACUC at Mayo Clinic (A00003506-18-R24). The investigation involving clinical samples was approved by the Mayo Clinic IRB (approval no. 22-000320). Written informed consent was obtained from all participants prior to their inclusion in the study.

### Data availability.

Data associated with this study are available in the main text or in the Supporting Data Values file. RNA-seq data on hLSECs and isolated LSECs are in GEO database (accession no. GSE 309912 and GSE 164006, respectively).

## Author contributions

SHI, AS, QG, KW, PH, and D Povero were responsible for the experimental study designs. AS, QG, AH, GH, NS, XD, UY, C Meroueh, and KDP conducted experiments and data collection. C Miciano and TK provided human single nuclear RNA seq. ZF, PPS, D Pereyra, and EK prepared and provided human-derived PCLS. EK, PH, and D Povero provided research advice and discussions. AS, QG, C Meroueh, KDP, and SHI performed the formal analysis. AS wrote the original draft. SHI formulated the study concept, edited the manuscript, and was responsible for funding acquisition. All authors reviewed and approved the final manuscript.

## Conflict of interest

The authors have declared that no conflict of interest exists.

## Funding support

This work is the result of NIH funding, in whole or in part, and is subject to the NIH Public Access Policy. Through acceptance of this federal funding, the NIH has been given a right to make the work publicly available in PubMed Central.

- NIH (R01DK122948 and P30DK084567 to SHI)
- Postdoctoral Fellowship from the American Liver Foundation, the Study Abroad Grant from the Mochida Memorial Foundation for Medical and Pharmaceutical Research, the Travel Grant from the Japanese Society of Gastroenterology, the Fellowship from the Uehara Memorial Foundation, and the Konno Overseas Scholarship from Tohoku University (AS)
- National Research Council of Thailand (NRCT) (Contact No. N41A661125) and the Second Century Fund (C2F), Chulalongkorn University (NS).
- NIH R01DK111866, R56DK088837, DK099205, AA028550, DK101737, AA011999, DK120515, AA029019, DK091183, P42ES010337, R44DK115242 to TK.
- Stem Cell Fitness and Space Medicine Center at Sanford Stem Cell Institute (UCSD).

## Supplementary Material

Supplemental data

Unedited blot and gel images

Supporting data values

## Version 1 05/05/2026

In-Press Preview

## Version 2 06/22/2026

Electronic publication
