# Supplementary material for: Endothelial cell glycogen synthase kinase 3β promotes lipotoxic endotheliopathy and liver inflammation in MASH
Source: JCI Insight. 2026 May 5;11(12):e202552. doi: 10.1172/jci.insight.202552 (PMC13313496; doi:10.1172/jci.insight.202552)
Supplement: Supplemental data [file jciinsight-11-202552-s120.pdf]

Endothelial Cell Glycogen Synthase Kinase 3β Promotes Lipotoxic Endotheliopathy and Liver Inflammation in MASH

Akitoshi Sano[1,2], Qianqian Guo[1], Khaled Warasnhe[1], Chady Meroueh[3], Nantawat Satthawiwat[1,4], Asma Hamdi[5], Ghefar Hmaydoosh[1], Xin Dai[1,6], Usman Yaqoob[1], Kevin D. Pavelko[7,8], Charlene Miciano[9,10], Tatiana Kisseleva[11], Zeba Firdaus[12], Patrick P. Starlinger[13, 14], David Pereyra[13, 14, 15], Enis Kostallari[1], Petra Hirsova[1], Davide Povero[1], Samar H. Ibrahim[1,5]

[1]Division of Gastroenterology & Hepatology, Mayo Clinic, Rochester, Minnesota, USA; [2]Division of Gastroenterology, Tohoku University Graduate School of Medicine, Sendai, Japan; [3]Department of Laboratory Medicine and Pathology, Mayo Clinic, Rochester, Minnesota, USA; [4]Department of Biochemistry, Medical Biochemistry Program, Faculty of Medicine, Chulalongkorn University, Bangkok, Thailand; [5]Division of Pediatric Gastroenterology & Hepatology, Mayo Clinic, Rochester, Minnesota, USA; [6]Department of Gastroenterology and Hepatology, Tianjin Medical University General Hospital, Tianjin, China; [7]Immune Monitoring Core, Mayo Clinic, Rochester, Minnesota, USA; [8]Department of Immunology, Mayo Clinic, Rochester, Minnesota, USA; [9]Department of Cellular and Molecular Medicine, University of California San Diego School of Medicine, La Jolla, California, USA; [10]Center for Epigenomics, University of California San Diego School of Medicine, La Jolla, California, USA; [11]Department of Surgery, University of California San Diego, La Jolla, California, USA; [12]Department of Biochemistry and Molecular Biology, Mayo Clinic, Rochester, Minnesota, USA; [13]Division of Hepatobiliary and Pancreas Surgery, Department of Surgery, Mayo Clinic, Rochester, Minnesota, USA; [14]Centre of Physiology and Pharmacology, Medical University of Vienna, Vienna, Austria; [15]Department of General Surgery, Medical University of Vienna, Vienna, Austria

Corresponding Author: Samar H. Ibrahim, MBChB
Professor of Pediatrics and Physiology
Division of Pediatric Gastroenterology
Department of Pediatric and Adolescent Medicine
Mayo Clinic
200 First Street SW
Rochester, MN 55905
Phone: (507) 266-0114
FAX: (507) 284-0160
E-mail: ibrahim.samar@mayo.edu

1. Supplemental methods

Materials. Palmitate (P0500) was obtained from Sigma-Aldrich (St. Louis, MO). LY2090314 (S7063) was obtained from Selleck Chemicals, LLC (Houston, TX). Elraglusib (9-ING-41) was gifted by Acuate Therapeutics Inc. (Fort Worth, TX). MYCi975 was purchased from MedChemExpress (HY-129601, Monmouth Junction, NJ). Mdivi1 was purchased from MedChemExpress (HY-15886). Antimycin A was obtained from Abcam (ab141904, Cambridge, MA). The primary antibodies employed in the study include anti-α-SMA (ab124964) from Abcam, anti-GAPDH (MAB374) from Millipore Sigma, anti-GSK3β (D5C5Z) (#12456), anti-MPO (#AF3667), anti-F4/80 (#70076S), anti-phospho-AMPKα (Thr172) (#2535), anti-phospho-Acetyl-CoA Carboxylase (Ser79) (#3661), anti-phospho-DRP1 (Ser616) (#3455), anti-DRP1 (#8570) from Cell Signaling Technology (Danvers, MA), anti-ICAM1/CD54 (AF796) from R&D systems (Minneapolis, MN), phosphor-GSK3β (#44-604G) from Invitrogen (Waltham, MA). Anti CD31-AF-647 and Phalloidin-iFluor 647 reagent (ab176759) was purchased from Abcam. MHC-II-FITC (327005) and anti CD11c-AF568 (371502) were purchased from BioLegend (San Diego, CA). Eraglusib (9-ING-41) was provided as a gift by Dr. Andrew (Mazar Inc, Fort Worth, TX).

Animals and Diet-induced Murine MASH Models. Male C57Bl/6J mice (Jackson Laboratory, Bar Harbor, ME, USA) were crossed with *Cdh5(PAC)-CreER*^{T2} mice (1, 2). At 6 weeks of age, *Gsk3β*$^{fl/fl}$ *Cdh5(PAC)-CreER*^{T2} mice were injected intraperitoneally with 160 mg/kg of tamoxifen for 7 days to generate *Gsk3β*$^{\Delta End}$ mice. *Cdh5(PAC)-CreER*^{T2}-negative littermates received the same tamoxifen dose and served as control mice (*Gsk3β*$^{fl/fl}$). Knock-down efficacy was assessed by western blotting. The chow diet was a standard rodent diet (5053 PicoLab Rodent Diet 20; LabDiet, St. Louis, MO, USA) with tap water. At 8 weeks of age, mice were fed a CDHFD (A06071302, Research Diet), which consists of 60% fat, 0.1% methionine, and no added choline,

for 5 weeks to induce MASH. CDHFD-fed mice experienced minimal body weight loss compared to those fed with the traditional MCD diet. These mice effectively recapitulate the histopathological features of human MASH in terms of hepatic steatosis, elevated ALT levels, hepatic inflammation, and fibrosis (3, 4). Since Cre recombinase activity may be reduced overtime after tamoxifen administration (5), we fed mice CDHFD, which causes MASH phenotype within a relatively short feeding period when compared with the FFC diet model (6). Alternatively, to replicate findings, C57BL6J mice were fed a MCD (A02082002Bri, Research Diet) for three weeks. All mice in this study were cohoused at the Mayo Clinic Rochester animal facility with a 12-hour light-dark cycle. All experimental interventions were performed during the light cycle. The use of anesthetics in experimental mice was conducted according to the IACUC guidelines.

Assessment of metabolic profiles in mice. Total caloric intake was calculated based on the weight of food consumption. Blood glucose and plasma insulin levels were measured using Assure 4 (Arkray, Edina, MN) and Ultra-Sensitive Mouse Insulin enzyme-linked immunosorbent assay (ELISA) kits (Crystal Chem Inc., Downers Grove, IL), respectively. The homeostasis model assessment of insulin resistance (HOMA-IR) was calculated using the following formula: HOMA-IR = 26 × fasting insulin level (ng/mL) × fasting glucose level (mg/dL)/405 (7).

Measurement of liver triglyceride and alanine aminotransferase levels. Liver triglyceride (TG) levels were measured from liver specimens. Fifty milligrams of liver tissue were homogenized in a 5% NP-40 solution. Tissue homogenate was boiled twice and centrifuged at 20,000g for 2 minutes. The extracted triglycerides were diluted then quantified using the EnzyChrom Triglyceride Kit (BioAssay System, CA, USA) according to the manufacturer's instructions. The photometric absorbance readings were obtained at 570 nm using a Synergy H1 microplate reader

(BioTek). The VetScan2 system (Abaxis Veterinary Diagnostics, Union City, CA, USA) was used for blood biochemical analysis.

Histological, immunohistochemical, and digital imaging analyses. Hematoxylin and eosin (H&E) staining was performed on murine liver samples to visualize and assess histopathological changes. The non-alcoholic fatty liver disease (NAFLD) activity score (NAS), a semi-quantitative score to evaluate the severity of liver pathology in murine models that accounts for steatosis, ballooned hepatocytes, and lobular inflammation was used to assess the liver samples (8). Sirius red staining were performed as previously described (9). Sirius red-stained tissue was imaged using light microscope and the tissue area was determined using ImageJ software. Formalin-fixed paraffin-embedded mouse liver tissue sections were deparaffinized, hydrated, and stained with antibodies against F4/80 (1:500), α-SMA (1:1,000), ICAM1 (1:2000). The bound antibody was detected using a Vectastain ABC kit for goat (PK-6105 or PK-6101, Vector Laboratories, Burlingame, CA) and DAB substrate (Vector Laboratories) according to the manufacturer's instructions. Tissue sections were counterstained with hematoxylin. MPO staining was evaluated by immunofluorescence with an anti-goat alexa-568 secondary antibody (A-11057, Thermo Fisher Scientific). Liver injury was assessed by the number of apoptotic hepatocytes using the terminal deoxynucleotidyl transferase deoxyuridine triphosphate nick-end labeling (TUNEL) assay and an In Situ Cell Death Detection Kit (Roche) following the

manufacturer's instructions.

Portal pressure measurements. Portal pressure was measured using a digital blood pressure analyzer. After calibration of the analyzer, a 16-guage catheter attached to a pressure transducer was inserted into the portal vain. The average portal pressure (mmHg) was then recorded.

Quantitative reverse transcription PCR (qRT-PCR). Total RNA was isolated using the Zymo Research Direct-zol™ RNA MiniPrep Kit (Zymo Research, Irvine, CA, USA) following the manufacturer's protocol. For cDNA synthesis, 1 μg of total RNA was reverse transcribed using the LightCycler® 480 SYBR Green I Master (Roche Diagnostics, Indianapolis, IN, USA) according to the manufacturer's instructions. The resulting cDNA was diluted 1:5 with nuclease-free water and used as a template for qRT-PCR. Gene expression was quantified by real-time PCR using SYBR green fluorescence on a LightCycler 480 instrument (Roche Applied, IN, USA) (primers are listed in Supplementary Table 4). The target gene expression was calculated and normalized using the $^{\Delta\Delta}$Ct method.

Immunoblot analysis. Mouse liver tissues were homogenized in T-PER lysis buffer (78510, Roche) containing protease and phosphatase inhibitors (78442, Thermo Scientific). Cells were lysed using RIPA buffer (50 mM Tris-HCl, pH 7.4; 1% Nonidet P-40; 0.25% sodium deoxycholate; 150 mM NaCl; 1 mM EDTA with protease inhibitors). The homogenates were centrifuged at 15,000g for 15 minutes at 4°C to remove debris, and the supernatants were collected. Protein concentrations were quantified using the Bradford assay (Sigma-Aldrich). Equal amounts of protein were loaded onto sodium dodecyl sulfate (SDS)-sulfate-polyacrylamide gel electrophoresis (PAGE) gels and then transferred to nitrocellulose membranes (Bio-Rad, Hercules, CA, USA). Following transfer, membranes were blocked in 5% non-fat dry milk in Tris-buffered saline with 0.1% Tween-20 (TBST) for 1 hour at room temperature. Membranes were then incubated overnight at 4°C with primary antibody of interest diluted in 5% BSA or 5% non-fat dry milk in TBST. All primary antibodies were used at a dilution of 1:1,000 unless otherwise recommended by the manufacturer. After primary antibody incubation, membranes were washed three times with TBST and incubated with Horseradish peroxidase-conjugated (HRP)-conjugated

secondary antibodies against rabbit (Alpha Diagnostic International, San Antonio, TX, USA) or mouse (Southern Biotech, Birmingham, AL, USA) (1:5000 dilution) in 5% non-fat dry milk in TBST for 1 hour at room temperature. Following secondary antibody incubation, membranes were washed three times with TBST. Protein bands were visualized using an enhanced chemiluminescence (ECL) detection reagents (GE Healthcare, Chicago, IL, USA). GAPDH protein levels were used as loading controls. Band intensity was quantified using ImageJ software and normalized to GAPDH levels.

Immunocytochemistry and confocal microscopy

hLSECs were fixed with 4% PFA for 20 mins at room temperature and permeabilized using 0.01% TritonX-100 for 5 min, then blocked with 5% BSA for one hour. Cells were incubated with primary antibodies overnight at 4°C. Cells were labeled using Alexa Fluor 596-conjugated donkey anti-rabbit IgG (1:2,000), Alexa Fluor 488-conjugated donkey anti-rabbit IgG (1:2,000), or Alexa Fluor 596-conjugated chicken anti-mouse IgG (1:2,000), and observed under confocal microscopy (LSM 980, Zeiss, Jena, Germany). 4', 6-diamidino-2-phenylindole (DAPI) was used for the nuclear counterstain. ZEN 2.3 lite software (ZEISS) was used for acquiring images. To quantify pMLC2, a stress fiber ROI was generated from the phalloidin channel via background subtraction and thresholding. A pMLC2 intensity was then calculated as the ratio of mean pMLC2 intensity on the stress fibers. For focal adhesion analysis, vinculin images were subjected to background subtraction and restricted to the whole-cell ROI. Focal adhesions were quantified via particle analysis (size $\geq$0.2 μm^2) in Image J. Focal adhesion area fraction (%) were subsequently determined based on the total cell area.

Live cell imaging. Mitochondrial staining was performed using MitoTracker™ Green (Thermo Fisher Scientific, #CST9074, 1:3000) to label active mitochondria. Mitochondrial ROS was

stained by MitoSOX™ (Thermo Fisher, M36008). hLSECs or isolated mouse LSECs were washed with phosphate-buffered saline (PBS) and incubated with 100 nM MitoTracker, 5 μM MitoSOX, and Hochest (33342, 1:1000) in PBS for 30 minutes at 37°C. After incubation, the cells were washed with PBS three times to remove excess dye. Lipid droplets were stained using LipidTox™ Neutral Lipid Stain (Thermo Fisher Scientific, #34477, 1:3000). Images were captured using a C-Apochromat 63_objective/1.20 W korrM27 of a fluorescent confocal microscope (LSM780 AxioObserver, Carl Zeiss) and were processed using the Zen Lite software (Carl Zeiss). Mitochondria and lipid droplets in LSECs were quantified by FIJI software (10) and the plugin Mitochondria Analyzer (11). Data were expressed as mean values per cell. MitoSOX fluorescence intensity was quantified using FIJI software following a standardized image analysis workflow. Confocal image files (.czi) were opened in FIJI as hyperstacks and subsequently split into individual fluorescence channels corresponding to MitoTracker (green), MitoSOX (red), and Hoechst (blue). Mitochondrial regions of interest (ROIs) were identified from the MitoTracker (green) channel using the Mitochondrial Analyzer plugin with a two-dimensional thresholding approach. Next, the mitochondrial ROIs were overlaid onto the MitoSOX (red) channel to quantify superoxide signal intensity specifically within mitochondrial regions. Mean gray value was measured for each ROI. Data were expressed as mean fluorescence intensity per mitochondrial ROI.

LSEC isolation from mouse livers. The liver was digested with collagenase infused via portal vein and obtained cell suspensions were centrifuged at 50 g for 2 minutes to remove hepatocytes. The supernatant which includes non-parenchymal cells was subjected to LSEC isolation using CD146 MicroBeads (Miltenyi Biotec, Bergisch Gladbach, Germany) following the manufacture's instruction. hLSECs and primary mouse LSECs were cultured in Endothelial Cell Growth Medium

(Cat# 211-500, Sigma) consisting of 5% fetal bovine serum (FBS), 1% endothelial cells growth supplement, and 1% primocin (InVivoGen, San Diego, CA) solution.

Mass Cytometry by time-of-light (CyTOF) analysis. Four to three random mice from each cohort were selected for intrahepatic leukocyte isolation by using a liver dissociation kit and Percoll gradient centrifugation. Cells were suspended in Maxpar Cell Staining Buffer (CSB) (Fluidigm, San Francisco, CA) and labeled with 0.5 µM cisplatin (Fluidigm) solution. After centrifugation, the cells were resuspended in CSB before the addition of the cell surface antibody staining cocktail in an equal volume of CSB. Cells were incubated with gentle agitation at room temperature for 45 min. Following a wash with CSB, samples were permeabilized with eBioscience FoxP3/Transcription factor/fixation/permeabilization solution (Thermo Fisher, Waltham, MA), washed, and resuspended in permeabilization buffer before the addition of the intracellular antibody staining cocktail. Cells were incubated at room temperature for 45 min with gentle agitation. Following washing with PBS, the cells were fixed with 2% paraformaldehyde using gentle agitation at 4°C for 30 min. Cells were washed with CSB and DNA intercalation was performed by adding 1:10000 diluted 125 µM of Cell ID™ Intercalator-Ir (Fluidigm) with gentle agitation at 4°C overnight. Cells were resuspended in a 1:10 dilution of EQ beads (EQ Four Element Calibration Beads, Fluidigm) and then loaded onto the Helios sample loader for data acquisition. Mass cytometry was performed in the Immune Monitoring Core at the Mayo Clinic, and antibodies conjugated to stable heavy metal isotopes were employed to detect cellular antigens by mass cytometry time-of-flight (CyTOF) and enable comprehensive profiling of the phenotype and function of intrahepatic leukocytes. After data acquisition, fcs files were normalized using CyTOF Software (version 6.7.1014). Cleanup of cell debris, removal of doublets, and dead cells were performed using FlowJo software version 10.5.3 (Ashland, OR). The cleaned fcs files were

analyzed using the R-based tool CyTOF kit version 3.8. Clustering and dimensionality reduction to 20,000 events per file were performed using the R-phonograph algorithm. The marker panel which was used in this study is shown in Supplementary Table 2, Fig. S4). Visualization of the clusters was performed using a tSNE map. Relative marker intensities and cluster abundances per sample were visualized using a heat map.

Isolation of human primary monocytes. Monocytes were isolated using the Pan Monocyte Isolation Kit (#130-096-537, Miltenyi Biotec, Bergisch Gladbach, Germany) according to the manufacturer's instructions. PBMCs were obtained from healthy adult volunteers in Mayo Clinic.

Differentiation of human primary dendritic cells. Following bead isolation, human monocytes were cultured in a 10 cm tissue culture dish for 5 days in RPMI 1640 media (11875093, ThermoFisher) with recombinant human GM-CSF (50ng/ml, 215-GM, R&D Systems) and recombinant human IL-4 (50ng/ml, BT-006, R&D Systems). Cells were resuspended in fresh RPMI media to use for the coculture assay.

Co-culture assay of human dendritic cells and human LSECs. hLSECs are harvested in 6-well tissue culture plate. At 90 % confluence, hLSECs were treated with 800μM palmitate ± 20nM LY overnight. The medium was then removed and hLSECs were cultured with human DCs (5.0×10^5 /well) in fresh LSEC growth medium for 6 hours (for cytokine staining) 48 hours (for surface marker staining). For the assay with conditioned medium, the medium was changed into LSEC growth medium after hLSECs are treated with 800μM palmitate ± 20nM LY overnight. After 24 hours, the medium was collected, and human DCs (5.0×10^5) were cultured with the medium for 6 hours (for cytokine staining) 48 hours (for surface marker staining). Cells were collected and stained with the antibodies for 30 min at 4 °C in the dark (Supplementary Table 1). The stained

cells were analyzed using ZE5 cell analyzer (Bio-Rad). The data were analyzed using FlowJo v10.8 software.

3D co-culture assay of human hepatic stellate cells and human LSECs. Endothelial cell growth medium was combined with Matrigel Matrix (Corning, NY, USA, Cat# 356231) in a 3:2 ratio to produce a 40% Matrigel solution. Each well of a 96-well culture plate was coated with 80 µl of the 40% Matrigel solution. The Matrigel layers were then incubated at 37°C for 45 minutes to enhance polymerization. 1.0×10^5 hLSECs and 1.0×10^5 human hepatic stellate cells (hHSCs) (#5300, ScienCell Research Laboratories, San Diego, CA) were suspended in endothelial cell growth medium and seeded on the freshly solidified Matrigel layer. After 3 day-treatment with or without palmitate (800 µM) and LY (20 nM), cells were recovered from Matrigel using Corning Cell Recovery Solution (Corning, Cat#35425), and total RNA extraction, cDNA synthesis, and qPCR analysis were performed.

Flow cytometry of human dendritic cells treated with CXCL10. Human dendritic cells (5.0×10^5 /well) were treated with recombinant CXCL10 (100 ng/mL and 500 ng/mL, R&D systems, 266-IP/CF) for 2 days. Cells were collected and stained with the antibodies for 30 min at 4 °C in the dark (Supplementary Table 1). The stained cells were analyzed using ZE5 cell analyzer (Bio-Rad). The data were analyzed using FlowJo v10.8 software.

Flow cytometry for intrahepatic leukocytes. One gram of liver tissue was dissociated using a mouse liver dissociation kit (Miltenyi Biotec, Bergisch Gladbach, Germany), according to the manufacturer's instructions. Intrahepatic leukocytes were purified by Percoll gradient centrifugation (Sigma-Aldrich). Cells were stained with the antibodies for 20 min at room temperature in the dark (Supplementary table 1). The stained cells were analyzed using ZE5 cell analyzer (Bio-Rad). The data were analyzed using FlowJo v10.8 software.

Seahorse assay. The Seahorse XF Analyzer (Agilent Technologies) was used to assess the mitochondrial bioenergetics of hLSECs. Prior to the assay, assay cartridges were hydrated in Seahorse XF Calibrant at 37°C in a non-CO2 incubator overnight. hLSECs were cultured at a density of 20,000 cells per well. Cells were allowed to adhere overnight at 37°C in a 5% CO2 incubator. After pretreatment of LY or DMSO for 1 hour, cells were treated with either 500 µM PA ± 20 nM of LY or DMSO for 4 hours. Cells were washed and incubated in Seahorse XF Assay Medium (Agilent Technologies) supplemented with 25 mM glucose, 1 mM pyruvate, and 2 mM glutamine at 37°C in a non-CO2 incubator for 1 hour prior to starting the assay. 1 µM oligomycin, 0.25 µM FCCP and 1 µM rotenone–antimycin-A were loaded in the assay wells. The oxygen consumption rate (OCR) and extracellular acidification rate (ECAR) measurements for each well were recorded and reported as pmol/min and mpH/min, respectively. Results were displayed as a percentage of maximal OCR and ECAR or as relative fold change of each parameter between time points.

Precision-Cut Liver Slices (PCLS) Preparation and Treatment. Human liver tissue was collected from patients who provided consent for liver resections at the Mayo Clinic and processed immediately, as previously described (12, 13). Following resection, the tissue was immediately transported in RPMI medium (11875085, Gibco) at 4°C to minimize ischemic injury. The liver capsule and any cauterized scar tissue were carefully removed to isolate fresh tissue. The tissue was then sectioned into ~8 mm x 8 mm wedges and embedded in 2.5% low-melting-point agarose (VF-AGT-VM, Precisionary). The embedded tissue was sliced into 250 µm-thick sections using a Compresstome instrument (VF-510-0Z, Precisionary), following the manufacturer's recommended blade oscillation and speed settings. Slices were temporarily stored in cold HBSS on ice until sample processing was completed. PCLS samples were then transferred to sterile

culture media consisting of a 1:1 mixture of Williams' E Medium (12551032, Gibco) and DMEM (#11995-065, Gibco), supplemented with 10% fetal bovine serum (#s11150H, R&D Systems), 1% penicillin-streptomycin (15140122, Gibco), and Insulin-Transferrin-Selenium-Ethanolamine (ITS-X, #51500056, Gibco). To mitigate stress-induced signaling from the slicing process, the medium was supplemented with 25 mM valproic acid (P4543, Sigma-Aldrich), 94 nM SB431542 (1614, Tocris Bioscience), and 6.7 nM CHIR99021 (4423, Tocris Bioscience) for the first 24 h. After that, slices were incubated for an additional 96 h in control media supplemented with gentamycin (50 µg/mL; Cat. 15750-037, Invitrogen) and glucose (25 mM; Cat. 1.08342.1000, Merck, Darmstadt, Germany), or MASLD-inducing media referred to as GFIPO media (14) supplemented with additional glucose (36 mM), fructose (5 mM, Cat. F3510-100G, Sigma-Aldrich, St. Louis, MO, USA), human insulin (1 nM, Cat. I9278 (Sigma-Aldrich Louis, MO, USA), palmitic acid (240 µM, Cat. P0500-10G, Sigma-Aldrich, St. Louis, MO, USA) and oleic acid (480 µM, Cat. 75096-1L, Sigma-Aldrich, St. Louis, MO, USA) with or without 20 nM GSK3 inhibitor LY. Slices were cultured at 37°C with 5% CO_2 on a rocking platform (90 rpm) to ensure adequate nutrient and oxygen exchange. The medium was refreshed daily. Following treatment, RNA and proteins were isolated for further analysis.

RNA sequencing and bioinformatic analysis. RNA sequencing was performed on hLSECs treated with PA overnight at the Mayo Clinic Genome Analysis Core. Three replicates per group were included for the study. RNA libraries were prepared using 200 ng of total RNA according to the manufacturer's instructions for the TruSeq RNA Sample Prep Kit v2 (Illumina, San Diego, CA). The concentration and size distribution of the completed libraries was determined using an Agilent Bioanalyzer DNA 1000 chip (Santa Clara, CA) and Qubit fluorometry (Invitrogen, Carlsbad, CA). Libraries were sequenced at 53 million to 90 million reads per sample following Illumina's

standard protocol using the Illumina cBot and HiSeq 3000/4000 PE Cluster Kit. The flow cells were sequenced as 100 × 2 paired end reads on an Illumina HiSeq 4000 using HiSeq 3000/4000 sequencing kit and HCS v3.3.20 collection software. Base calling was performed using Illumina's RTA version 2.5.2. Genes with Log2 fold change (Log2FC) more than 1.5 and p-value less than 0.05 were considered differentially expressed. Gene expression profiles of isolated LSECs from mice were assessed using a NanoString nCounter® system (NanoString Technologies, Seattle, WA, USA). We employed a Myeloid Innate Immunity Panel of isolated LSECs from CDHFD-fed mice. LSECs were isolated as reported previously (15). Total RNA was isolated from isolated LSECs using the Zymo Research Direct-zol RNA MiniPrep Kit (Zymo Research, Irvine, CA, USA) according to the manufacturer's instructions. The quantity and quality of extracted RNA were assessed using a NanoDrop spectrophotometer (Thermo Fisher Scientific, Waltham, MA, USA). For the NanoString nCounter® assay, 100 ng of total RNA was hybridized at 65°C for 18 h in a thermal cycler with a custom-designed code-set specific for the target genes of interest, including housekeeping genes for normalization, according to the manufacturer's instructions. Raw data were normalized to internal positive controls and housekeeping genes to account for the variability in RNA input and hybridization efficiency. Data analysis was performed using nSolver™ Analysis Software (NanoString Technologies). Only samples that passed the Nanostring quality control (QC) assessment were included in the analysis; samples with QC flags were excluded. Differential gene expression analysis was performed to identify significant differences between the experimental groups.

Ingenuity Pathway Analysis (IPA) software was used to analyze the transcriptomic data from NanoString and bulk-RNA sequence. Subsequently, these differentially expressed genes were grouped into gene pathways using the pathway enrichment analysis with the Kyoto Encyclopedia

of Genes and Genomes database (KEGG; www.genome.jp/kegg). The statistically enriched differentially expressed genes in KEGG pathways were considered significant with $p < 0.05$. KEGG pathway enrichment analysis was conducted using R version 4.5.0 (R Foundation for Statistical Computing, Vienna, Austria). Heatmaps were generated using ClustVis, a web tool for visualizing clustering of multivariate data (https://biit.cs.ut.ee/clustvis/).

Dendritic cell quantification of within liver tissue sections of patients with MASH. Digital slide scanning and analysis were performed to phenotype and spatially resolve cell populations within liver tissue sections. Immunofluorescent images were imported and managed using the open-source bioimage analysis software, QuPath (version 0.6.0). An initial annotation using a pixel classifier was created for each slide to delineate the entire liver tissue section. Regions exhibiting tissue folding artifacts or significant loss of immunofluorescence signal were manually excluded from this primary annotation to ensure data quality. Within the primary tissue area, portal tracts were identified and excluded from the analysis to focus on the liver parenchyma. This was achieved by training a pixel-based Random Forest classifier, a supervised machine learning algorithm well-established for tissue segmentation in digital pathology. The classifier was trained on manually annotated representative regions of portal and non-portal tissue to generate a pixel classifier capable of accurately segmenting these distinct tissue compartments. Following the exclusion of portal areas, individual cell nuclei were detected within the remaining parenchymal tissue using the DAPI staining channel. For this task, the deep-learning-based InstanSeg algorithm.(16), integrated into QuPath as an extension, was employed for robust and accurate instance segmentation of nuclei. Once nuclei were segmented, a cell classification script was executed to phenotype cells based on their marker expression. This was achieved by employing multiple steps of segmentation using the InstanSeg algorithm. Based on this, detected cells were

classified into distinct populations: a. LSECs were identified based on positivity for CD14, b. DCs were defined by dual positivity for both CD11c and HLA-DR. Upon completion of the image analysis pipeline, tissue surface area, cellular classifications, and spatial coordinates (x/y positions), were exported from QuPath. This data was then imported into a custom Python script for quantitative spatial analysis. The SciPy library (17) was utilized to compute a spatial score to characterize the distribution and proximity relationships between the identified cell populations.

2. Supplementary Tables

Supplementary Table 1. Flow cytometry panel.

1. Mouse dendritic cells

Marker	Fluorochrome	Catalog #	Company
CD45	Vio-green	130-110-803	Miltenyi Biotec
CD11b	PE-vio770 REA	130-113-808	Miltenyi Biotec
MHC II	PerCP-vio700 REA	130-103-875	Miltenyi Biotec
CD11c	APC-vio 770 Rat	130-110-841	Miltenyi Biotec
CD86	PE REA	130-123-724	Miltenyi Biotec
XCR1	APC REA	130-111-373	Miltenyi Biotec

2. Mouse macrophages

Marker	Fluorochrome	Catalog #	Company
CD45	Vio-green	130-110-803	Miltenyi Biotec
CD11b	PerCP-vio700 REA	130-113-809	Miltenyi Biotec
F4/80	APC-cy7 Rat	123117	BioLegend
Ly6G	PE REA	130-123-780	Miltenyi Biotec

3. Human dendritic cells (Maturation panel)

Marker	Fluorochrome	Catalog #	Company
CD11c	PE-Cy7	301607	BioLegend
MHC II	FITC	327005	BioLegend
CD80	PerCP Cy5.5	375411	BioLegend
CD86	BV421	305417	BioLegend
CD83	APC-Cy7	305329	BioLegend
CD14	BV650	367163	BioLegend

CD 40	AF700	334327	BioLegend
AXL	APC	386205	BioLegend
CCR7	PE	353203	BioLegend

3. Human dendritic cells (Cytokine panel)

**Marker**	**Fluorochrome**	**Catalog #**	**Company**
CD11c	PE-Cy7	301607	BioLegend
MHC II	AF700	327013	BioLegend
IL12p70	PE	559325	BD Biosciences
IL10	APC	506806	BioLegend
TNFa	FITC	502906	BioLegend

Supplementary Table 2. CyTOF Panel.

**Label**	**Target**	**Clone**	**Company**	**Catalog Number**	**Assigned Category (for gating)**
089Y	CD45	30-F11	Fluidigm	3089005B	Leukocytes
106Cd	CD146	ME-9F1	Biolegend	134702	Endothelial cells
110Cd	XCR1	ZET	Biolegend	148202	Dendritic cells (cDC1)
111Cd	CD11b (Mac-1)	M1/70	Fluidigm	3172012B	Myeloid lineage cells
112Cd	CD4	RM4-5	Biolegend	100561	CD4+ T cells
113Cd	Ly6G	1A8	Biolegend	127637	Granulocytes
114Cd	MARCO	2359A	R&D	MAB29561-100	Kupffer cells/tissue-resident macrophages
139La	Spp1	E9Z1D	CST	17090	Lipid-associated or tissue-remodeling macrophage
141Pr	Lgals3	202213	R&D Systems	MAB1197	Macrophages
142Nd	CD11c	N418	Fluidigm	3142003B	Monocytes/Dendritic cells
143Nd	TCRb	H57-597	Fluidigm	3143010B	T cells

144Nd	MHC Class I	28-14-8	Fluidigm	3144016B	Nucleated cells
145Nd	CLEC-2 (CLEC1B)	17D9/CLEC-2	Biolegend	146102	Monocyte-derived macrophages
146Nd	CD40L	24-31	Biolegend	310835	Activated CD4+ T cells (co-stimulation)
147Sm	CD80	16-10A1	Biolegend	104702	Antigen-presenting cells (co-stimulatory marker)
148Nd	CXCR2	SA044G4	Biolegend	149302	Neutrophils
149Sm	Tim4	RMT4-54	Biolegend	130002	Kupffer cells
151Eu	CD206 (MMR)	C068C2	Biolegend	141702	Restorative macrophages
152Sm	CD3	145-2C11	Fluidigm	3152004B	T cells
153Eu	CLEC4F/CLECSF13	poly Goat	R&D	AF2784	Kupffer cells
154Sm	CD62L (L-selectin)	MEL-14	Biolegend		Lymphocytes
155Gd	MERTK	108928	R&D Systems	MAB5912	Macrophages
156Gd	CCR2	475301	R&D Systems	MAB55381	Pro-inflammatory monocytes
158Gd	CD40	1C10	Biolegend	102802	Activated antigen-presenting cells
159Tb	F4/80	BM8	Fluidigm	3159009B	Macrophages
160Gd	CD64	290322	R&D Systems	MAB20741	Macrophages
161Dy	CD83	Michel-19	Biolegend	121503	Maturation marker
163Dy	TGFb	TW4-6H10	Standard BioTools	3163010B	Immunoregulatory marker
164Dy	CX3CR1	SA011F11	Fluidigm	3164023B	Monocytes/Macrophages
165Ho	CD14	Sa14-2	Biolegend	123321	Monocytes/Macrophages
166Er	CD19	6D5	Fluidigm	3166015B	B cells
167Er	TREM2	237920	R&D	MAB17291	Scar-associated macrophages
168Er	CD8a	53-6.7	Fluidigm	3168003B	CD8+ T cells
169Tm	CD163	S15049	Biolegend	155302	Restorative macrophages
170Er	CD161 (NK1.1)	PK136	Fluidigm	3170002B	NK cells

171Yb	CD86	GL-1	Biolegend	105002	Antigen-presenting cells (co-stimulatory marker)
173Yb	S100A8	63N13 G5	NOVUS	NBP2-25273	Granulocytes
174Yb	CD115/CSF1R	AFS98	Biolegend	135521	Macrophages
175Lu	Ly6C	HK1.4	Biolegend	128039	Monocytes
176Yb	CD45R (B220)	RA3-6B2	Fluidigm	3176002B	B cells
209Bi	MHC II	M5/11 4.15.2	Biolegend	107637	Antigen presenting cells

Supplementary Table 3. **Predominant immune cell markers for the differentially expressed clusters between the study groups.**

Cluster	Predicted cell type	Marker profile
Cluster 1	Restorative macrophages	$CD206^{high}$, MHC II^{high}, $CLEC2^{+}$, $CD80^{+}$, $CD83^{+}$, $TGF\beta^{+}$, $CD64^{+}$, $MERTK^{+}$
Cluster 2	Naïve B cells	MHC II^{high}, $B220^{+}$, $CD80^{-}$, $CD86^{-}$,
Cluster 3	Immature DC	$Ly6C^{high}$, $CD11c^{+}$, MHC II^{+}, $CD11b^{-}$, $CD80^{-}$, $CD86^{-}$, $XCR1^{-}$
Cluster 5	Naïve/immature B cells	MHC II^{high}, $CCR2^{+}$, $CD19^{+}$, $B220^{+}$, $CD80^{-}$, $CD86^{-}$,
Cluster 6	Naïve B cells	MHC II^{high}, $CD19^{+}$, $B220^{+}$, $CD80^{-}$, $CD86^{-}$,
Cluster 7	Neutrophils	$Ly6C^{high}$, $CD11b^{high}$, $Ly6G^{high}$, $S100A8^{+}$, $CD18^{+}$, $CD83^{+}$
Cluster 9	Type 1 conventional dendritic cells	MHC II^{high}, $CD11c^{high}$, $CD18^{+}$, $CD86^{+}$, $TGF\beta^{+}$, $XCR1^{+}$
Cluster 11	Neutrophils	$Ly6C^{high}$, $CD11b^{high}$, $Ly6G^{high}$, $S100A8^{+}$, $CD18^{+}$, $CD83^{+}$
Cluster 12	Lipid-associated macrophages	$CD64^{high}$, MHC II^{high}, $F4/80^{+}$, $CD163^{+}$, $TREM2^{+}$, $SPP1^{+}$, $Lgals3^{+}$
Cluster 13	Neutrophils	$Ly6G^{high}$, $Ly6C^{high}$, $CD11b^{high}$, $S100A8^{+}$
Cluster 14	$CD8^{+}$ T cells	$Ly6C^{high}$, $CD8a^{high}$, $TCRb^{+}$
Cluster 15	Kupffer cells	$CD11b^{high}$, $F4/80^{high}$, $Tim4^{+}$, $MARCO^{+}$
Cluster 17	Pro-inflammatory $Ly6C^{high}$ monocytes	$Ly6C^{high}$, $CD11b^{high}$, $TGF\beta^{+}$, $CX3CR1^{+}$, $CCR2^{+}$, $CD18^{+}$, $F4/80^{+}$
Cluster 18	Kupffer cells	MHC II^{high}, $CLEC4F^{high}$, $CLEC2^{high}$, $CD18^{high}$, $Tim4^{high}$, $F4/80^{high}$, $MARCO^{+}$
Cluster 19	$CD8^{+}$ T cells	$CD8a^{high}$, $TCRb^{+}$
Cluster 20	Monocyte-derived macrophages	$CD11b^{high}$, $CD18^{high}$, $TGF\beta^{+}$, $CX3CR1^{+}$, $Ly6C^{+}$, $CD11c^{+}$, $F4/80^{+}$
Cluster 21	Monocyte-derived macrophages	MHC II^{high}, $CD11b^{high}$, $CD11c^{high}$, $CD18^{high}$, $F4/80^{high}$, $CD86^{high}$, $TGF\beta^{+}$, $CX3CR1^{+}$, $CD80^{+}$, $CD86^{+}$
Cluster 23	Type 2 conventional dendritic cell	MHC II^{high}, $CD11b^{high}$, $CD11c^{high}$, $TGF\beta^{+}$, $CD18^{+}$, $CD86^{+}$
Cluster 24	Monocyte-derived macrophage	$CD11b^{high}$, $TGF\beta^{+}$, $Ly6C^{+}$, $CD18^{+}$, $F4/80^{+}$
Cluster 25	Activated monocyte-derived dendritic cells	MHC II^{high}, $CD11b^{high}$, $Ly6C^{high}$, $TGF\beta^{+}$, $CX3CR1^{+}$, $CCR2^{+}$, $CD11c^{+}$, $CD18^{+}$, $CD80^{+}$, $CD86^{+}$, $XCR1^{-}$

Twenty of the twenty-five clusters were categorized into distinct leukocyte subpopulations based on the intensities of individual cell markers. The remaining seven clusters could not be categorized due to the absence of essential markers required to define a distinct subpopulation.

Supplementary Table 4. **Primer list.**

Gene Name		Sequence
18S Forward Primer		CGCTTCCTTACCTGGTTGAT
18S Reverse Primer		GAGCGACCAAAGGAACCATA
Mouse *Ccl2* Forward Primer		TTAAAAACCTGGATCGGAACCA
Mouse *Ccl2* Reverse Primer		GCATTAGCTTCAGATTTACGGG
Mouse *Col1a1* Forward Primer		GCTCCTCTTAGGGGCCACT
Mouse *Col1a1* Reverse Primer		CCACGTCTCACCATTGGGG
Mouse *Cxcl1* Forward Primer		CTGGGATTCACCTCAAGAACATC
Mouse *Cxcl1* Reverse Primer		CAGGGTCAAGGCAAGCCTC
Mouse *Cxcl2* Forward Primer		CCAACCACCAGGCTACAGG
Mouse *Cxcl2* Reverse Primer		GCGTCACACTCAAGCTCTG
Mouse *Cxcl10* Forward Primer		CCAAGTGCTGCCGTCATTTTC
Mouse *Cxcl10* Reverse Primer		GGCTCGCAGGGATGATTTCAA
Mouse *Il1b* Forward Primer		GCAACTGTTCCTGAACTCAACT
Mouse *Il1b* Reverse Primer		ATCTTTTGGGGTCCGTCAACT
Mouse *Il6* Forward Primer		TAGTCCTTCCTACCCCAATTTCC
Mouse *Il6* Reverse Primer		TTGGTCCTTAGCCACTCCTTC
Mouse *Mmp13* Forward Primer		CTTCTTCTTGTTGAGCTGGACTC
Mouse *Mmp13* Reverse Primer		CTGTGGAGGTCACTGTAGACT
Mouse *Myc* Forward Primer		GCGACTCTGAAGAAGAGCAAG
Mouse *Myc* Reverse Primer		GCCTCGGGATGGAGATGAG
Mouse *Nfkbia* Forward Primer		CTCCGAGACTTTCGAGGAAATAC

Mouse *Nfkbia* Reverse Primer		GCCATTGTAGTTGGTAGCCTTCA
Mouse *Tgfb* Forward Primer		CTCCCGTGGCTTCTAGTGC
Mouse *Tgfb* Reverse Primer		GCCTTAGTTTGGACAGGATCTG
Mouse *Tnfa* Forward Primer		CCCTCACACTCAGATCATCTTCT
Mouse *Tnfa* Reverse Primer		GCTACGACGTGGGCTACAG
Mouse *Tnfaip3* Forward Primer		GAACAGCGATCAGGCCAGG
Mouse *Tnfaip3* Reverse Primer		GGACAGTTGGGTGTCTCACATT
Mouse *Vcam1* Forward Primer		TCTTGGGAGCCTCAACGGTA
Mouse *Vcam1* Reverse Primer		CAAGTGAGGGCCATGGAGTC
Human *CCL2* Forward Primer		CAGCCAGATGCAATCAATGCC
Human *CCL2* Reverse Primer		TGGAATCCTGAACCCACTTCT
Human *CXCL1* Forward Primer		TCATCGAAAAGATGCTGAACA
Human *CXCL1* Reverse Primer		TTCAGGAACAGCCACCAGT
Human *CXCL2* Forward Primer		CATCGAAAAGATGCTGAAAAATG
Human *CXCL2* Reverse Primer		TTCAGGAACAGCCACCAATA
Human *CXCL10* Forward Primer		CCAAGTGCTGCCGTCATTTTC
Human *CXCL10* Reverse Primer		GGCTCGCAGGGATGATTTCAA
Human *ICAM1* Forward Primer		TATAAAGGATCACGCGCCCC
Human *ICAM1* Reverse Primer		GACTCACCTGGGAACAGAGC
Human *IL1B* Forward Primer		ATGATGGCTTATTACAGTGGCAA
Human *IL1B* Reverse Primer		GTCGGAGATTCGTAGCTGGA
Human *IL6* Forward Primer		ACTCACCTCTTCAGAACGAATTG
Human *IL6* Reverse Primer		CCATCTTTGGAAGGTTCAGGTTG

Human *MFN2* Forward Primer		CTCTCGATGCAACTCTATCGTC
Human *MFN2* Reverse Primer		TCCTGTACGTGTCTTCAAGGAA
Human *MYC* Forward Primer		ACGGCCGACCAGCTGGAGAT
Human *MYC* Reverse Primer		TCGGGCTGCCGCTGTCTTTG
Human *NFKBIA* Forward Primer		CTCCGAGACTTTCGAGGAAATAC
Human *NFKBIA* Reverse Primer		GCCATTGTAGTTGGTAGCCTTCA
Human *NRF1* Forward Primer		AGGAACACGGAGTGACCCAA
Human *NRF1* Reverse Primer		TATGCTCGGTGTAAGTAGCCA
Human *OPA1* Forward Primer		TGTGAGGTCTGCCAGTCTTTA
Human *OPA1* Reverse Primer		TGTCCTTAATTGGGGTCGTTG
Human *PDGFRB* Forward Primer		AGCACCTTCGTTCTGACCTG
Human *PDGFRB* Reverse Primer		TATTCTCCCGTGTCTAGCCCA
Human *TFAM* Forward Primer		ATGGCGTTTCTCCGAAGCAT
Human *TFAM* Reverse Primer		TCCGCCCTATAAGCATCTTGA
Human *TNFAIP3* Forward Primer		CGTCCAGGTTCCAGAACACCATTC
Human *TNFAIP3* Reverse Primer		TGCGCTGGCTCGATCTCAGTTG

3. Supplementary figures

Figure S1

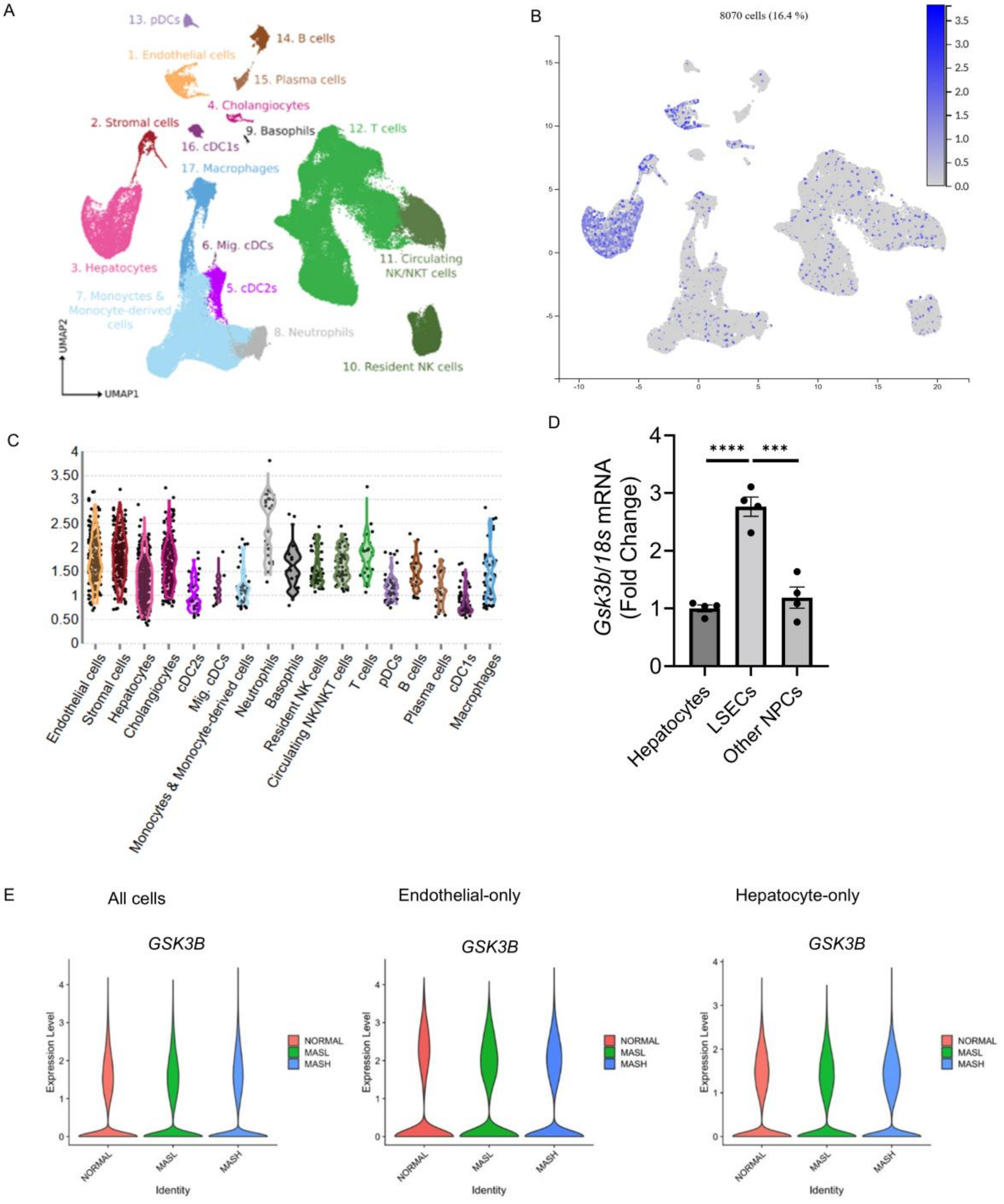

Figure S1. *GSK3β* expression in human liver cell populations. (A) The UMAP plot shows clustering of distinct liver cell populations. (B) The UMAP plot displaying *Gsk3β* expression across liver cell types, color coded by expression level (gray to blue). The number and percentage of *Gsk3β*-expressing cells are indicated. (C) Violin plot illustrating *Gsk3β* expression across annotated liver cell types. Each dot represents a single cell. Figures are generated by Liver Cell Atlas (https://www.livercellatlas.org). (D) Gsk3β expression of each cell type isolated from wild type mice. (E) Violin plots showing the expression of *GSK3β* based on human single-nuclear RNA sequencing data (GEO accession: GSE244832). Expression levels are compared among normal subjects (red), patients with metabolic dysfunction-associated steatotic liver (MASL; green), and patients with metabolic dysfunction-associated steatohepatitis (MASH; blue). The panels display gene expression profiles across all captured cells (left), endothelial cells only (middle), and hepatocytes only (right). The y-axis represents the normalized expression level of GSK3β. Bar graphs represent the mean ± SEM. Statistical significance: *p <0.05; **p <0.01; ***p <0.001; ****p <0.0001; ns, non-significant (one-way ANOVA with Bonferroni's multiple comparison).

Figure S2

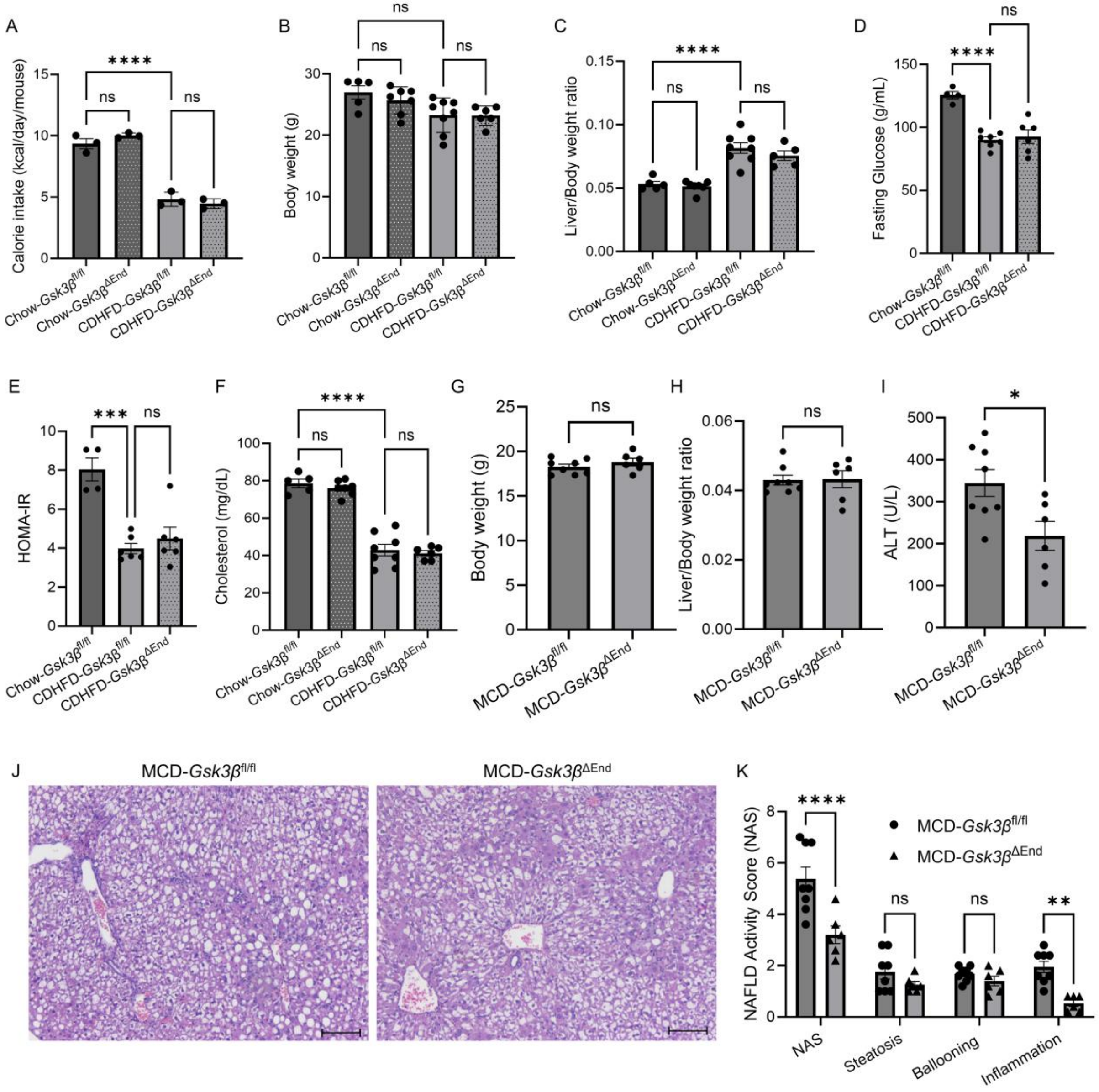
A
Calorie intake (kcal/day/mouse)
Chow-Gsk3β fl/fl
Chow-Gsk3β ΔEnd
CDHFD-Gsk3β fl/fl
CDHFD-Gsk3β ΔEnd
B
Body weight (g)
C
Liver/Body weight ratio
D
Fasting Glucose (g/mL)
E
HOMA-IR
F
Cholesterol (mg/dL)
G
Body weight (g)
MCD-Gsk3β fl/fl
MCD-Gsk3β ΔEnd
H
Liver/Body weight ratio
I
ALT (U/L)
J
MCD-Gsk3β fl/fl
MCD-Gsk3β ΔEnd
K
NAFLD Activity Score (NAS)
NAS
Steatosis
Ballooning
Inflammation
ns
*
**
***
****

Figure S2. Genetic deletion of *Gsk3β* in LSECs attenuates murine MASH. (A) Caloric intake (kcal/day/mouse) averaged over three days. (B) Body weight (g). (C) Liver/Body weight ratio. (D) Fasting glucose (mg/dL) after 12 hours fasting. (E) HOMA-IR calculated from fasting glucose and insulin. (F) Serum cholesterol (mg/dL). (G-I) Endothelial cell-specific *Gsk3β* deletion ($Gsk3\beta^{\triangle End}$) mice were fed the methionine and choline deficient diet (MCD) for 3 weeks to induce MASH. Body weight (G), Liver/Body weight ratio (H), alanine aminotransferase (ALT) (I) were measured at the endpoint. (J) Representative images of hematoxylin and eosin-stained liver sections (scale bar: 100 μm). (K) NAS and its components (Steatosis, Ballooning, and Inflammation). Bar graphs represent the mean ± SEM. Statistical significance: *p <0.05; **p <0.01; ***p <0.001; ****p <0.0001; ns, non-significant (one-way ANOVA with Bonferroni's multiple comparison for A, B, C, D; unpaired t-test for G, H, I; two-way ANOVA with Bonferroni's comparison for K).

Figure S3

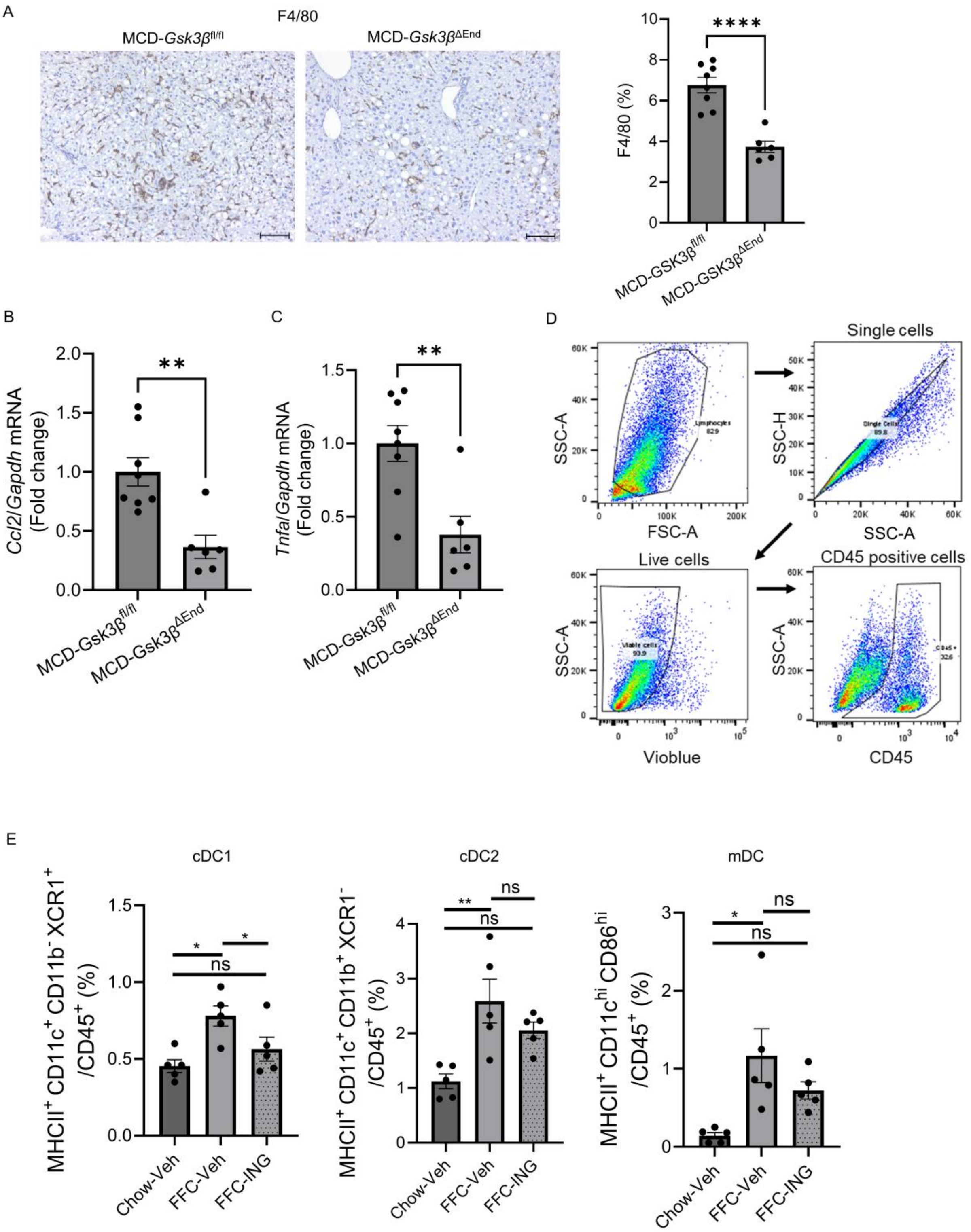

A
F4/80
MCD-Gsk3β fl/fl
MCD-Gsk3β ΔEnd
F4/80 (%)
****
MCD-GSK3β fl/fl
MCD-GSK3β ΔEnd
B
Ccl2/Gapdh mRNA (Fold change)
**
MCD-Gsk3β fl/fl
MCD-Gsk3β ΔEnd
C
Tnfa/Gapdh mRNA (Fold change)
**
MCD-Gsk3β fl/fl
MCD-Gsk3β ΔEnd
D
Single cells
Live cells
CD45 positive cells
SSC-A
SSC-H
FSC-A
Vioblue
CD45
Lymphocytes
Single Cells
Viable cells
E
cDC1
cDC2
mDC
MHCII+ CD11c+ CD11b- XCR1+ /CD45+ (%)
MHCII+ CD11c+ CD11b+ XCR1- /CD45+ (%)
MHCII+ CD11chi CD86hi /CD45+ (%)
*
**
ns
Chow-Veh
FFC-Veh
FFC-ING

Figure S3. GSK3β inhibition reduces liver inflammation by suppressing the recruitment of pro-inflammatory myeloid cells in murine MASH. (A) Representative immunohistochemical staining for F4/80 (left panel). Scale bar:100μm. Positive areas were quantified in five random 10x microscopic fields (right panel). (B, C) Whole liver mRNA expression of *Ccl2* (B) and *Tnfα* (C). (D)The gating strategy for CD45 positive cells. (E) Percentages of cDC1, cDC2, and mDC out of $CD45^+$ intrahepatic leukocytes. Mice were fed a high fat, fructose, and cholesterol (FFC) diet for 24 weeks and treated with the oral formulation of the GSK3 inhibitor 9-ING-41 (Elraglusib, 100 mg/kg/day) for 2 weeks. Bar graphs represent the mean ± SEM. Statistical significance: *p <0.05; **p <0.01; ***p <0.001; ****p <0.0001; ns, non-significant (unpaired t-test for A, B C; one-way ANOVA with Bonferroni's multiple comparison for E).

Figure S4

A

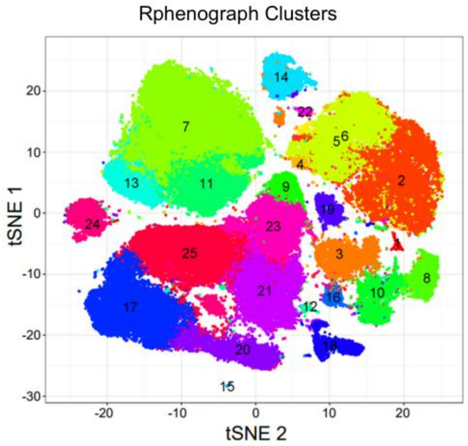

B

Markers of Myeloid Lineage Cells and Kupffer Cells

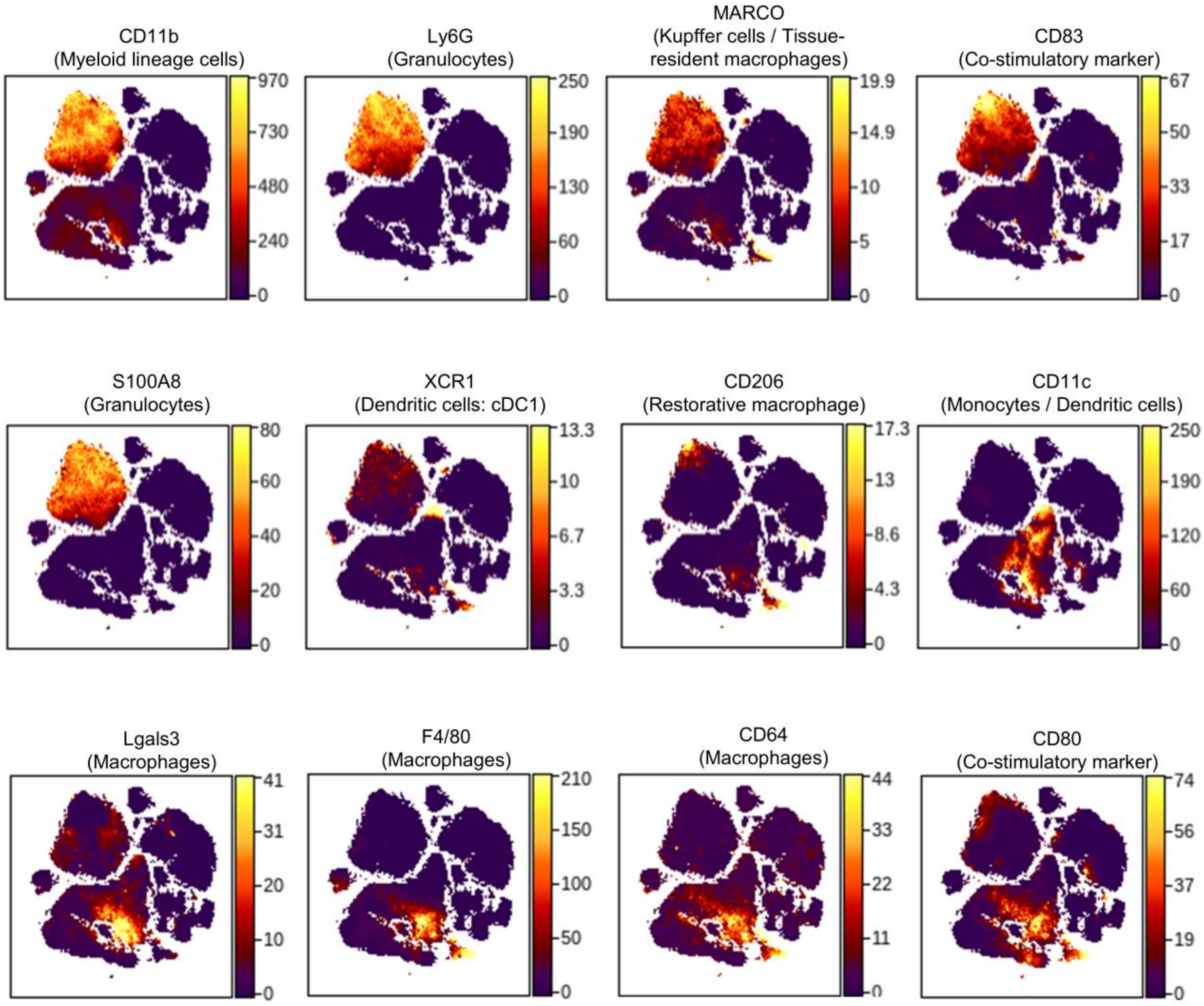

Figure S4 (continued)

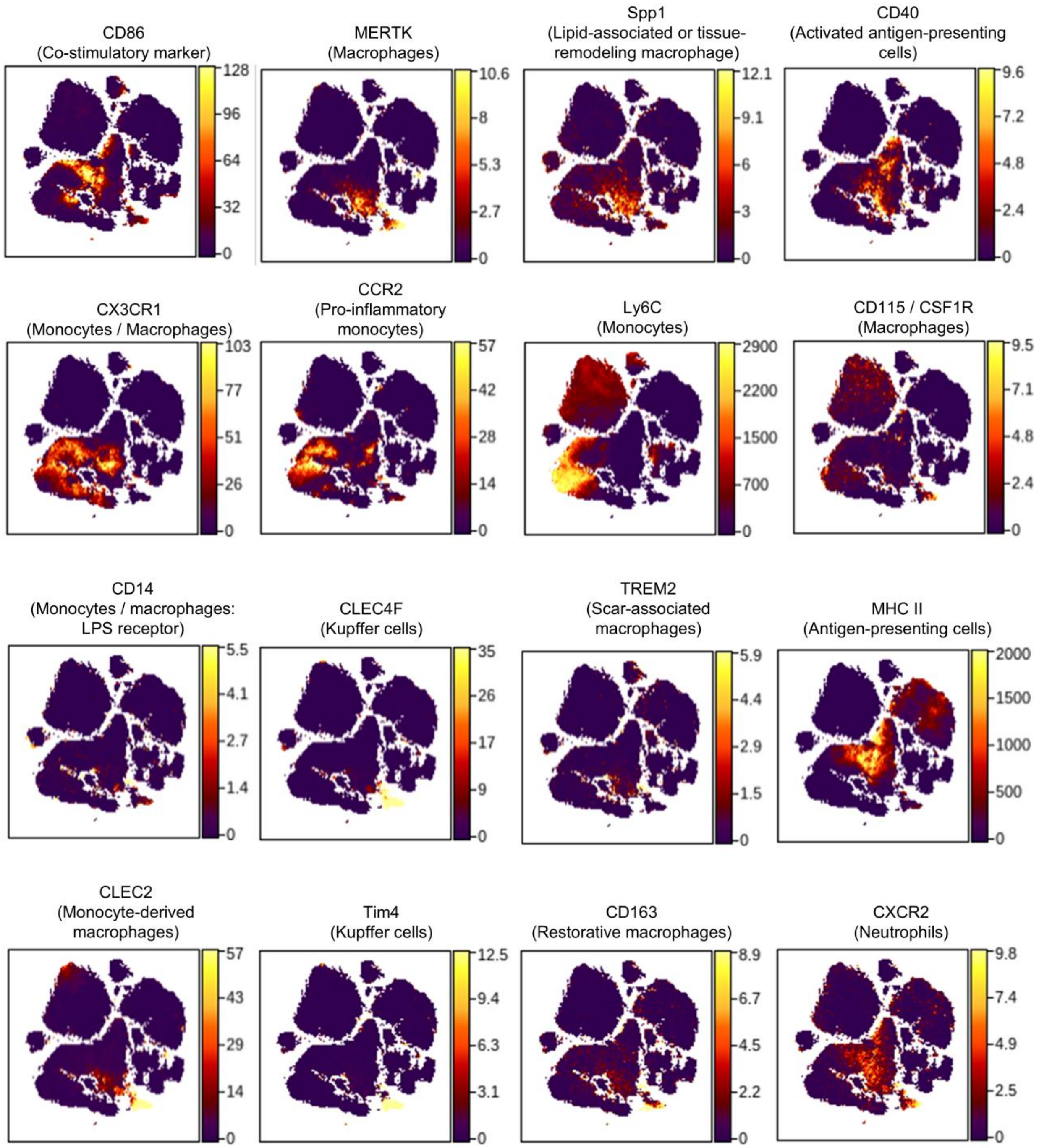

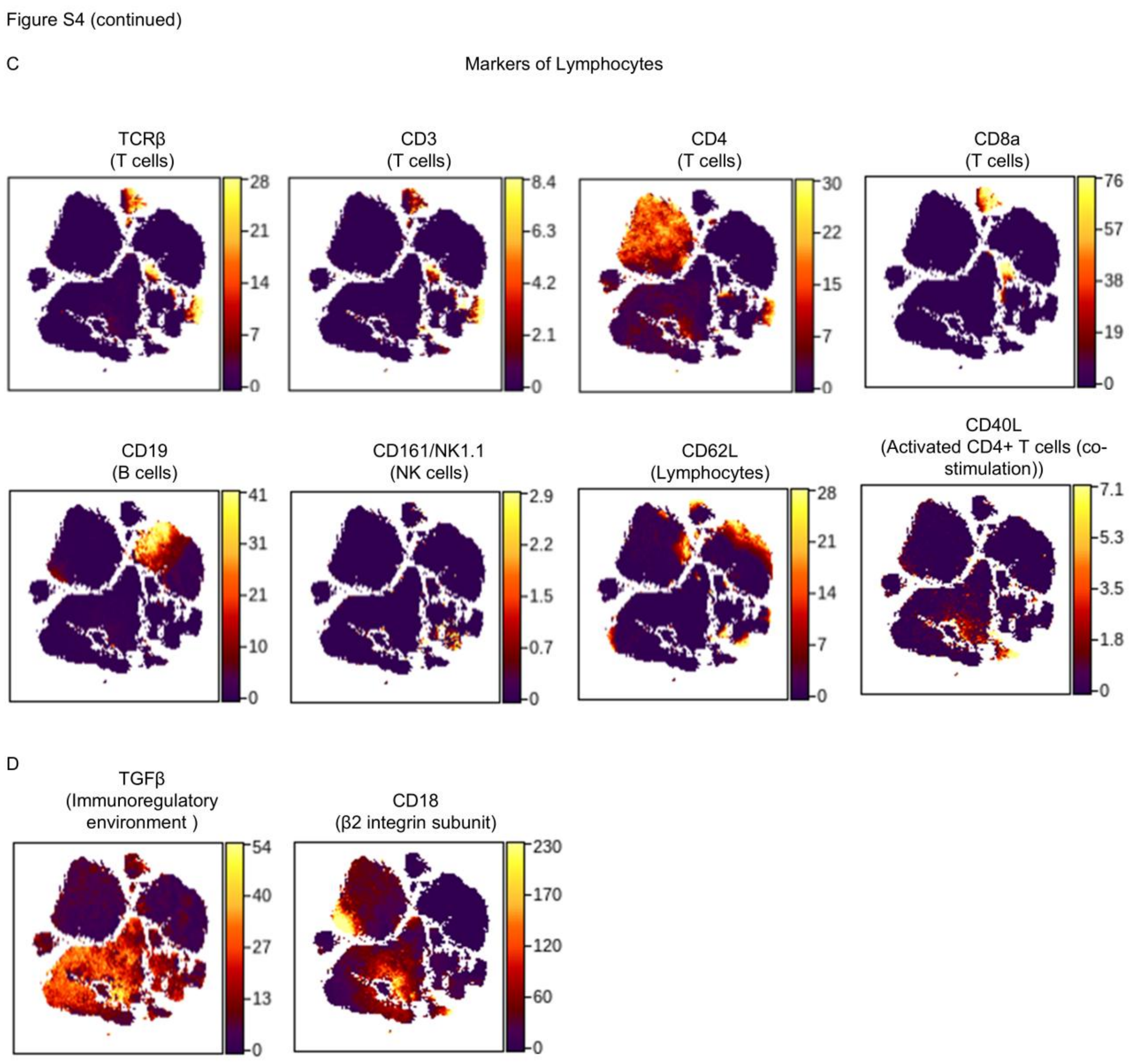

Figure S4. Intrahepatic leukocyte profiling by mass cytometry by time-of-flight (CyTOF).

CyTOF analysis of intrahepatic leukocytes from control chow-fed mice, and CDHFD-fed $\mathit{Gsk3\beta}^{fl/fl}$ and $\mathit{Gsk3\beta}^{\triangle End}$ mice. (A) t-distributed stochastic neighbor embedding (tSNE) plot generated using Rphenograph clustering algorithm (identical to Figure 3A). (B-D) tSNE plots showing marker densities: (B) myeloid-lineage cells and Kupffer cells and, (C) lymphocytes, (D) TGFβ and CD18.

Figure S5

Myeloid Cells

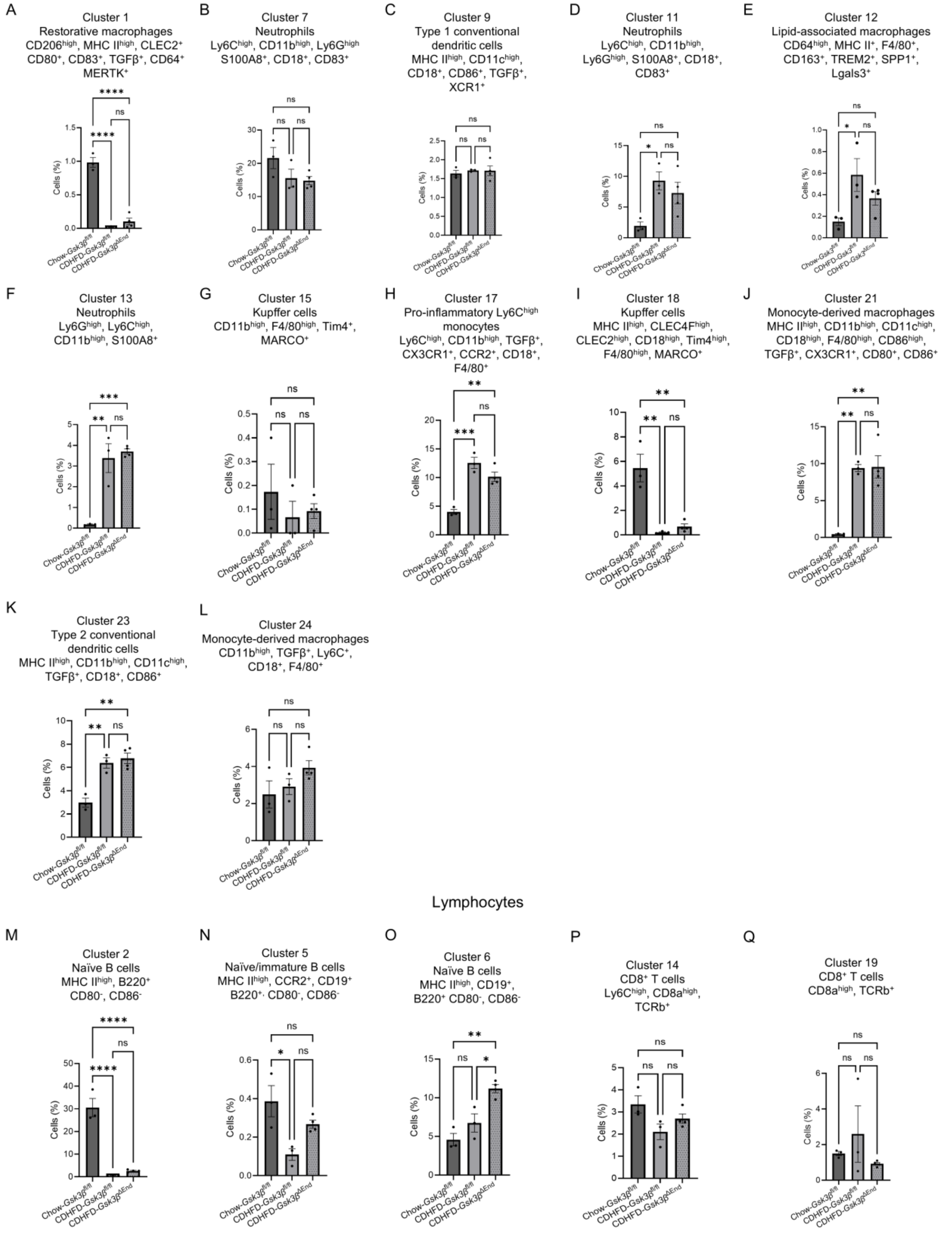

Figure S5. Endothelial cell *Gsk3β* deletion reduces activated monocyte-derived dendritic cells and monocytes derived inflammatory macrophages hepatic infiltration in CDHFD-fed mice. (A-Q) Quantification of CyTOF-defined clusters categorized into distinct leukocyte subpopulations based on surface markers intensities. Bar graphs show mean ± SEM. Statistical significance: *p <0.05; **p <0.01; ***p <0.001; ****p <0.0001; ns, non-significant (one-way ANOVA with Bonferroni's multiple comparison).

Figure S6

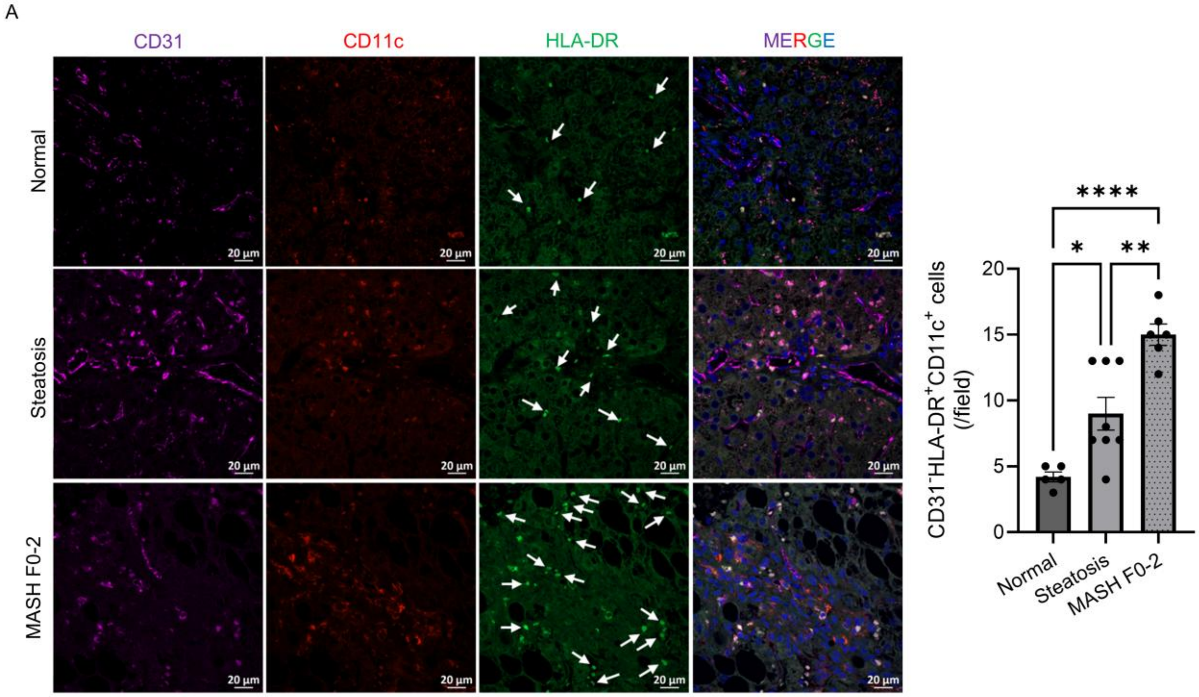

Figure S6. Human MASH liver shows increased dendritic cell infiltration. (A) Immunostaining images from patients with isolated steatosis and metabolic dysfunction-associated steatohepatitis (MASH) showing CD31 (a marker for liver sinusoidal endothelial cells), CD11c, and HLA-DR (dendritic cell markers) (left panel). Dendritic cells were identified by double-positive for HLA-DR and CD11c (right panel). Bar graphs show mean ± SEM. Statistical significance: *p <0.05; **p <0.01; ***p <0.001; ****p <0.0001; ns, non-significant (one-way ANOVA with Bonferroni's multiple comparison).

Figure S7

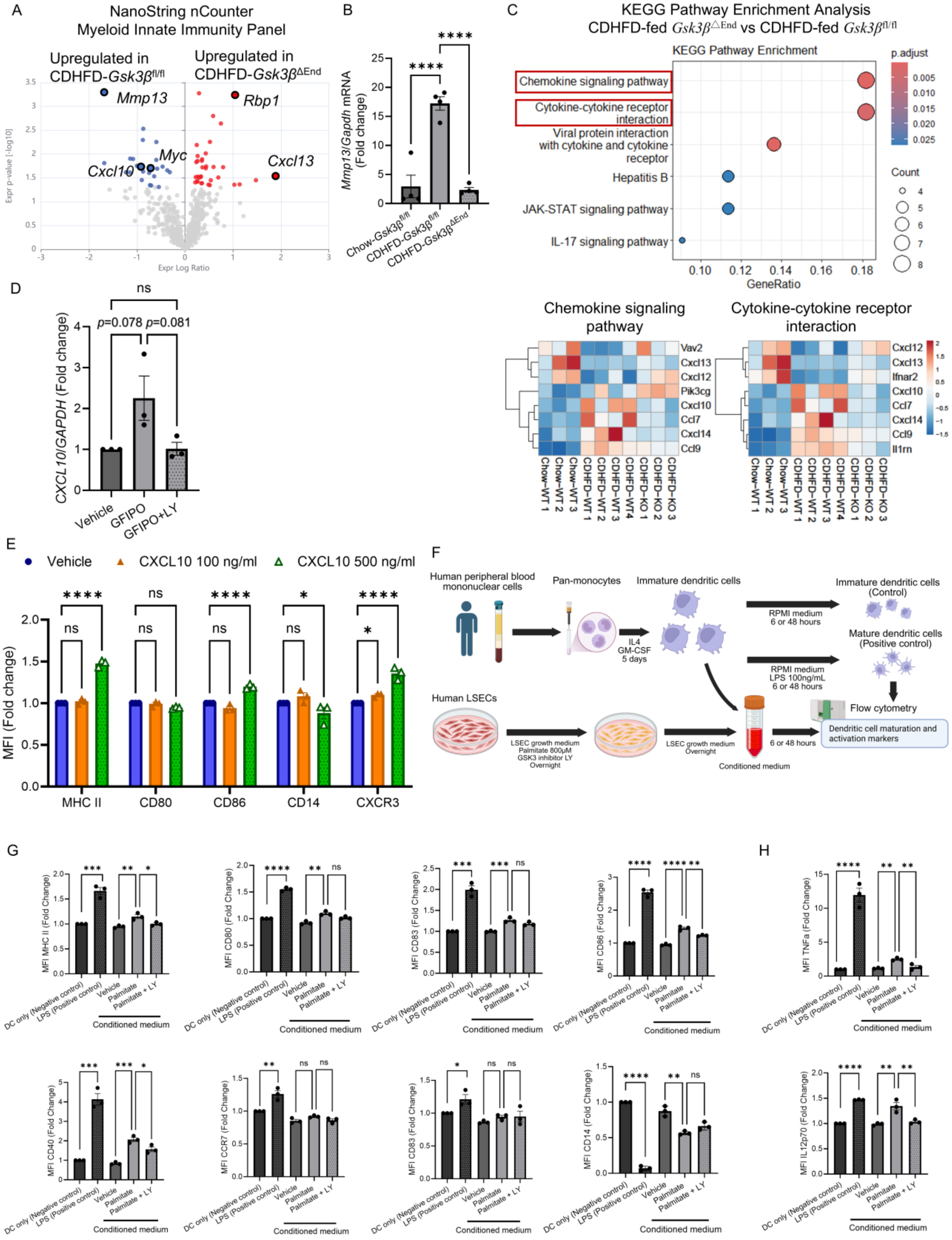
A
NanoString nCounter
Myeloid Innate Immunity Panel
Upregulated in CDHFD-Gsk3β fl/fl
Upregulated in CDHFD-Gsk3β ΔEnd
Mmp13
Rbp1
Myc
Cxcl10
Cxcl13
Expr p-value [-log10]
Expr Log Ratio
B
Mmp13/Gapdh mRNA (Fold change)
Chow-Gsk3β fl/fl
CDHFD-Gsk3β fl/fl
CDHFD-Gsk3β ΔEnd
C
KEGG Pathway Enrichment Analysis
CDHFD-fed Gsk3β ΔEnd vs CDHFD-fed Gsk3β fl/fl
KEGG Pathway Enrichment
Chemokine signaling pathway
Cytokine-cytokine receptor interaction
Viral protein interaction with cytokine and cytokine receptor
Hepatitis B
JAK-STAT signaling pathway
IL-17 signaling pathway
GeneRatio
p.adjust
Count
Chemokine signaling pathway
Cytokine-cytokine receptor interaction
Vav2
Cxcl13
Cxcl12
Pik3cg
Cxcl10
Ccl7
Cxcl14
Ccl9
Ifnar2
Il1rn
Chow-WT 1
Chow-WT 2
Chow-WT 3
CDHFD-WT 1
CDHFD-WT 2
CDHFD-WT 3
CDHFD-WT4
CDHFD-KO 1
CDHFD-KO 2
CDHFD-KO 3
D
CXCL10/GAPDH (Fold change)
ns
p=0.078 p=0.081
Vehicle
GFIPO
GFIPO+LY
E
Vehicle
CXCL10 100 ng/ml
CXCL10 500 ng/ml
MFI (Fold change)
MHC II
CD80
CD86
CD14
CXCR3
F
Human peripheral blood mononuclear cells
Pan-monocytes
Immature dendritic cells
IL4
GM-CSF
5 days
RPMI medium
6 or 48 hours
Immature dendritic cells (Control)
Mature dendritic cells (Positive control)
RPMI medium
LPS 100ng/mL
6 or 48 hours
Flow cytometry
Dendritic cell maturation and activation markers
Human LSECs
LSEC growth medium
Palmitate 800μM
GSK3 inhibitor LY
Overnight
LSEC growth medium
Overnight
Conditioned medium
6 or 48 hours
G
MFI MHC II (Fold Change)
MFI CD80 (Fold Change)
MFI CD83 (Fold Change)
MFI CD86 (Fold Change)
MFI CD40 (Fold Change)
MFI CCR7 (Fold Change)
MFI CD14 (Fold Change)
DC only (Negative control)
LPS (Positive control)
Vehicle
Palmitate
Palmitate + LY
Conditioned medium
H
MFI TNFa (Fold Change)
MFI IL12p70 (Fold Change)

Figure S7. GSK3β inhibition alters cytokines and chemokines-related pathways in hLSECs under lipotoxic stress.

(A) Volcano plot of differential gene expression in LSECs isolated from CDHFD-fed *Gsk3β*$^{\triangle \mathrm{End}}$ vs CDHFD-fed *Gsk3β*$^{\mathrm{fl/fl}}$ mice using the NanoString nCounter Myeloid Innate Immunity Panel. Only samples that passed the Nanostring quality control (QC) assessment were included in the analysis; samples with QC flags were excluded. The x-axis shows the expression log ratio, and y-axis shows the -log10 p-value. The cut-off is $p<0.05$. (B) mRNA expression of *Mmp13* in LSECs isolated from mice. (C) KEGG pathway enrichment analysis of differentially expressed genes (upper panel). The analysis was conducted using R for differential genes ($p < 0.05$) obtained from Nanostring nCounter and nSolver. The horizontal axis shows the gene ratio, while the vertical axis shows the names of enriched pathways. The size of the bubbles shows the number of differentially expressed genes in each pathway (count), and the color gradient represents the p-value. Key pathways highlighted in red. Heatmap of normalized mRNA data for genes in enriched pathways (lower panel). (D) *CXCL10* mRNA expression of PCLS derived from patients treated with MASLD-inducing medium (GFIPO) ± GSK3 inhibitor LY. (E) Flow cytometry analysis of mean fluorescence intensity (MFI) of MHC II, CD80, CD86, and CD14 on human dendritic cells treated with recombinant CXCL10. (F) Schematic representation of human DCs treatment with conditioned medium from palmitate-treated hLSECs. Created in BioRender. Sano, A. (2026) https://BioRender.com/ynnwkwg. (G and H) Flow cytometry analysis of mean fluorescence intensity (MFI) of surface markers (G) and intracellular staining (H) Bar graphs represent the mean ± SEM. Statistical significance: *p <0.05; **p <0.01; ***p <0.001; ***p <0.0001; ns, non-significant (one-way ANOVA with Bonferroni's multiple comparison).

Figure S8

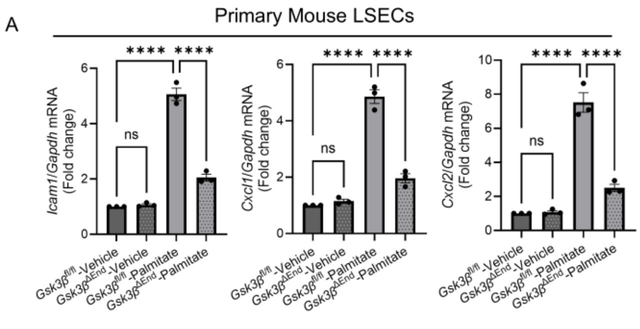

Figure S8. *Gsk3β* deletion attenuates palmitate-induced proinflammatory phenotype in LSECs. (A) mRNA expressions of *Icam1*, *Cxcl1,* and *Cxcl2* in primary mouse LSECs treated with palmitate. Bar graphs represent the mean ± SEM. Statistical significance: *p <0.05; **p <0.01; ***p <0.001; ****p <0.0001; ns, non-significant (one-way ANOVA with Bonferroni's multiple comparison).

Figure S9

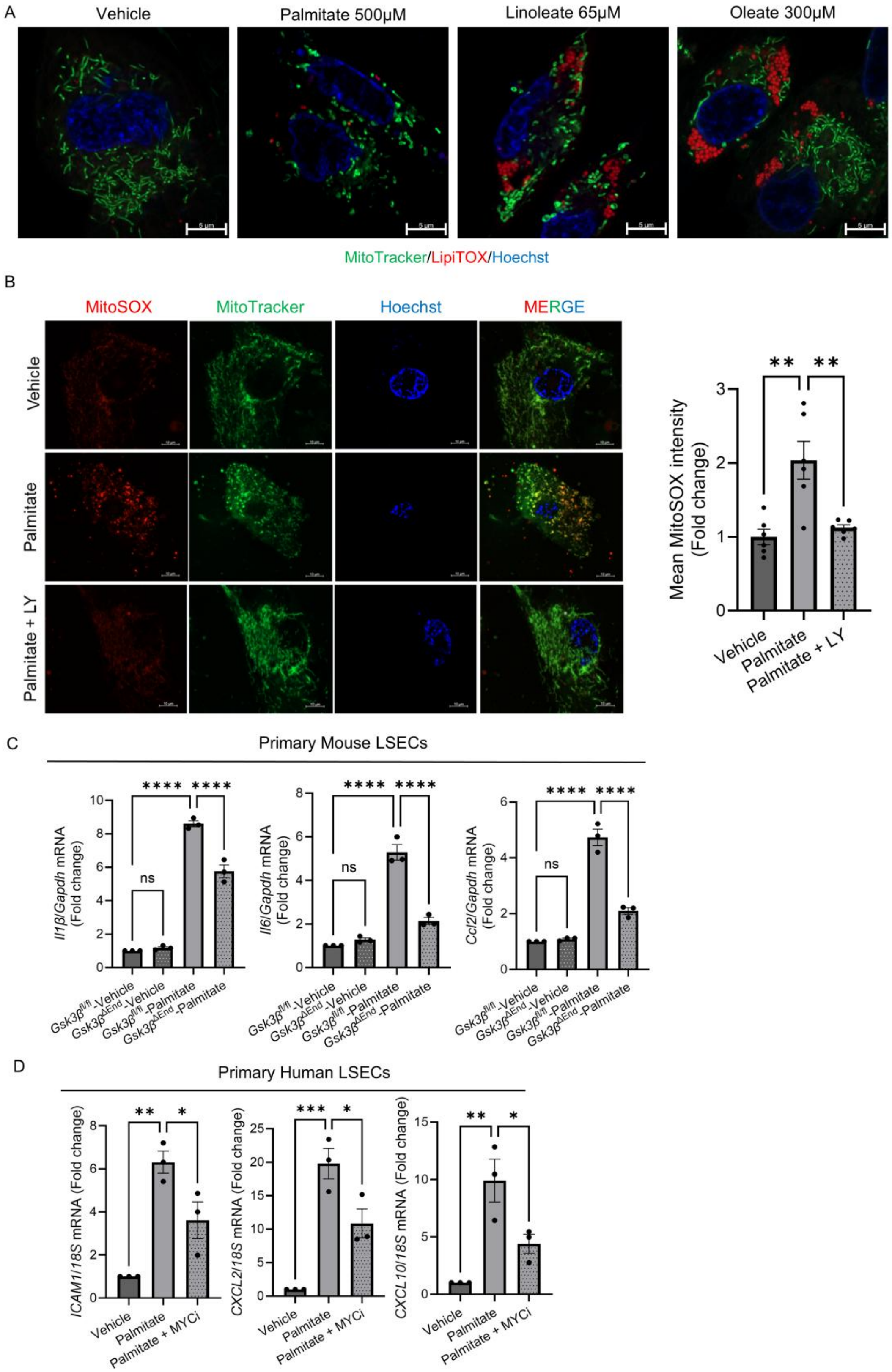
A
Vehicle
Palmitate 500μM
Linoleate 65μM
Oleate 300μM
5 μm
MitoTracker/LipiTOX/Hoechst
B
MitoSOX
MitoTracker
Hoechst
MERGE
Vehicle
Palmitate
Palmitate + LY
Mean MitoSOX intensity (Fold change)
Vehicle
Palmitate
Palmitate + LY
C
Primary Mouse LSECs
Il1β/Gapdh mRNA (Fold change)
Il6/Gapdh mRNA (Fold change)
Ccl2/Gapdh mRNA (Fold change)
ns
$Gsk3\beta^{fl/fl}$-Vehicle
$Gsk3\beta^{\Delta End}$-Vehicle
$Gsk3\beta^{fl/fl}$-Palmitate
$Gsk3\beta^{\Delta End}$-Palmitate
D
Primary Human LSECs
ICAM1/18S mRNA (Fold change)
CXCL2/18S mRNA (Fold change)
CXCL10/18S mRNA (Fold change)
Vehicle
Palmitate
Palmitate + MYCi

Figure S9. Fatty acids treatments in LSECs induce mitochondrial morphological alterations, ROS generation, and NfκB downstream target upregulation via a *Gsk3β* dependent mechanism. (A) Representative confocal images of hLSECs stained with MitoTracker (green) and LipiTOX (red). hLSECs were incubated overnight with 500 μM palmitate, 65 μM linoleate, or 300 μM oleate for overnight. Scale bars = 5 μm. (B) Representative confocal images of hLSECs stained with MitoTracker (green) and MitoSOX (red). hLSECs were incubated overnight with 800 μM palmitate for overnight. Scale bars = 5 μm. The quantification is shown in the right panel. (C) mRNA expression of *Il1β*, *Il6*, and *Ccl2* in LSECs isolated from mice and treated with palmitate (500 μM, overnight). (D) mRNA expression of *ICAM1*, *CXCL2*, and *CXCL10* in hLSECs treated with palmitate with and without the MYC inhibitor (MYCi). Bar graphs represent the mean ± SEM. Statistical significance: *p <0.05; **p <0.01; ***p <0.001; ****p <0.0001; ns, non-significant (one-way ANOVA with Bonferroni's multiple comparison).

Figure S10

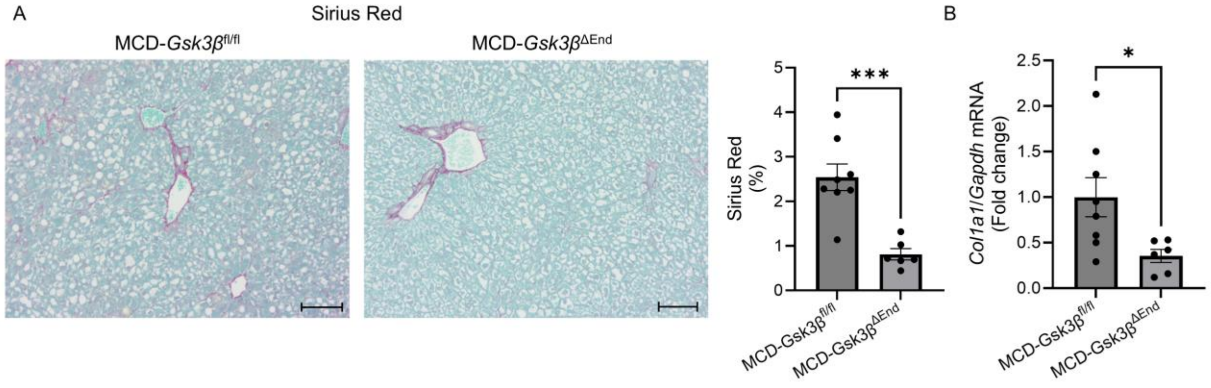

Figure S10. Endothelial cell-specific *Gsk3β* deletion attenuates liver fibrosis in murine MASH. (A) Representative images of MCD-fed *Gsk3β*$^{\triangle \text{End}}$ and *Gsk3β*$^{\text{fl/fl}}$ mice showing Sirius Red staining (left panel). Positive areas were quantified in 5 random 10x microscopic fields and averaged for each animal. (right panel) Scale bar is 100μm. (B) *Col1a1* mRNA expression of whole livers from MCD-fed *Gsk3β*$^{\triangle \text{End}}$ and *Gsk3β*$^{\text{fl/fl}}$ mice. Bar graphs represent the mean ± SEM. Statistical significance: *p <0.05; **p <0.01; ***p <0.001; ****p <0.0001; ns, non-significant (unpaired t-test).

4. References

1. Hilscher MB, Sehrawat T, Arab JP, Zeng Z, Gao J, Liu M, et al. Mechanical Stretch Increases Expression of CXCL1 in Liver Sinusoidal Endothelial Cells to Recruit Neutrophils, Generate Sinusoidal Microthombi, and Promote Portal Hypertension. *Gastroenterology*. 2019;157(1):193-209.e9.
2. Pitulescu ME, Schmidt I, Benedito R, and Adams RH. Inducible gene targeting in the neonatal vasculature and analysis of retinal angiogenesis in mice. *Nat Protoc*. 2010;5(9):1518-34.
3. Furuta K, Guo Q, Pavelko KD, Lee JH, Robertson KD, Nakao Y, et al. Lipid-induced endothelial vascular cell adhesion molecule 1 promotes nonalcoholic steatohepatitis pathogenesis. *J Clin Invest*. 2021;131(6).
4. Zhao P, Sun X, Chaggan C, Liao Z, In Wong K, He F, et al. An AMPK-caspase-6 axis controls liver damage in nonalcoholic steatohepatitis. *Science*. 2020;367(6478):652-60.
5. Monvoisin A, Alva JA, Hofmann JJ, Zovein AC, Lane TF, and Iruela-Arispe ML. VE-cadherin-CreERT2 transgenic mouse: a model for inducible recombination in the endothelium. *Dev Dyn*. 2006;235(12):3413-22.
6. Matsumoto M, Hada N, Sakamaki Y, Uno A, Shiga T, Tanaka C, et al. An improved mouse model that rapidly develops fibrosis in non-alcoholic steatohepatitis. *International journal of experimental pathology*. 2013;94(2):93-103.
7. Matthews DR, Hosker JP, Rudenski AS, Naylor BA, Treacher DF, and Turner RC. Homeostasis model assessment: insulin resistance and beta-cell function from fasting plasma glucose and insulin concentrations in man. *Diabetologia*. 1985;28(7):412-9.
8. Kleiner DE, Brunt EM, Van Natta M, Behling C, Contos MJ, Cummings OW, et al. Design and validation of a histological scoring system for nonalcoholic fatty liver disease. *Hepatology*. 2005;41(6):1313-21.
9. Tomita K, Kohli R, MacLaurin BL, Hirsova P, Guo Q, Sanchez LHG, et al. Mixed-lineage kinase 3 pharmacological inhibition attenuates murine nonalcoholic steatohepatitis. *JCI Insight*. 2017;2(15).
10. Schindelin J, Arganda-Carreras I, Frise E, Kaynig V, Longair M, Pietzsch T, et al. Fiji: an open-source platform for biological-image analysis. *Nature Methods*. 2012;9(7):676-82.
11. Chaudhry A, Shi R, and Luciani DS. A pipeline for multidimensional confocal analysis of mitochondrial morphology, function and dynamics in pancreatic β-cells. *bioRxiv*. 2019:687749.
12. de Graaf IA, Olinga P, de Jager MH, Merema MT, de Kanter R, van de Kerkhof EG, et al. Preparation and incubation of precision-cut liver and intestinal slices for application in drug metabolism and toxicity studies. *Nat Protoc*. 2010;5(9):1540-51.
13. Pearen MA, Lim HK, Gratte FD, Fernandez-Rojo MA, Nawaratna SK, Gobert GN, et al. Murine Precision-Cut Liver Slices as an Ex Vivo Model of Liver Biology. *J Vis Exp*. 2020(157).
14. Li M, Larsen FT, van den Heuvel MC, Gier K, Gorter AR, Oosterhuis D, et al. Metabolic Dysfunction-Associated Steatotic Liver Disease in a Dish: Human Precision-Cut Liver Slices as a Platform for Drug Screening and Interventions. *Nutrients*. 2024;16(5):626.
15. Guo Q, Furuta K, Aly A, and Ibrahim SH. Isolation and Characterization of Mouse Primary Liver Sinusoidal Endothelial Cells. *J Vis Exp*. 2021(178).
16. Goldsborough T, O'Callaghan A, Inglis F, Leplat L, Filby A, Bilen H, et al. A novel channel invariant architecture for the segmentation of cells and nuclei in multiplexed images using InstanSeg. *bioRxiv*. 2024:2024.09.04.611150.

17. Virtanen P, Gommers R, Oliphant TE, Haberland M, Reddy T, Cournapeau D, et al. SciPy 1.0: fundamental algorithms for scientific computing in Python. *Nature Methods*. 2020;17(3):261-72.
