# Supplementary material for: Endothelial cell glycogen synthase kinase 3β promotes lipotoxic endotheliopathy and liver inflammation in MASH
Source: JCI Insight. 2026 May 5;11(12):e202552. doi: 10.1172/jci.insight.202552 (PMC13313496; doi:10.1172/jci.insight.202552)
Supplement: Unedited blot and gel images [file jciinsight-11-202552-s121.pdf]

Uncropped-unedited blots
Figure 1B

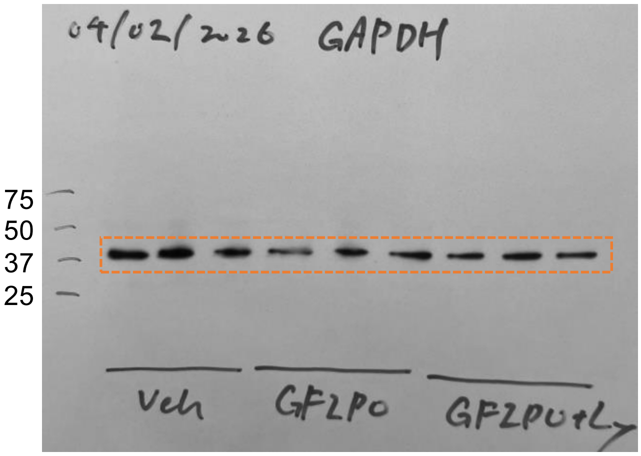

GAPDH

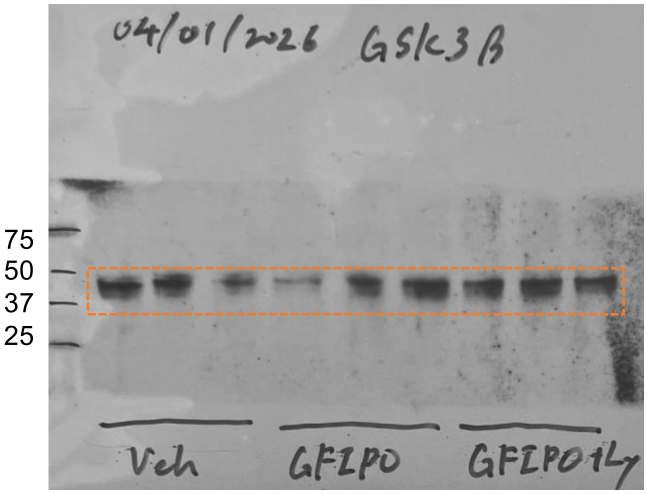

GSK3β

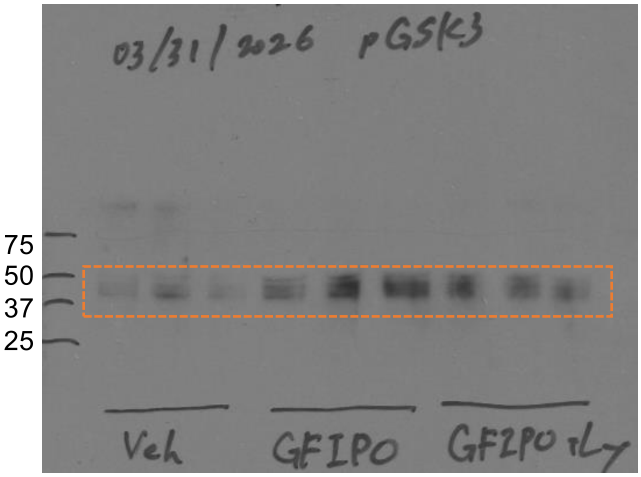

pGSK3α/β

Uncropped-unedited blots
Figure 1E

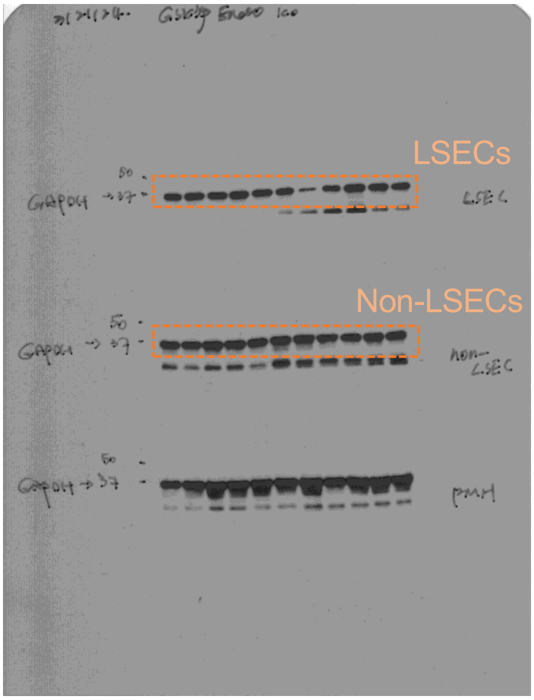

Gapdh

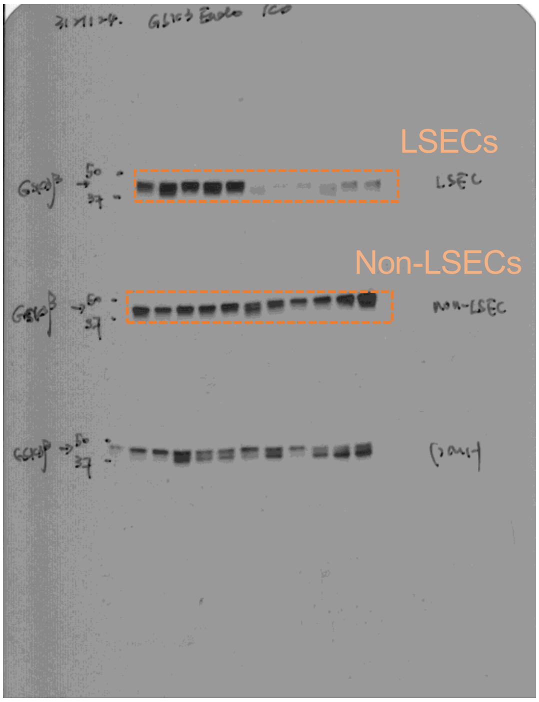

Gsk3b

Uncropped-unedited blots
Figure 5I

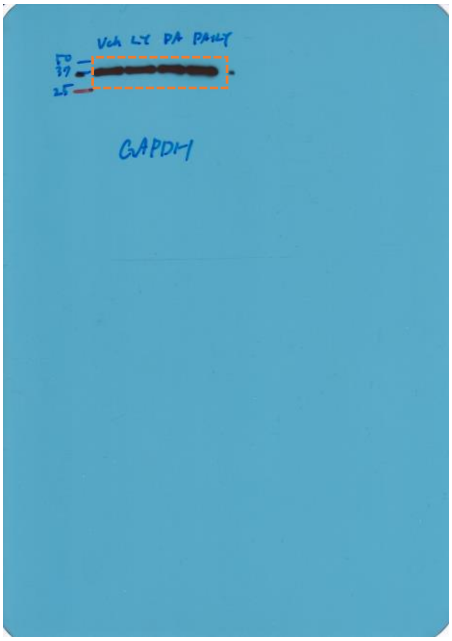

GAPDH

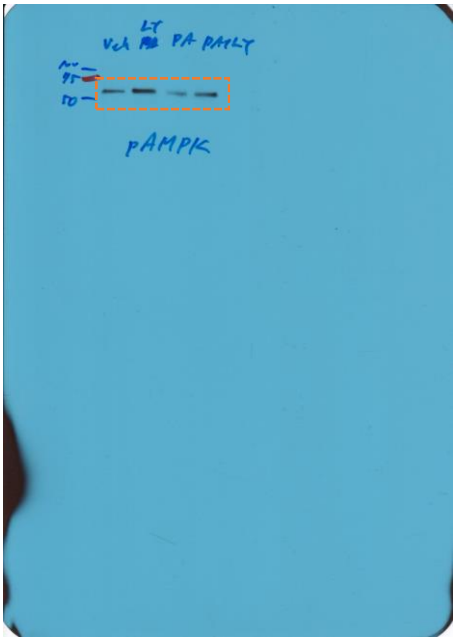

pAMPK

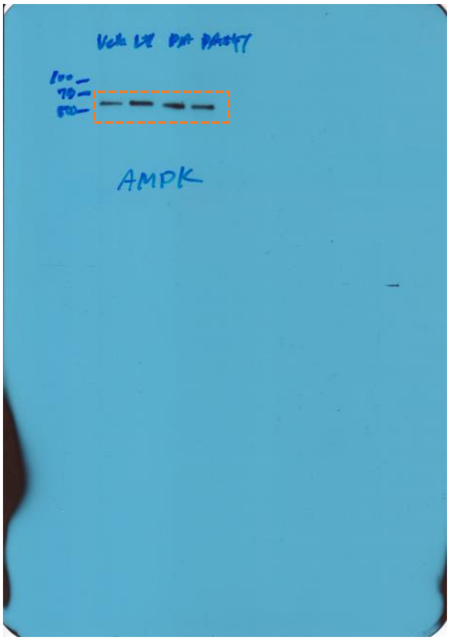

Total AMPK

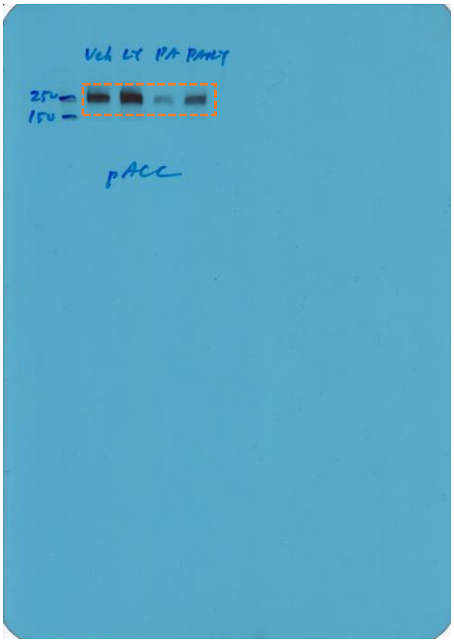

pACC

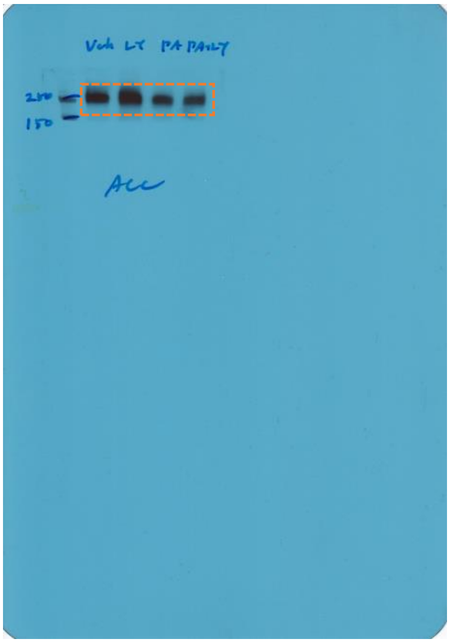

Total ACC

Uncropped-unedited blots
Figure 6S

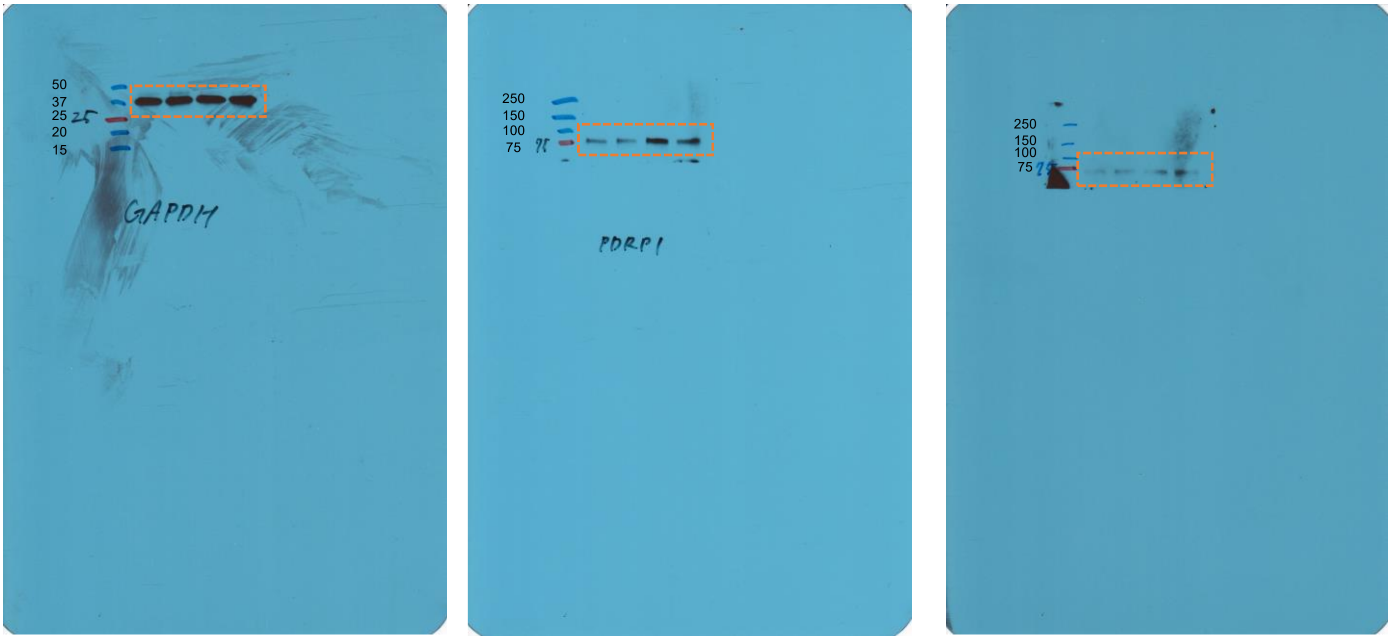

GAPDH

pDRP1

Total DRP1
